# Biological Properties of Vitamins of the B-Complex, Part 1: Vitamins B_1_, B_2_, B_3_, and B_5_

**DOI:** 10.3390/nu14030484

**Published:** 2022-01-22

**Authors:** Marcel Hrubša, Tomáš Siatka, Iveta Nejmanová, Marie Vopršalová, Lenka Kujovská Krčmová, Kateřina Matoušová, Lenka Javorská, Kateřina Macáková, Laura Mercolini, Fernando Remião, Marek Máťuš, Přemysl Mladěnka, on behalf of the OEMONOM

**Affiliations:** 1 Department of Pharmacology and Toxicology, Faculty of Pharmacy in Hradec Králové, Charles University, Akademika Heyrovského 1203, 500 05 Hradec Kralove, Czech Republic; hrubsam@faf.cuni.cz (M.H.); voprsalova@faf.cuni.cz (M.V.); mladenkap@faf.cuni.cz (P.M.); 2 Department of Pharmacognosy, Faculty of Pharmacy in Hradec Králové, Charles University, Akademika Heyrovského 1203, 500 05 Hradec Kralove, Czech Republic; siatka@faf.cuni.cz (T.S.); macakovak@faf.cuni.cz (K.M.); 3 Department of Biological and Medical Sciences, Faculty of Pharmacy in Hradec Králové, Charles University, Akademika Heyrovského 1203, 500 05 Hradec Kralove, Czech Republic; ivetanajmanova@seznam.cz; 4 Department of Analytical Chemistry, Faculty of Pharmacy in Hradec Králové, Charles University, Akademika Heyrovského 1203, 500 05 Hradec Kralove, Czech Republic; lenka.kujovska@fnhk.cz; 5 Department of Clinical Biochemistry and Diagnostics, University Hospital Hradec Králové, Sokolská 581, 500 05 Hradec Kralove, Czech Republic; katerina.matousova@fnhk.cz (K.M.); lenka.javorska@fnhk.cz (L.J.); 6 Research Group of Pharmaco-Toxicological Analysis (PTA Lab), Department of Pharmacy and Biotechnology (FaBiT), Alma Mater Studiorum, University of Bologna, Via Belmeloro 6, 40126 Bologna, Italy; laura.mercolini@unibo.it; 7 UCIBIO—Applied Molecular Biosciences Unit, REQUINTE, Toxicology Laboratory, Biological Sciences Department Faculty of Pharmacy, University of Porto, 4050-313 Porto, Portugal; remiao@ff.up.pt; 8 Associate Laboratory i4HB—Institute for Health and Bioeconomy, Faculty of Pharmacy, University of Porto, 4050-313 Porto, Portugal; 9 Department of Pharmacology and Toxicology, Faculty of Pharmacy, Comenius University Bratislava, Odbojárov 10, 83232 Bratislava, Slovak Republic

**Keywords:** thiamine, riboflavin, niacin, pantothenic acid, essential

## Abstract

This review summarizes the current knowledge on essential vitamins B_1_, B_2_, B_3_, and B_5_. These B-complex vitamins must be taken from diet, with the exception of vitamin B_3_, that can also be synthetized from amino acid tryptophan. All of these vitamins are water soluble, which determines their main properties, namely: they are partly lost when food is washed or boiled since they migrate to the water; the requirement of membrane transporters for their permeation into the cells; and their safety since any excess is rapidly eliminated via the kidney. The therapeutic use of B-complex vitamins is mostly limited to hypovitaminoses or similar conditions, but, as they are generally very safe, they have also been examined in other pathological conditions. Nicotinic acid, a form of vitamin B_3_, is the only exception because it is a known hypolipidemic agent in gram doses. The article also sums up: (i) the current methods for detection of the vitamins of the B-complex in biological fluids; (ii) the food and other sources of these vitamins including the effect of common processing and storage methods on their content; and (iii) their physiological function.

## Contents

1. Introduction22. Thiamine—Vitamin B_1_22.1. Introduction and Properties22.2. Sources of Thiamine32.3. Pharmacokinetics of Thiamine72.4. Physiological Function of Thiamine92.5. Thiamine Deficiency102.6. Pharmacological Use of Thiamine122.7. Toxicity of Thiamine172.8. Drug-Vitamin Interactions Associated with Thiamine Deficiency173. Riboflavin—Vitamin B_2_173.1. Introduction and Properties173.2. Sources of Riboflavin173.3. Pharmacokinetics of Riboflavin203.4. Physiological Functions of Riboflavin233.5. Riboflavin Deficiency243.6. Analytical Determination253.7. Pharmacological Use of Riboflavin263.8. Toxicity of Riboflavin263.9. Drug Interactions Affecting Pharmacokinetics and Interfering with Physiological Function of Riboflavin264. Niacin—Vitamin B_3_274.1. Introduction and Properties274.2. Sources of Niacin274.3. Pharmacokinetics of Niacin314.4. Physiological Functions of Niacin334.5. Niacin Deficiency384.6. Pharmacological Use of Niacin384.7. Toxicity of Niacin445. Pantothenic Acid—Vitamin B_5_455.1. Introduction and Properties455.2. Sources of Pantothenic Acid455.3. Physiological Function of Pantothenic Acid485.4. Pharmacokinetics of Pantothenic Acid495.5. Pantothenic Acid Deficiency535.6. Pharmacological Use of Pantothenic Acid556. Conclusions58References59

## 1. Introduction

At the turn of the 20th century, investigation of nutritional requirements yielded crucial findings. Vital amines, or “vitamines” at that time, have been described, at first thought to be merely 2 compounds: a lipid-soluble vitamine A and a water-soluble vitamine B. Further research has shown that “vitamine B” is a complex of a wide range of compounds that only share their solubility. In this review, a concise summary of their discovery, sources, physiological role, pharmacological use, potential toxicity, and interactions of individual vitamins belonging to “B-complex” is provided. The first part is concerned with vitamins B_1_—thiamine, B_2_—riboflavin, B_3_—niacin, and B_5_—pantothenic acid, and the second part will provide information on the remaining members of the “B-complex”.

## 2. Thiamine—Vitamin B_1_

### 2.1. Introduction and Properties

**Figure 1:**
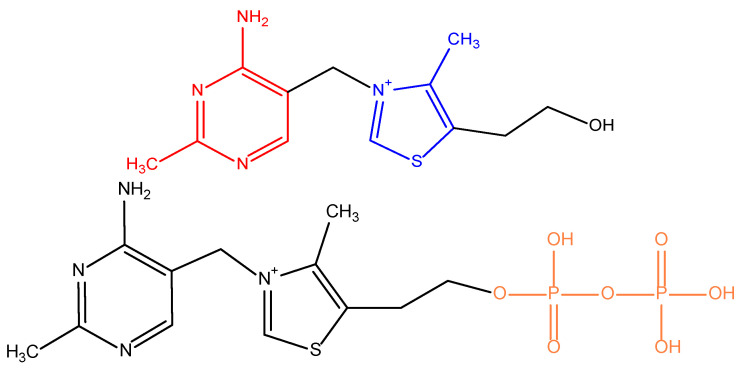
Structure of thiamine (vitamin B_1_ (upper)) and thiamine pyrophosphate (TPP (bottom)).

In 1897, vitamin B_1_ was discovered as the first vitamin, at that time called “vitamine”. It was isolated in 1926 and synthesized 10 years later. This substance contains an amino group, and, because of that, it was called “vital amine“ (“an amine of life“), which, as mentioned, initially gave the name to the whole group of micronutrients—formerly vitamines, currently known as vitamins [1]. Vitamin B_1_ was also formerly known as “aneurin“ because it prevented neurological symptoms of hypovitaminosis. Currently, rather, it is known as thiamine. This term reflects the presence of both sulfur and an amino group in the molecule. Thiamine is a water-soluble compound composed of pyrimidine (or precisely 4-amino-2-methylpyrimidine) and thiazole/4-methyl-5-(2-hydroxyethyl)-thiazolium/rings, which are linked by a methylene bridge (Figure 1) [2,3,4,5].

Free thiamine is a base that is stable at acidic pH, but it is decomposed by ultraviolet light and gamma irradiation, and it is susceptible to heat. In alkaline solutions, and in the presence of oxidizing agents, thiamine is converted into thiochrome, a fluorescent substance, that is used to determine the vitamin levels in food and drugs [3].

In pharmaceutical products, it is used usually in the form of solid, water soluble, thiazolinium salts, e.g., of thiamine hydrochloride or thiamine mononitrate. There are also synthetic lipophilic derivatives of the vitamin, e.g., allithiamins, which can pass biological membranes more easily [6].

In an organism, thiamine may occur in several forms, mainly esters, e.g., thiamine monophosphate (TMP), thiamine diphosphate (also sometimes called thiamine pyrophosphate/TPP/ or cocarboxylase; see Figure 1), and thiamine triphosphate (TTP). Recently, other forms, such as adenosine thiamine diphosphate and adenosine thiamine triphosphate, have also been discovered in humans [6,7].

### 2.2. Sources of Thiamine

Plants, fungi, and most bacteria synthetize thiamine, whereas other organisms (all animals, as well as many prokaryotes and unicellular eukaryotes) rely on external intake for thiamine supply [8,9,10,11,12,13,14]. Hence, in humans, thiamine is an essential vitamin that needs to be continuously supplied by the diet [15]. The most common nutritional sources of thiamine for humans are whole grains, bread, meat (especially pork), and pulses, while milled wheat flour, polished rice, vegetables, and fruits are less useful [11,16,17,18,19]. Nuts are also rich in thiamine [20,21]. Amounts of thiamine in some selected foodstuffs are shown in Table 1.

Several food products, however, also contain so-called anti-thiamine factors (e.g., thiaminases and thiamine antagonists) that inactivate or block the absorption of thiamine. Regular consumption of those foods is thought to be implicated in the development of thiamine deficiency [22,23,24,25]. Thiaminases, heat-labile enzymes that cleave thiamine and render it inactive, are found in certain raw and fermented fish, shellfish, African silkworm larvae, ferns (e.g., *Pteridium aquilinum*, *Marsilea drummondii*, and *Equisetum arvense*), and some bacteria (e.g., *Bacillus* and *Clostridium* species). Their anti-thiamine activity can be eliminated by cooking. Heat-stable thiamine antagonists include phenolic compounds (especially with ortho-dihydroxy groups, e.g., tannins, caffeic acid, and chlorogenic acid) and methylxanthines, such as caffeine, theophylline, and theobromine, occurring in some foods of plant origin, such as blueberries, betel nuts, coffee, and tea. They react with thiamine to yield the nonabsorbable thiamine disulfide. The destructive process of these compounds can be prevented, e.g., by ascorbic acid (playing a protective role if it is consumed together with thiamine), and tartaric acid and citric acid (acidifying agents), all present in many vegetables and fruits, or by delaying contact between thiamine and tannins. The latter can be achieved by consuming tea hours after a thiamine-containing meal instead of during the meal [11,15,22,23,24,26,27,28,29,30,31,32,33,34,35,36,37,38,39,40,41,42,43,44,45,46]. Trace amounts of thiamine antimetabolites, such as oxythiamine, are formed from thiamine through cooking under acidic conditions at 100 °C. Oxythiamine can be accumulated in end-stage renal disease patients, which leads to inhibition of transketolase with possible negative impact on those patients [2,47]. Lastly, the cyanogenic glycosides (linamarin and lotaustralin), which are present in the cassava plant (*Manihot esculenta*), bind the sulfur in thiamine molecule, splitting this vitamin into its pyrimidine and thiazole moieties; therefore, the vitamin becomes inactive [48]. Thiamine is also very sensitive to sulfites, especially at a high pH [16,22,49,50]. The sulfite-induced cleavage of thiamine is the cause of large losses of the vitamin in vegetables, fruits, and minced meats where sulfites and bisulfites are used as preservatives [51]. Thiamine is cleaved, as well, by residual chlorine in the cooking water [22,52,53]. The presence of copper ions accelerates decomposition of thiamine, too [22,54].

Primary and secondary food processing may unfavorably affect the thiamine content [22,24,55]. Regarding cereals, all grains are structurally composed of endosperm (80–85%), germ (2–3%), and bran (13–17%) [56]. Thiamine, as in some other micronutrients, is not evenly distributed throughout the grain. The bran portion and germ, which are removed during milling, and refining (wheat and maize) or polishing (rice) of grains, are much richer in thiamine than the endosperm [57]. The thiamine content in milled refined wheat flour is reduced by 40–80% in comparison to whole wheat flour [22,56,57,58]. Similarly, thiamine losses in white rice are 75–82% in contrast to brown rice [11,22,59,60,61]. Amounts of thiamine in various maize milled products decrease by 36–82% as compared to whole kernels [62,63].

Thiamine stability is good in acidic solutions (e.g., in acidic fruit juices and drinks [22,51]), even when heated [22,24,53,64]. It is very susceptible to decomposition by several factors, such as neutral and alkaline conditions, heat, oxidizing, and reducing agents, and radiation [16,17,24,51,53,54,64,65,66,67,68,69]. Thiamine leaches into the water, owing to its solubility, and will be lost in any soaking or cooking water that is thrown away, as well as being destroyed by heating during culinary processes, in dependence on a type of food, a method used, temperature, and duration [21,22,53,70,71,72,73,74,75,76,77,78,79,80,81,82,83,84,85,86,87,88,89].

Boiling, stewing, roasting, and frying lead to thiamine losses in meat. The respective losses from pork were 70%, 38–55%, 18–40%, and 10–50%, while, from beef, the losses were 60–80%, 55–68%, 12–38%, and 55%, respectively [53,70,100,108]. Consumption of meat and other food with cooking liquids, such as water added or exuding juices, is hindering the losses of thiamine for the reason mentioned in the previous paragraph. For example, thiamine contents decrease by 33–40% in boiled beef with soup, and by 30% in braised pork with gravy, whereas they decrease by 60–80% and 55% solely in beef and pork, [53,70,100]. Fried breaded meat, such as pork, chicken, lamb, and beef, contain 15–40% more thiamine than variants with no breading [70]. Meat with a lower initial fat content loses more water during thermal culinary processing, resulting in more thiamine leaching out, and so the fat content might be a factor with a positive influence on the vitamin retention in the course of heat treatment of meat [74].

Thiamine content in rice is reduced due to washing before cooking, boiling, stewing, baking, and frying by 37–50%, 25–52%, 20–25%, 15–30%, and 10–30%, respectively [15,22,53,70,71,72,73,82,109,110]. Steaming, boiling, baking, and frying of potatoes cause 15%, 20% (with peel; 25% in peeled potatoes), 15–20%, and 20–40% thiamine losses, respectively [70,71]. Generally, steaming is preferred to boiling because it hinders thiamine losses due to its leaching into the water [111,112]. Decreases in thiamine level in pasta, boiled and not drained, boiled and drained, baked, and boiled, drained and baked, amounts 20%, 35%, 20%, and 45%, respectively [72]. In legumes, cooking brings about 20–25% thiamine loss, while pressure cooking brings about thiamine loss 56–58% [70,74]. The practice of adding sodium bicarbonate to peas or beans for retention of their colors in cooking or canning results in a large decline of thiamine due to the alkaline environment [22,113].

Losses of thiamine during the baking of white bread are between 15 to 37% [51,114]. The content of thiamine decreases more in wheat than in rye baking [115]. Use of baking powder in cake mixtures brings about significant thiamine decrease of 50% or higher owing to poor stability of thiamine under alkaline conditions [74].

Scrambled, hard cooked, and fried eggs lose 5%, 20%, and 30% thiamine during cooking, respectively [70]. Heat-induced reduction in thiamine in milk is normally up to 10%, 5–15%, 30–40%, and 30–50% for pasteurized, ultra-heat treated, sterilized, and evaporated milk, respectively [22,51,87,116,117], and it is lower in whole milk than that in low-fat and skimmed milk [74]. Donor human milk can be processed using Holder pasteurization (62.5 °C for 30 min) or a current retort-processed alternative (121 °C, 15 PSI for 5 min), which increases the shelf-life. Thiamine content is unaffected by the former but decreased by about 42% in the latter. Those losses are clinically significant, and fortification may be needed if shelf-stable donor milk is a long-term feeding choice [118].

Effects of extrusion processing [119,120], gamma irradiation as a conservation technique [68,121,122,123,124], canning [125,126,127], freezing [89,126], storage conditions [81,86,105,126,128,129,130,131], and edible seed germination [90,94,95,96,132,133] on thiamine content in foods have also been reported.

A range of food ingredients has been shown to affect the stability of thiamine. Starch and proteins (such as egg albumin and casein) are protective. Mannitol, inositol, and fructose, in contrast to glucose, can retard the rate of destruction of thiamine during heat processing [40,51,65]. Allicin in garlic, when the bulb is cut or crushed, reacts with thiamine to form allithiamine, a more lipophilic form with improved absorption [22,134,135,136,137], which will be discussed later.

Parboiling improves the quality of milled rice and is an example of a food processing method which, in contrast to those aforementioned, can increase thiamine content of polished rice—rice in the husk is soaked in water, steamed, and dried before milling [23,138,139,140,141]. Steaming causes thiamine and other nutrients to migrate from the outer layers into the endosperm, so that removing the outer layers by milling does not denude the grain of thiamine—parboiled rice may be milled to a high degree and yet retain enough thiamine [22,23,142,143]. Losses of thiamine in parboiled and non-parboiled polished rice are 33–40% and 75–85%, respectively, in comparison to brown rice [22,144].

Although food processing has mostly a negative effect on the content of thiamine, it should be emphasized that processing may occasionally be necessary for minimizing harmful constituents, such as arsenic. This is the case of rice, which is an exception among cereals since its inorganic arsenic content can reach one order of magnitude higher levels than in wheat and barley. The reason is that the plant is able to take up and translocate inorganic arsenic to the grain. Moreover, the semiaquatic anaerobic growing environment in paddy fields favors root uptake of arsenic [145,146]. In rice grains, arsenic accumulates especially in husk and bran and, to a much lesser extent, in endosperm [147,148,149,150]. It has been clearly demonstrated that inorganic arsenic may lead to a plethora of pathological conditions [151,152,153,154,155,156,157,158,159,160,161,162]. Polishing and rinsing before cooking and boiling with excess water which is then discarded lead to an efficient reduction of arsenic but, at the same time, also to the aforementioned losses of vitamin B_1_ [149,150,151,156,158,163,164,165,166,167,168,169]. In fact, in respect of arsenic, resulting recommendations on preparation and consumption of rice lately prefer a safety point of view to the nutritional one, especially in some population groups, such as infants and toddlers [151,154,156].

Thiamine is widespread in nature, but only in relatively small quantities. Since its extraction from natural sources would not be economically profitable, it has to be manufactured by chemical synthesis [59]. All industrially-relevant syntheses of thiamine use a pyrimidine intermediate called Grewe diamine (4-amino-5-aminomethyl-2-methylpyrimidine) as the key building element. Acrylonitrile or malononitrile are the common starting materials for the synthesis of this intermediate. The thiazole ring is then linearly constructed on the preformed pyrimidine moiety (Grewe diamine) in three chemical steps to obtain thiamine [59,170]. Microbial fermentations have met limited success because of thiamine biosynthetic pathway is very complex, and its regulation is complicated. Therefore, major metabolic engineering breakthroughs will be needed because thiamine fermentation will require high yields to become competitive to the current low-cost chemical manufacturing process [171].

In many countries, mandatory or voluntary fortification of food products with thiamine provides an artificial dietary source of the vitamin, compensating for its often-insufficient intake from foods containing it naturally [12,23,92,172,173,174,175,176,177,178,179]. Fortified foods contribute to about half of the total thiamine consumed in some high-income countries [175,180]. Wheat flour, maize flour, and rice are foods commonly fortified with thiamine [61,92,181,182,183,184,185,186]. Biofortification, the augmentation of natural micronutrient levels in crops through breeding and genetic engineering, offers a long-term sustainable approach to increase the amount of dietary thiamine for people who rely on staple crops for most of their caloric intake. Despite efforts made, none have undergone human feeding trials, and further research is needed [92,172,187,188,189,190,191].

### 2.3. Pharmacokinetics of Thiamine

Ingested thiamine from dietary sources is readily absorbed in the proximal small intestine (upper jejunum). Absorption is dependent on the dose and involves two mechanisms: carrier-mediated active transport, as long as oral intake is less than 5 mg; and passive diffusion, at higher doses [192].

Most of dietary thiamine occurs in phosphorylated forms, and intestinal phosphatases hydrolyze them to free thiamine before the vitamin is absorbed [193].

**Figure 2:**
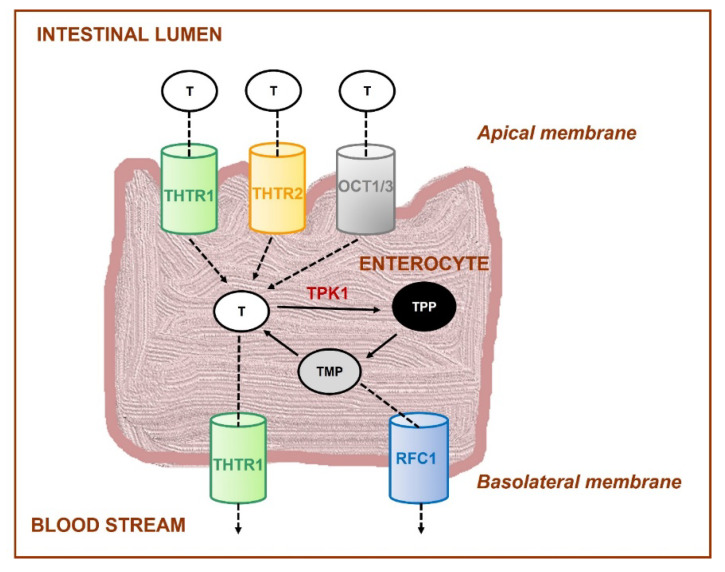
Intestinal uptake of thiamine. THTR1 carrier (encoded by SLC19A2 gene); THTR2 carrier (encoded by SLC19A3 gene); OCT1/3, organic cation-transporter 1/3; RFC1, reduced folate carrier (encoded by SLC19A1 gene); T, thiamine; TMP, thiamine monophosphate; TPP, thiamine pyrophosphate; TPK1, thiamine phosphokinase.

Free thiamine is taken up into intestinal mucosal cells (enterocytes), mainly by two saturable and high affinity transporters, THTR1 (encoded by SLC19A2 gene) and THTR2 (encoded by SLC19A3 gene) (Figure 2). THTR1 transporter is located on the apical brush-border membrane and basolateral membrane, while THTR2 is found only on the apical surface [194,195,196,197]. Moreover, other transporters (e.g., organic cation-transporters OCT1,3), which are not specific to thiamine, are involved in this process, as well [198].

Humans also obtain vitamin B_1_ from bacterial microbiota of the large intestine, where both thiamine and TPP are synthesized. Thiamine is taken up into colonocytes by similar carrier-mediated mechanism, such as in enterocytes, and TPP enters these cells by the human colonic thiamine pyrophosphate transporter (encoded by SLC44A44 gene).

Transport from intestine into the portal circulation through the basolateral membrane is mediated by the THTR1 carrier. Another transporter, called the reduced folate carrier (RFC1 is encoded by SLC19A1 gene), is less important since it transports thiamine monophosphate (TMP) but not free thiamine [193,198].

The concentrations of total thiamine (free thiamine and its phosphorylated forms) in whole blood are in the range of 75–200 nmol/L. Once in the circulation, thiamine crosses the erythrocyte membrane by the means of THTR1 and THTR2 transporters. In the TMP transport, RFC1 is again implicated. Inside the red blood cell, phosphorylation occurs and TPP is formed. The result is that 80% of thiamine in the blood is located in erythrocytes in the form of TPP. Only low concentrations of the vitamin are present in the plasma, both as free thiamine, TMP and protein bound TPP [198,199].

**Figure 3:**
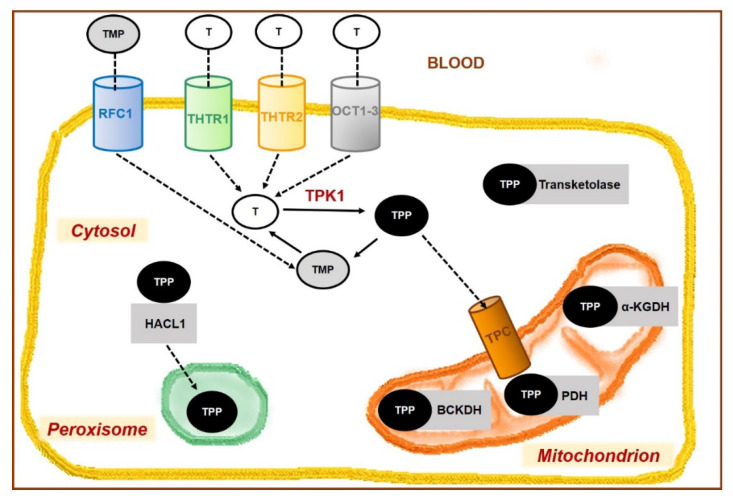
Cellular uptake of thiamine. THTR1 carrier (encoded by SLC19A2 gene); THTR2 carrier (encoded by SLC19A3 gene); OCT1-3, organic cation-transporters 1–3; RFC-1, reduced folate carrier (encoded by SLC19A1 gene); TPC, thiamine pyrophosphate carrier (encoded by SLC25A19 gene); T, thiamine; TPP, thiamine pyrophosphate; TMP, thiamine monophosphate; TPK1, thiamine phosphokinase; BCKDH, branched-chain α-ketoacid dehydrogenase; PDH, pyruvate dehydrogenase; α-KGDH, α-ketoglutarate dehydrogenase; HACL1, 2-hydroxyacyl-CoA lyase 1.

From blood, thiamine is taken up into cells of various tissues by previously mentioned transporters, i.e., THTR1, THTR2, and OCT, while RFC1 allows TMP transfer again (Figure 3). Both carriers THTR1 and THTR2 are highly expressed in the cells of various organs, e.g., skeletal muscle, heart, kidney, liver, pancreas, brain, placenta, and adipose tissue [5,200].

**Figure 4:**
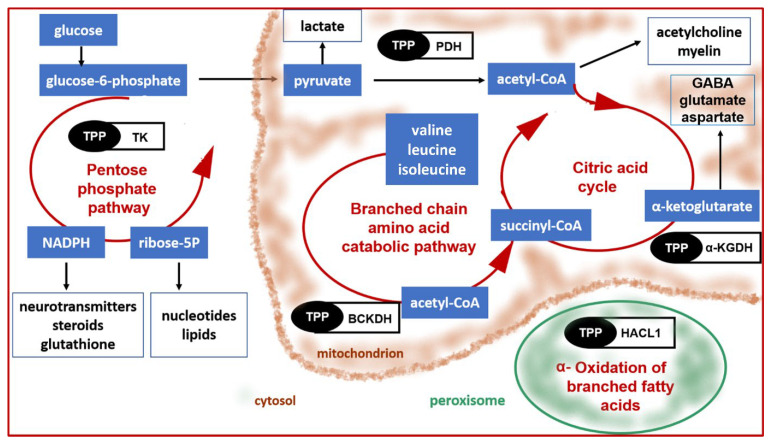
Cellular pathways requiring thiamine pyrophosphate (TPP) as a cofactor. BCKDH, branched-chain α-ketoacid dehydrogenase; PDH, pyruvate dehydrogenase; α-KGDH, α-ketoglutarate dehydrogenase; TK, transketolase; HACL1, 2-hydroxyacyl-CoA lyase 1.

Within the cell, free thiamine is phosphorylated to thiamine pyrophosphate (TPP) by a specific cytosolic kinase, i.e., thiamine pyrophosphokinase (TPK1). The synthesis requires magnesium and adenosine triphosphate (ATP) [201]. TPP is the metabolically active form, which acts as a crucial cofactor in multiple enzymatic reactions in the cytosol (e.g., transketolase), in the mitochondria (see below and Figure 3 and Figure 4) [202,203,204]. The transport of TPP into the mitochondria is mediated via the thiamine pyrophosphate carrier (TPC) encoded by the SLC25A19 gene.

Being a water-soluble vitamin with a half-life of about 10 days, thiamine is not stored in the human body in large quantities. Its body content is approximately 25–30 mg. In conditions of insufficient intake, deficiency can develop over a period of 2–3 weeks.

Excretion of thiamine is mediated mostly by the kidneys, and its rate depends on both glomerular filtration and tubular reabsorption/secretion. The re-uptake from urine to blood in the proximal tubules is enabled mainly by previously mentioned transporters THTR1, THTR2, and OCT1 in the brush border membrane of renal tubular cell and THTR1 and OCT2/3 in the basolateral membrane. Unsurprisingly, the basic transporters THTR1 and THTR2 are up-regulated in thiamine deficiency. If there is an excess of thiamine in the organism, reabsorption switches to active secretion; thus, high thiamine plasma levels are cleared with a high efficacy. In this case, thiamine crosses basolateral membrane of renal tubular cells during transport from blood via THTR1 and OCT2/3 and is transported from tubular cells through the brush border membrane into urine by OCT1 and multidrug and toxin extrusion proteins (MATE1 and MATE2k). Long-term use of diuretics is known to produce thiamine deficiency. Thiamine is also excreted via feces, which is, however, mainly related to thiamine of bacterial origin, but also via sweat and breast milk [2,5,198,205]. Thiamine concentrations are usually in the range of 0.14–0.21 mg/L in breast milk, and levels are dependent on the diet [23].

### 2.4. Physiological Function of Thiamine

Thiamine in its mentioned active form, TPP, serves as a cofactor of several crucial enzymes associated with the metabolism of carbohydrates and branched-chain amino acids. In addition to this role, thiamine is essential for numerous other cellular processes, e.g., the synthesis of nucleic acid precursors, myelin, and neurotransmitters (e.g., acetylcholine), as well as antioxidant defense (Figure 4) [6].

In mitochondria, TPP-dependent enzymes pyruvate dehydrogenase (PDH), α-ketoglutarate dehydrogenase (α-KGDH), and branched chain α-ketoacid dehydrogenase (BCKDH) are involved in pathways that allow production of ATP. PDH catalyzes oxidative decarboxylation of pyruvate to acetyl-CoA within the citric acid cycle (the Krebs cycle), α-KGDH converts α-ketoglutarate to succinyl CoA, which is a key intermediate in the citric acid cycle, and BCKDH catalyzes the oxidation of branched chain amino acids (valine, isoleucine, and leucine) with resulting intermediates acetyl-coenzyme A and succinyl CoA [193,206,207,208].

In the cytosol, TPP-dependent transketolase is involved in the pentose phosphate pathway, that utilizes glucose and produces ribose-5-phosphate and nicotinamide adenine dinucleotide phosphate (NADPH), important for nucleotide, lipid, and neurotransmitter synthesis, as well as antioxidant defense [209]. Another TPP-dependent enzyme 2-hydroxyacyl-CoA lyase 1 (HACL1) is located in the peroxisomes. This enzyme is essential for α-oxidation of branched fatty acids [210].

### 2.5. Thiamine Deficiency

Based on knowledge of the physiological roles of thiamine, deficiency leads to a reduction in oxidative metabolism. Biochemical consequences involve a failure to produce ATP, lactic acidosis due to increased production of lactic acid, and decreased synthesis of neurotransmitters (e.g., acetylcholine, glutamate, aspartate, and GABA). Decreased transketolase activity (TK) impairs synthesis of nucleic acids and glutathione, while insufficient function of α-KGDH leads to defective heme synthesis. Because the central nervous system (CNS) and heart are highly dependent on ATP generated by oxidative decarboxylation, these organs are more sensitive to thiamine deficiency.

Inadequate thiamine intake, impaired absorption or metabolism, and increased demand are the main reasons for vitamin B_1_ deficiency [211]. Lack of thiamine intake can be caused by a vitamin-deficient diet (e.g., historically by polished rice), starvation, or diet rich in carbohydrates. On the other hand, in spite of a sufficient amount of thiamine in the diet, thiamine deficiency may develop by ingesting food containing a high concentration of thiaminases or thiamine antagonists, as reported above. Other factors that decrease absorption of thiamine include aging, malabsorption, and alcohol abuse [15,25,48].

There are also some genetic defects causing impairment of key regulators of the thiamine homeostasis, e.g., thiamine transporters, thiamine pyrophosphokinase (TPK1), and BCKDH. THTR1 dysfunction leads to diabetes mellitus, megaloblastic anemia, and hearing loss, whereas defects of transporter THTR2, mitochondrial TPP transporter, and enzymes TPK1 and BCKDH result predominantly in encephalopathy with severe disability, and early death (Table 2) [26,212,213].

Pregnancy and lactation, fever, hyperthyroidism, and diet with high content of carbohydrates are usually associated with increased consumption of thiamine, while the use of diuretics and diarrhea contribute to thiamine depletion [211]. Serious deficiency of thiamine leads to many syndromes, such as beriberi, predominantly with neurological and cardiovascular symptoms, Wernicke’s encephalopathy, and Korsakoff´s syndrome [214].

Beriberi, also called clinically apparent thiamine deficiency, was described in Chinese literature as early as 2600 B.C. This disease is classified as a dry (nervous) or wet (cardiac) form. The main feature of dry beriberi is peripheral neuropathy leading to paresthesia and paralysis. “Burning feet syndrome” may occur at the beginning of neuropathy. Several characteristic symptoms include a loss of sensation and weakness in legs and arms, muscle pain, etc.

Wet beriberi is predominantly characterized by cardiovascular manifestation, primarily affects the heart, and can lead to cardiac abnormalities characterized by abnormal electrocardiogram (ECG) and, ultimately, to congestive acute heart failure with edema, tachycardia, and an enlarged heart [215,216]. In Japan, acute fulminant hypovitaminosis with cardiopathy accompanied by metabolic acidosis has been reported in chronic alcoholics. This condition is referred to in the literature as wet beriberi “shoshin” [217,218].

In children, thiamine deficiency, known as infantile beriberi, occurs in infants who are breast-fed by thiamine-deficient mothers or with thiamine-deficient diet. The disorder is characterized by rapid onset and by an acute condition involving mainly neurologic and cardiac symptoms, e.g., vomiting, tachypnoea, restlessness, sleeplessness, convulsions, paralysis, aphonia, cardiac dysfunction, and heart failure. Without proper thiamine supplementation, fatality occurs quickly in many cases, notwithstanding the best available supportive treatment. Those children who survive the early stages continue to live with developmental disabilities, e.g., movement and motor skills difficulties, epilepsy, loss of motor function, and language impairment [219,220].

Severe thiamine deficiency can affect the CNS more profoundly. This cerebral beriberi is referred to as Wernicke´s encephalopathy and Korsakoff’s psychosis [221,222]. The diagnosis of Wernicke´s encephalopathy is based on a “triad” of signs, which include impaired eye movement, gait ataxia, and cognitive impairments. If left without treatment, irreversible neurologic damage can cause another clinical manifestation known as Korsakoff’s psychosis. This syndrome involves loss of recent memory, amnesia, and confabulation. The harmful effect of thiamine deficiency on brain function can be explained by strong dependency of the brain on the oxidative metabolism [15,222]. When Wernicke’s encephalopathy accompanies Korsakoff’s psychosis, the combination is called Wernicke-Korsakoff syndrome [223]. This syndrome is one of the most severe neuropsychiatric sequelae of alcohol abuse, although it has been observed in other disorders, e.g., malnutrition or genetic mutations of thiamine transporters. It seems that alcohol impairs intestinal absorption of thiamine. Animal studies suggest that ethanol in the small intestine inhibits the gene expression of thiamine transporter THTR1 but not thiamine transporter THTR2, while, in the large intestine, it inhibits the gene expression of both THTR1 and THTR2. Hence, even if thiamine is present in the intestinal tract, it is not absorbed in the presence of alcohol. Up to 80% of people with chronic alcoholism develop thiamine deficiency because of reduced gastrointestinal absorption. Due to this effect of alcohol on the thiamine absorption, thiamine administration should be intravenous. Moreover, people who abuse alcohol usually have an inadequate intake of essential nutrients, including thiamine [224,225].

Detailed overview of analysis of vitamin B_1_ in biological fluids is summarized, together with other vitamins, in Table 3 and Supplementary Data Table S1.

### 2.6. Pharmacological Use of Thiamine

Thiamine is available as a dietary supplement, and, in cases of severe deficiency, it represents a valuable drug. Multivitamin supplements usually contain about 1.5 mg of thiamine. Recommended Dietary allowances (RDAs) for optimal supply of the organism, which must be taken daily, are 1.1 mg/day and 1.2 mg/day for women and men, respectively [226]. Recommended dietary allowances for thiamine according to age and gender are listed in the Table 4.

In critically ill patients, thiamine supply in the body is depleted within 5–7 days. Therefore, the dosage of vitamin in patients with severe infections, operations, or polytraumas is many times higher, not only due to the increased need in tissues but, mainly, due to increased urinary loss. In these cases, the daily dose for a 70 kg patient is 100–300 mg i.v. [262,263].

For treatment of vitamin B_1_ deficiency, a wide range of therapeutic approaches and doses of thiamine are reported in literature. In the cases of severe deficiency (e.g., beriberi), the World Health Organization recommends daily doses of 50–100 mg i.v. in adults, then 10 mg/day i.m. for about one week, followed by a daily p.o. dose of 3–5 mg for at least six weeks. For infantile thiamine deficiency, the initial intravenous dose is lower, 25–30 mg, followed by the same intramuscular and oral doses as for adults. Patients suffering from early stages of Wernicke-Korsakoff syndrome are treated by subcutaneously or intravenously administered thiamine in a dose of 50–100 mg twice daily. Then, the vitamin should be administered orally for several weeks [22,264]. Thiamine is also taken for conditions related to various genetic disorders of thiamine transport and metabolism, as shown in the Table 2. Disorders of thiamine absorption are circumvented by its parenteral administration. It is also possible to administer the active TPP parenterally [265].

Moreover, thiamine is used to cure nervous disorders (polyneuropathy of various origin) that resemble thiamine avitaminosis with its symptoms. Other uses include treating some patients at risk of thiamine inadequacy, e.g., patients with diabetes, heart failure, HIV infections, and Alzheimer´s disease [3,266,267,268].

**Figure 5:**
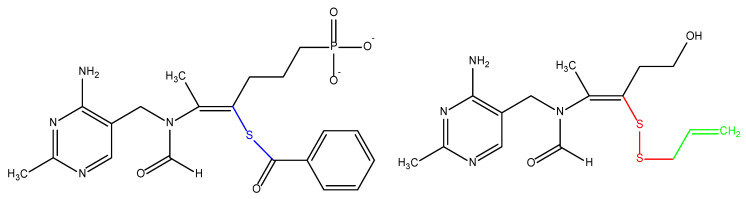
Structure of benfothiamine (**left**) and allithiamine (**right**).

As mentioned, water-soluble thiamine has a limited bioavailability. To overcome this limitation, lipid-soluble derivatives of thiamine were developed. The first lipophilic thiamine analogue was isolated from garlic (*Allium sativum*) extract in 1950s. It was an allyl disulfide derivate known as allithiamine. It is more readily absorbed in the intestine, more stable than thiamine, and is not decomposed by thiaminase [22,134,135,136,137]. Then, based upon the molecular structure of allithiamine, a class of synthetic derivatives have been synthesized, such as sulbutiamine (O-isobutyryl thiamine disulfide), fursultiamine (thiamine tetrahydrofurfuryl disulfide), benfotiamine (S-benzoylthiamine O-monophosphate; see Figure 5), etc. These lipid-soluble derivatives are better absorbed because they can easily diffuse through membranes via passive diffusion, thus circumventing the rate-limiting transport. They have enhanced bioavailability after oral administration compared with an equivalent dose of water-soluble thiamine hydrochloride or mononitrate. Therefore, they are more suitable for therapeutic purposes [135,269].

Benfotiamine, which is increasingly being used in clinical settings, is dephosphorylated in the gut after ingestion to highly lipophilic S-benzoylthiamine, which readily crosses through biological membranes. It is known that it is converted into thiamine in tissues and then into the known thiamine metabolites (thiamine monophosphate and TPP) [270]. Due to its enhanced bioavailability and improved efficacy, benfothiamine is more effective compared to water soluble thiamine in the treatment of subclinical thiamine deficiency and overt thiamine deficiency. It should be mentioned that thiamine hypovitaminosis is more common than thought, primarily due to the discrepancy between high caloric diet and the low intake of vitamins characteristic for contemporary diets [271].

**Figure 6:**
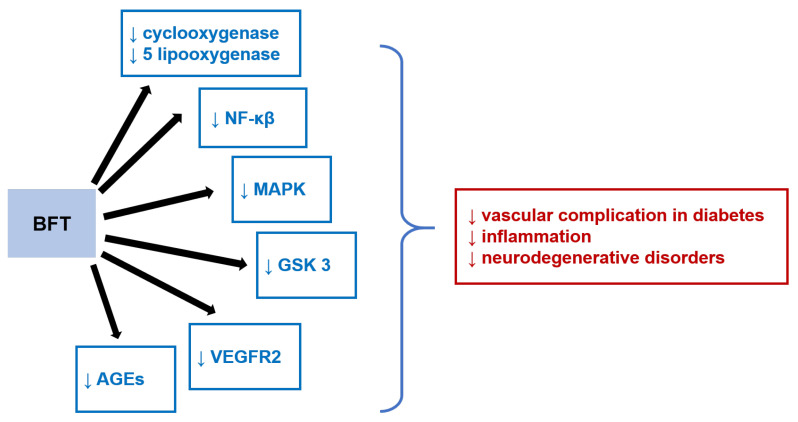
Other effects of benfothiamine. NF-κβ, nuclear transcription factor κB; MAPK, mitogen-activated protein kinases; GSK 3, glycogen synthase kinase-3; VEGFR2, vascular endothelial growth factor receptor 2; AGEs, advanced glycation end-products.

Compared to thiamine, benfothiamine has been shown to influence some signaling pathways, such as arachidonic acid, mitogen-activated protein kinases (MAPK), nuclear transcription factor κB (NF-κβ), glycogen synthase kinase-3 (GSK 3), vascular endothelial growth factor receptor 2 (VEGFR2), and advanced glycation end-products (AGEs); see Figure 6. These signaling pathways are involved in a variety of pathological conditions, so benfothiamine may be beneficial in the treatment of related diseases, e.g., inflammation, neurodegenerative disorders (e.g., Alzheimer´s disease), and diabetes-related vascular complications (neuropathy, retinopathy, nephropathy, and cardiac failure) [272,273].

In the clinical practice, benfothiamine is often used in the combination with other B vitamins (mainly cyanocobalamin/B_12_/ and pyridoxine/B_6_/) in adjuvant pain management (e.g., diabetic polyneuropathy, postherpetic neuralgia, and trigeminal neuralgia). Several clinical studies have shown that the effect of the combination is significantly greater than that seen for individual vitamins. Doses in the studies vary [274].

### 2.7. Toxicity of Thiamine

Thiamine is considered to be safe, with low toxicity. In animal studies, LD_50_ (p.o.) of thiamine hydrochloride in mice was described in a range between 3 and 15 g/kg body weight [275].

In humans, orally ingested thiamine is usually administered for a long period of time without adverse effects, even at doses of several hundred milligrams daily. Thus, orallyingested thiamine has a very low risk of adverse effects. This is related to the fact that oral intakes above 5 mg are associated with lower efficacy of absorption. Moreover, absorbed excess of thiamine is excreted in the urine. In individuals receiving parenteral thiamine supplementation, the most commonly reported adverse effects have been attributed to allergic reactions, which include sensation of heat, urticaria, pruritus, angioedema, diaphoresis, cyanosis, and anaphylaxis [264].

Reports show rare cases of adverse events at doses from 100–300 mg (i.v.) and, more frequently, at higher doses up to 500 mg (i.v.) daily. Allergic persons should not take thiamine supplements to avoid a hypersensitivity reaction. Thiamine is safe to use while breastfeeding [276]. Some parenteral thiamine products may contain aluminium. Therefore, these products should be used with caution in individuals with renal impairments, particularly in premature infants, to avoid the accumulation of aluminium and subsequent aluminium toxicity [277].

### 2.8. Drug-Vitamin Interactions Associated with Thiamine Deficiency

In addition to mentioned diuretics (mainly furosemide) that increase urinary excretion of thiamine and often lead to vitamin B_1_ deficiency [278], there are additional drugs that can affect thiamine kinetics. A cytostatic drug, 5-fluorouracil, inhibits the phosphorylation of thiamine to the active form TPP [279]. Fedratinib, a Janus kinase 2 (JAK2) inhibitor used as an anticancer drug, inhibits crucial intestinal thiamine transporter THTR2 and contributes to the development of Wernicke´s encephalopathy [280]. Metformin also causes drug-vitamin interaction at THTR2 [281].

## 3. Riboflavin—Vitamin B_2_

### 3.1. Introduction and Properties

Riboflavin, vitamin B_2_, was the second vitamin to be isolated from the B-complex. It is also a water-soluble, yellow-orange organic compound consisting of methylated isoalloxazine core and ribityl side chain, which increases its solubility and allows biosynthesis of active cofactors. Riboflavin is relatively stable to heat and atmospheric oxygen, particularly in an acidic environment. It is highly sensitive to light, degraded by reducing agents, and unstable in alkaline solutions [17,51,69,282,283,284,285,286,287,288,289].

While its presence in milk was already observed in 1879, even before thiamine, its isolation and chemical synthesis were successful several decades later in the 1930s [290,291]. Most of its properties and its importance for health have been known for some time; however, novel information has been appearing concerning potential use of this vitamin in metabolic diseases, migraines, and many other indications.

### 3.2. Sources of Riboflavin

As an essential vitamin and a crucial enzymatic cofactor for most organisms, riboflavin is present in a large variety of foods. Plants, fungi, and most bacteria [14,292,293,294,295,296,297,298] are capable of synthetizing riboflavin. On the contrary, humans and animals lack an endogenous biosynthetic pathway, and they must obtain it exogenously from diet [299,300,301]. According to population studies conducted in western countries, milk and other dairy products contributed the most to overall riboflavin uptake, followed by meat, cereals, and vegetables [284,302,303,304,305]. Eggs, legumes, nuts, mushrooms, and organ meat (liver) are also important sources of the vitamin [17,283,306,307,308]. The content of riboflavin in some foodstuffs is shown in Table 5.

Food processing and storage may influence riboflavin content [51]. Primary processing of cereals significantly reduces riboflavin content [313]. The amount of riboflavin in milled refined wheat flour decreases by 38–73% in comparison to whole wheat flour [56,57,58]. Similarly, riboflavin losses in white rice and various maize milled products are 33–57% and 60–75%, in contrast to brown rice [60,61,109] and whole corn kernels [62,63], respectively.

Riboflavin losses are usually rather low (12–15%) during heat processing [21,51,75,84,86]. The major drop of riboflavin (10–30%) in meat during heat culinary processing (such as boiling, stewing, roasting, and frying) is attributed to leaching into cooking water or dripping. Consumption of meat including cooking liquids, e.g., soup or gravy, may retain most of the riboflavin [70,73,77,100,108,112,314]. In legumes, boiling reduces riboflavin content by about 25% due to leaching into water, while stewing retains it [70,74]. Boiling of legumes caused greater riboflavin losses when a prior soaking was carried out in alkaline solution (sodium bicarbonate) [113]. The riboflavin amount in rice is reduced due to washing before cooking and boiling in excess water. These loses are 11–26% and 25–35%, respectively [82,110]. Analogously, boiling, stewing, frying, and baking of mushrooms lead to riboflavin losses of 5% in the total dish (including cooking liquid, sauce, and soup); in the case of boiling with discarding of the water, an additional reduction of riboflavin amount by 30% takes place [70]. Similar circumstances appear with vegetable riboflavin; steaming is preferred to boiling as riboflavin declines by 5% during the former type of processing, while by 30% during the latter one [70,72,73,111]. Riboflavin remains practically unaffected by heating of milk and baking of cheese [71,72]. Pasteurization or ultra-heat treatment of milk results in riboflavin loss of about 2%, whereas a time-dependent decline in the vitamin levels during milk boiling has been noticed [116,315,316]. Extrusion processing of oat whole grains and maize grits lead to no loss and 14% decrease of riboflavin, respectively [119]. Losses of riboflavin during baking of wheat bread and fortified cookies are 10% and 2–24% [76,115]. Boiling, poaching, and frying of eggs lower riboflavin content by 6%, 18%, and 8%, respectively [107]. The canning process may result in a small decrease of riboflavin content in vegetables and mushrooms, as well as pork luncheon meat [125,126].

The amount of riboflavin in mushrooms significantly decreased during frozen storage [81,105,128], while that in freeze-dried meals declined only by 3% during 24-month storage at 1 °C, 30 °C, or 40 °C [129]. Losses of riboflavin in frozen vegetables due to blanching as a pre-freezing treatment have been reported [126]. Riboflavin is relatively stable to ionizing radiation, which has been used as a sterilization method for foods [51,68,121,122,123,124,288].

The most important factor influencing the stability of riboflavin is light in the range of 350–560 nm, with the greatest effect caused by light in the range of 400–520 nm, particularly in a liquid medium [51,286,287,288]. Fluorescent light is less harmful than direct sunlight, but products in transparent packaging can be affected by strip lighting in retail outlets [51]. Although riboflavin is stable to the heat processing of milk, one of the main causes of loss in milk and dairy products is from exposure to light. Liquid milk exposed to light can lose 20–80% of its riboflavin content within 2 h, with the rate and extent of loss being dependent upon the light intensity, the temperature, and the surface area of the container exposed [51,117,287,315]. Riboflavin upon light exposure is cleaved, and it also forms highly reactive oxygen species, which play a part in photosensitized reactions with other compounds [284,285,288]. In this way, riboflavin photosensitization leads also to significant losses of other vitamins, including folate, thiamine, ascorbate (vitamin C), and vitamins A, D, and E, and becomes critical for food sensitivity to light exposure resulting, among others, in oxidative damage of proteins and lipid oxidation (e.g., degradation of unsaturated lipids and formation of toxic cholesterol oxides) [285,286,288,317]. Therefore, it is important to keep riboflavin food sources in dark environments (suitable containers or dark conditions) [306]. Alternatively, a decrease in the degradation rate of riboflavin in milk exposed to light was achieved by ultra-high pressure homogenization due to particle size reduction and, consequently, a decline of milk transparency owing to scattering and absorbance of the wavelengths related to photodestruction of the vitamin [318]. Riboflavin photosensitized decomposition of unsaturated lipids and proteins is responsible for development of light-induced strong off-flavor in milk and dairy products [284,285,317,319]. Similar photooxidation takes place in cheese, butter, and drinking yogurts, but the solid or semi-solid structure limits oxidation damage to the surface [286]. Riboflavin in stored human milk used for infant feeding is highly susceptible to photodegradation upon exposure to sunlight. This decomposition may result in a decrease in riboflavin under the recommended intake range for infants 0–6 months of age. When stored in a refrigeration, however, it is stable for as long as two weeks [320,321,322]. For this reason, banked human milk should be protected from light during storage and processing [322]. Beer is light-sensitive, thanks to riboflavin content—exposure to light develops an off-flavor known as “skunky”, which is derived from photodecomposition of bitter hop acids (isohumulones) sensitized by riboflavin [286]. A proper packaging, such as brown bottles which protect against fluorescent light or sunlight, is needed [285,286]. Riboflavin content in an enriched pasta product decreases gradually under daylight (losses of 78%), whereas it does not change significantly in the dark (losses 0.8%) during eight months of storage at ambient conditions [130]. Riboflavin remains stable in white bread wrapped in transparent packaging and kept in a lit retail area [51]. Contrarily, riboflavin level increases up to four times in germinated edible seeds and cereals, depending on the species [90,94,95,96,132,133,323].

Riboflavin for use in both human and animal nutrition has been industrially manufactured since 1950s by chemical synthesis from ribose (which is easily obtained from glucose), 3,4-dimethylaniline, and barbituric acid [282,324,325]. Fermentation processes employing some microbial strains that produce riboflavin in amounts exceeding their own metabolic requirements were implemented at industrial scale in the 1990s [282,295]. Around 2000, after using both the traditional chemical and the biotechnological process for several years in parallel, the former was replaced by the latter, which became economically and ecologically more feasible (costs reduced by 43% in relation to the former) [282,299,301,326,327]. Today, commercial riboflavin production is exclusively accomplished by microbial fermentation with major microbes being ascomycete *Ashbya gossypii* and bacterium *Bacillus subtilis* [299,300,326,328,329,330,331,332,333,334,335,336].

Fortified foods serve as an additional dietary source of riboflavin in many countries [17,92,173,174,178,179,181,182,305,337]. The most common foods that are fortified with riboflavin are cereals, including those ready to eat, wheat and maize flour, bread, dairy products, and baby food [11,174,282,307,317]. No extensive research has been made to biofortify crops for riboflavin [61,92]. However, lactic acid bacteria are being investigated to improve riboflavin concentrations in fermented foods. Lactic acid bacteria are used worldwide as starter cultures in the food industry for production of a large variety of fermented foods, such as yogurt, cheese, fermented sausages, and pickles. Some strains of lactic acid bacteria are able to synthetize riboflavin, which represents a practical advantage relying on food fortification in situ. Considering the extensive application of lactic acid bacteria in the food industry, those capable of producing riboflavin have potential for the development of diary- and cereal-based functional foods with increased levels of riboflavin. Such foods could help consumers meet their daily recommended intakes and could be useful in preventing clinical and subclinical riboflavin deficiencies [300,326,338,339,340,341,342,343,344,345].

### 3.3. Pharmacokinetics of Riboflavin

#### 3.3.1. Absorption

Dietary riboflavin is generally present either bound to proteins or in the form of cofactors—flavin mononucleotide (FMN) and flavin adenine dinucleotide (FAD). After protein denaturation by digestive enzymes, FMN/FAD is released from flavoproteins by intestinal alkaline phosphatases, and, subsequently, riboflavin is freed from FMN/FAD by pyrophosphatases located in the brush border membrane of the intestinal epithelium [346]. Human gut microbiota also produce the vitamin. Although the human microbiota are not able to supply the full daily need of this vitamin, they are able to compensate likely for a low dietary intake to some extent, as shown in a riboflavin-deficient rat model [342,347]. Riboflavin produced by bacteria in the colon is, to a large extent, present in the absorbable form [348,349].

**Figure 7:**
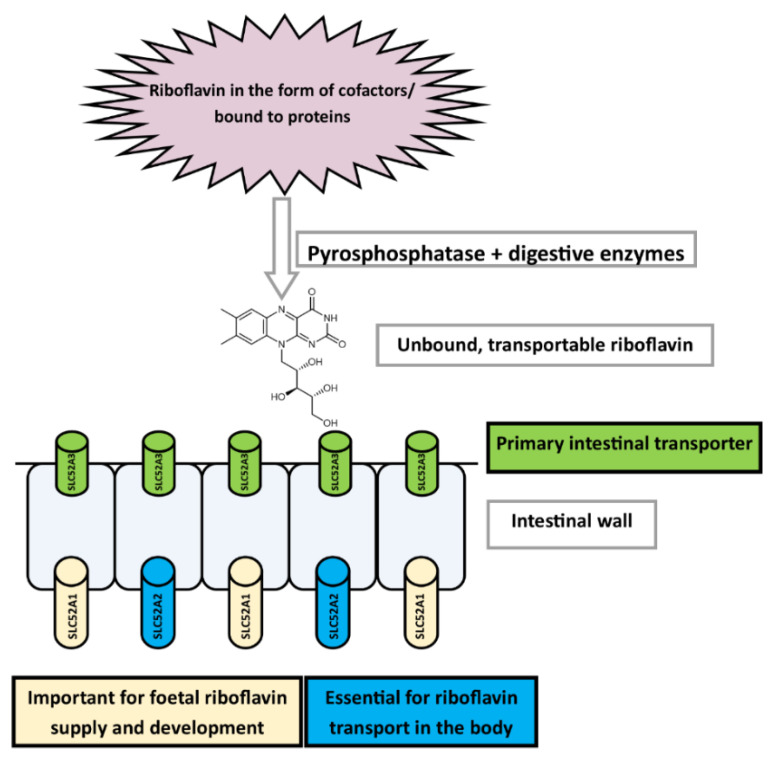
Riboflavin absorption. The majority of riboflavin uptake in the intestine is enabled by SLC52A3, while the other 2 transporters are mainly responsible for basolateral transport to the blood and transport in other cells.

Free and released riboflavin is transported by specific transporters located in the intestinal cells. Some of these transporters have recently been cloned and characterized as members of the Solute Carrier Family 52 (SLC52 or Riboflavin Transporter Family): *hRFVT1* (SLC52A1), *hRFVT2* (SLC52A2), and *hRFVT3* (SLC52A3) [350,351,352]. This carrier-mediated transport is specific and inhibited by structural analogues and metabolic inhibitors, such as lumiflavin, FMN, and FAD. SLC52A2 was also inhibited by amiloride, in addition to riboflavin analogues [353]. The localization of these transporters at the cellular and tissue level is dissimilar (Table 6). The uptake of riboflavin by intestinal cells at the luminal side is mediated mostly by SLC52A3, while its transport through the basolateral membrane to the blood is facilitated by another member of the same family, SLC52A2 (Figure 7). SLC52A2 and SLC52A3 are crucial for riboflavin absorption and transport and have been linked to metabolic diseases, such as Brown Vialetto-Von Laere syndrome (BVVLS). SLC52A1 is not able to compensate for defects of other members of this transporter family, most likely due to differences in their tissue and cellular distribution (Table 6) [350,352,354,355].

#### 3.3.2. Metabolism

##### Uptake and Transport

After cellular uptake, riboflavin is transformed into its biologically active forms by 2 enzymes: riboflavin kinase and FAD synthase (see next paragraph). The reverse process is also possible, but the intracellular hydrolase responsible for release of riboflavin from FMN/FAD is not known. Following absorption, most flavins are localized in blood cells, particularly in erythrocytes, which contain merely a trace amount of free riboflavin but a significant amount of FMN and FAD [356]. Circulating plasma riboflavin is also bound to albumin and immunoglobulins, but this amount is significantly lower than its cellular levels. Comparison of plasma and erythrocyte concentrations of these compounds can be seen in Table 7.

###### Synthesis of Flavin Cofactors

**Figure 8:**
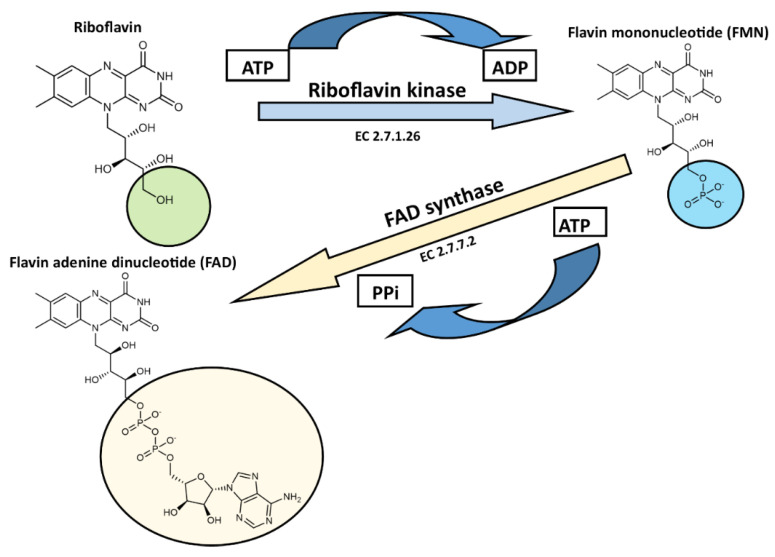
Cofactor biosynthesis. After penetration to the cells by facilitated transport mechanism, riboflavin is transformed into its biologically active forms by 2 cytosolic enzymes, riboflavin kinase and FAD synthase. ATP—adenosine triphosphate, ADP—adenosine diphosphate, PPi—pyrophosphate. See text for detailed description.

Following riboflavin uptake into the cell, it is quickly transformed into its active forms that serve as enzyme cofactors. Two enzymes are necessary for flavin cofactor synthesis: Riboflavin kinase (ATP: riboflavin 5′ phosphotransferase, EC 2.7.1.26), which transfers a phosphoryl group from ATP to riboflavin to form FMN; and FAD synthase (ATP: FMN adenylyltransferase, EC 2.7.7.2), that adenylates FMN to FAD (Figure 8). In bacteria, both these steps are catalyzed by a bifunctional enzyme, FAD synthetase [357,358,359]. Because FAD forming enzyme in humans and other eukaryota is unrelated to the bacterial FAD synthetase, it is a prospective target for future antimicrobial drug development, with the potential of developing highly potent and highly selective antibacterial drugs [360].

###### Excretion

Riboflavin, being a water-soluble vitamin, is primarily excreted in urine. The majority of riboflavin in urine is present in the form of unchanged riboflavin; however, several metabolites have been found, as well. These include 7α- and 8α-hydroxyriboflavins. 8α-sulfonyl flavin has also been found, albeit in a smaller amount. 10-hydroxyethylflavin, lumiflavin, and other lumichrome compounds are products of intestinal degradation of riboflavin, therefore being present only in minute concentrations in human urine [355,361].

### 3.4. Physiological Functions of Riboflavin

#### 3.4.1. Flavoproteins

Both bioactive forms of riboflavin, FMN and FAD, possess an isoalloxazine ring that is able to reversibly donate or accept 2 electrons. The majority of human enzymes utilizing these cofactors, therefore, catalyze reduction-oxidation reactions. Analysis of human flavoproteins has shown that all these enzymes are oxidoreductases, except for one transferase and one lyase (Supplementary Data Table S2) [362]. A complex analysis of a database of flavoproteins from different species yielded similar results, with 91% of the enzymes being oxidoreductases [363]. A higher number of enzymes is utilizing FAD as a cofactor compared to FMN. Indeed, only 16% of human flavoenzymes use FMN as a cofactor. Most of the flavoenzymes (90%) bind its cofactor non-covalently and, in the case of FAD, primarily utilize the Rossman fold of the protein structure [362,363].

#### 3.4.2. Flavoproteins’ Importance in Human Health

Flavoproteins are also involved in synthesis of other cofactors, such as coenzyme A, coenzyme Q, heme, pyridoxal 5-phosphate, and various hormones. Disruption of these processes has severe consequences for humans.

Biosynthesis of heme takes place both in cytosol and in mitochondria. One of the enzymes present in mitochondria, protoporphyrinogen IX oxidase (PPOX, EC 1.3.3.4), is FAD-dependent, and diminished activity of this enzyme results in genetic defect known as variegate porphyria. This disease has various clinical forms and times of onset, and it may cause severe and life-threatening acute attacks. Unfortunately, due to the cause of the disorder, only prophylactic and symptomatic treatment is currently available. In addition to avoiding exacerbating factors, such as certain drugs or sunlight, recurring premenstrual attacks can be prevented with gonadotropin-releasing hormone analogues. During acute manifestations, narcotic analgesics are generally used in combination with setrons for nausea and vomiting. Mild attacks should be treated with hemin, an oxidized form of protoporphyrin IX. Cutaneous exacerbations require analgesics for painful lesions and antibiotics for infections. Topical steroids have little to no effect [362,364].

Formation of pyridoxine 5-phosphate, the active form of vitamin B_6_, is also dependent on a flavoenzyme, pyridoxine 5-phosphate oxidase. Several cases of genetic defects of this enzyme were reported with a very poor prognosis and limited treatment options. Most patients developed neonatal epileptic encephalopathy and suffered from mental retardation later on [362,365]. Similarly, formation of coenzyme A from vitamin B_5_ is also dependent on a flavoprotein using FMN, phosphopantothenoylcysteine decarboxylase (EC 4.1.1.36). No defects of this particular enzyme have been recorded, but mutations of other enzymes involved in this pathway have been described with severe manifestation [362,366,367].

Coenzyme Q_10_ is essential for proper function of respiratory chain reactions and electron transport. It is synthesized de novo, and this biosynthesis is dependent on a flavoenzyme, monooxygenase, encoded by coq6 gene. Deficiency of this enzyme was found to be a cause of Q_10_ deficiency, causing steroid-resistant nephrotic syndrome and sensorineural deafness in most patients. In these patients, coenzyme Q_10_ supplementation leads to nephropathy remission and stopped progression of neurological symptoms [362,368,369,370].

Flavoproteins also play an important role in both thyroid hormone biosynthesis and recycling of iodine. Hydrogen peroxide required for T_3_ and T_4_ synthesis is provided by 2 FAD-dependent oxidases, and dehalogenation of inactive iodotyrosine is carried out by FMN-dependent dehalogenase. Several allelic variants of this dehalogenase causing insufficient activity led to hypothyroidism [362,371,372,373].

### 3.5. Riboflavin Deficiency

**Figure 9:**
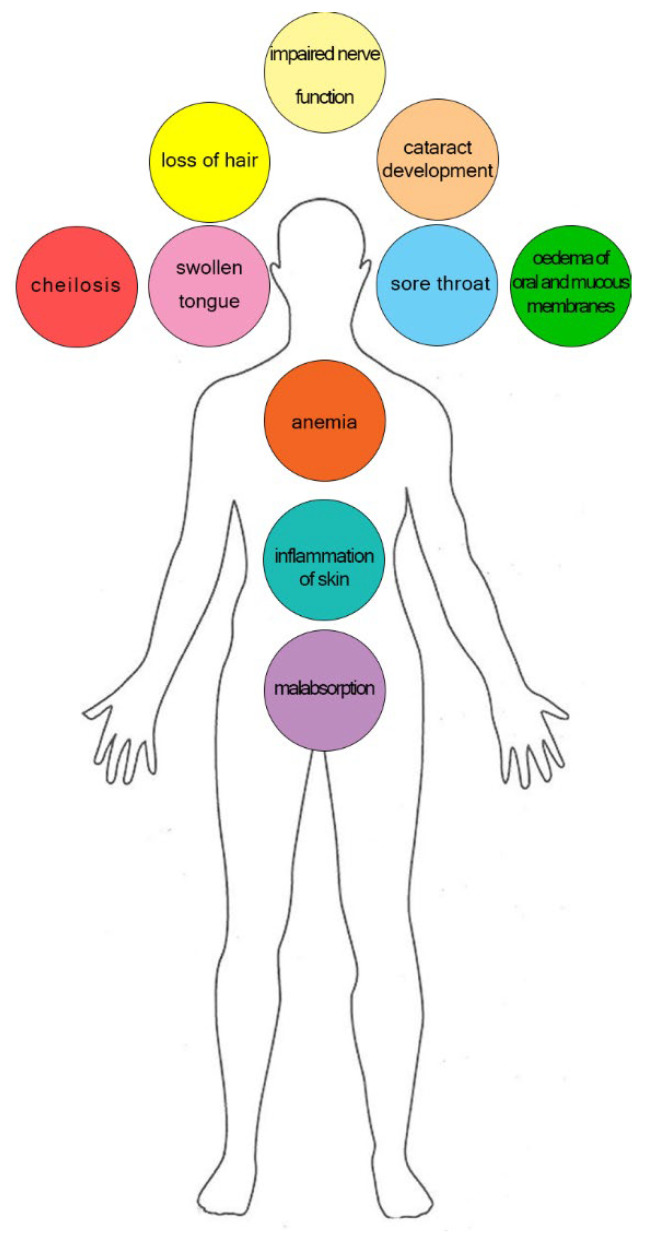
The most common symptoms of riboflavin deficiency.

Insufficient dietary intake manifests clinically after several months of deficiency. Symptoms include sore throat, hyperaemia, edema of oral and mucous membranes, cheilosis, and may progress to loss of hair, inflammation of skin, anemia, swollen tongue, cataract development, malabsorption, and impaired nerve function (Figure 9) [307,374,375].

The importance of sufficient riboflavin supply, and implications of its deficiency, were observed a long time ago as impairment of mitochondrial function and metabolism in mice fed on a riboflavin-poor diet [376]. Because of flavoproteome being involved in many aforementioned biological processes, lack of riboflavin or mutations of these genes has resulted in a plethora of metabolic disorders with a wide range of phenotypes and clinical manifestations.

### 3.6. Analytical Determination

Methods for analytical determination of riboflavin levels are summarized in Table 3 and Supplementary Data Table S1. Due to a small circadian variance in riboflavin levels, rapid metabolism, and fairly short half-life, also different, more biologically reliable methods are currently employed for riboflavin content determination. These methods are based on the estimation of enzymatic activity of flavin-dependent enzymes, such as erythrocyte glutathione reductase activation coefficient or pyridoxamine phosphate oxidase activity. The former is a simple and reliable method; however, it is not suitable for subjects with glucose-6-phosphate dehydrogenase (G6PD) deficiency. In these individuals, a more sensitive and reliable method, pyridoxamine phosphate oxidase activity, should be used, as it is reliable also in individuals with G6PD deficiency [356,377].

### 3.7. Pharmacological Use of Riboflavin

Riboflavin supplementation is a viable and established therapeutical option in several inborn errors of metabolism, either by compensating for transport or synthesis deficiency of flavin cofactors, or by improving the folding or stability of flavoproteins.

Multiple acyl-CoA dehydrogenase deficiency (MADD) is a recessive disorder of metabolism of fatty acids, amino acids, and choline caused by deficiency of electron transfer chain. Severe MADD patients harbor mainly homozygous or heterozygous loss of function variants, while mild MADD patients carry variants affecting protein folding and stability. Severe MADD has a very early onset, often neonatal, multisystem involvement, and patients generally do not survive for more than a year from the time of the onset. In contrast, mild MADD has a later onset and presents with milder clinical symptoms, such as muscle weakness, hypoglycemia, encephalopathy, and rare cardiomyopathies. Due to different causes of these 2 variants and varying clinical outcomes, it is not surprising that riboflavin supplementation has shown excellent results in mild MADD phenotype, while having very low or no effect in severe MADD. Late onset patients with a myopathic phenotype were responsive to riboflavin supplementation in 98% of cases [378,379].

Beneficial effect of riboflavin has also been observed in several patients with acyl-CoA dehydrogenase 9 deficiency. Taking into account that all patients that responded to the therapy harbored missense variants of the gene, the mechanism is most likely similar to mild MADD patients. Riboflavin has been evaluated for a few more inborn errors of metabolism involving flavoproteins, but more clinical trials are required to reach a definitive verdict on the efficacy of riboflavin supplementation. For more information on this topic, see a review by Mosegaard et al. [307].

Deficiency of riboflavin transporters is associated with genetic neuronopathies, known as Brown-Vialetto-Von Laere syndrome and Fazio Londe disease [379,380]. These defects can be efficiently treated with high dose riboflavin supplementation. Early diagnosis is paramount in preventing any serious damage occurring and stabilization of the patients. Symptoms of these mutations have been thoroughly described and summarized in a review by O’Callaghan et al. Most of the patients suffered from impaired hearing (87%), muscle weakness (83%), and neuropathy (89%) [380]. A few patients with mutations of mitochondrial FAD transporter (gene SLC25A32) have also recently been described. This defect is also responsive to riboflavin supplementation but has a different clinical manifestation than MADD [379,381,382].

Apart from potential use of riboflavin for diseases caused by impaired riboflavin transport or utilization, several clinical trials have shown that riboflavin is a viable choice for treatment of migraines. While the exact cause of migraines and their detailed molecular course is unknown, various studies have shown a link between dysfunctional mitochondria and the incidence of migraine headaches with specific mitochondrial haplotypes more prone to developing this type of headaches. Indeed, riboflavin treatment was significantly better in patients with certain genotypes. While the efficacy was not exceeding the conventionally used preventive drugs, such as antiepileptics and propranolol, the risk of adverse effects was significantly lower in riboflavin groups. Riboflavin-treated patients tolerated therapy very well, and the incidence of adverse effects was comparable to placebo treated groups. While more research in terms of pharmacokinetics and genetic component in using riboflavin for migraine prophylaxis is required, it is an interesting prospective use of this vitamin [383,384].

Vitamin B_2_ has also been tested in several other possible indications. While it did not reach the efficacy of currently used drugs, it is a viable adjuvant with positive effects being described with riboflavin intake simply from dietary sources or low dose supplementation. Its described anti-oxidative properties have proven to be beneficial in ischemia—reperfusion oxidative injury. These properties have been reported using various models, such as SH-SY5Y neuroblast cells, rabbit myocardium, and lung and brain contusion injury of rats [385,386,387,388].

Riboflavin has also shown some positive adjuvant effects when used in combination therapy with clinically used drugs. In combination with antibiotics, riboflavin improved the survival rate; however, only animal studies are available so far [389]. In addition to having an effect on *Plasmodium falciparum* in vitro on its own, riboflavin reduced anemia and improved the overall outcome of the infection when combined with chloroquine in malaria therapy [390,391]. Riboflavin is also an important part of immune response, promotes proliferation of neutrophils and monocytes and their phagocytic activity, and is particularly beneficial in reducing inflammatory reaction and nociception [389,392,393,394]. Photosensitizing properties of riboflavin may be used in combination with long wave length ultraviolet radiation to inactive pathogens, such as HIV, pseudorabies virus, West Nile virus, parvovirus, *Escherichiacoli*, and *Leishmania* spp. [389]. Cataract formation is associated with increased age, and riboflavin had some preventive effect on age-related cataract. In addition, the majority of cataract patients had a shortage of riboflavin, proving the importance of this vitamin in preventing cataract formation [395,396].

A significant reduction of risk (35%) in developing premenstrual syndrome has been observed in women with increased intake of riboflavin from dietary sources [397]. Some studies have shown decreased incidence of breast, lung, ovarian cancer, and colorectal carcinoma correlated with higher riboflavin intake and reduction of metastasis of melanoma in an animal model. Riboflavin deficiency, on the other hand, has been linked to higher susceptibility to cancer, such as esophageal cancer [389].

Riboflavin also improves type 2 diabetes mellitus, mainly through its role in glutathione recovery, but a decrease of blood sugar and increased expression of GLUT-4 transporter has also been observed in mice [398].

### 3.8. Toxicity of Riboflavin

So far, partly due to the saturable transport of riboflavin at the GIT level, there are no reported instances of riboflavin overdose or toxicity (even when high doses of the vitamin were administered), apart from minor non-specific symptoms of gastrointestinal nature, such as diarrhea or vomiting [284,383,399,400].

### 3.9. Drug Interactions Affecting Pharmacokinetics and Interfering with Physiological Function of Riboflavin

#### 3.9.1. Boric Acid

This compound is used locally as an antiseptic, but, in cases of intoxications, such as in oral ingestion, a clear depletion of riboflavin has been observed. This pharmacokinetic interaction is based on the fact that boric acid easily reacts with polyhydroxyl compounds. Hence, a complex of riboflavin-borate is formed. This complex increases water solubility of riboflavin and, therefore, leads to increased urinary excretion of riboflavin with subsequent depletion of riboflavin stores and hypovitaminosis [401,402].

#### 3.9.2. Doxorubicin

This antineoplastic compound causes significant riboflavin deficiency, which may contribute to its side effects [403,404]. The reason is inhibition of flavin cofactor synthesis.

#### 3.9.3. Antipsychotics

Some older antipsychotic drugs from the phenothiazine class share structural similarities with flavin co-factors and, therefore, are able to competitively inhibit flavin-dependent enzymes. Chlorpromazine, in particular, was able to inhibit flavin-dependent enzymes, such as D-amino acid oxidase. It also blocked incorporation of riboflavin into FAD in vivo. This biosynthetic pathway is stimulated by thyroxine, which was shown to increase flavin biosynthesis in a rat model. Interference of this pathway caused by chlorpromazine therapy resulted in a state similar to that observed in hypothyroidism. In addition to lower utilization of riboflavin, the urinary excretion of riboflavin was also significantly increased in chlorpromazine-treated animals using therapeutical doses of the drug [405,406,407,408,409,410]. This effect was most pronounced in the heart, using a rat model [411].

#### 3.9.4. Antidepressants

Tricyclic antidepressants share, as well, structural similarities with flavin compounds and are able to interact with flavin metabolism, utilization, and excretion, albeit their effect is not as significant as is the case of chlorpromazine. The mechanism of this interaction is, however, very likely the same [401,407,411].

## 4. Niacin—Vitamin B_3_

### 4.1. Introduction and Properties

Niacin, also known as vitamin B_3_, is the third member of water-soluble B vitamins. The term niacin denotes nicotinic acid (NA, pyridine-3-carboxylic acid) but is often used to name a group of related chemicals, mainly nicotinamide (pyridine-3-carboxamide) and related derivatives, such as nicotinamide riboside [412,413,414]. Most dietary niacin is in the form of NA and nicotinamide, but some foods contain small amounts of nicotinamide adenine dinucleotide (NAD) and nicotinamide adenine dinucleotide phosphate (NADP).

Niacin, in both forms (NA and nicotinamide), is the most stable water-soluble vitamin [74], and it is normally highly resistant to atmospheric oxygen, acids, heat, and light in both aqueous and solid systems [69,75,131,415,416,417,418].

### 4.2. Sources of Niacin

Plants, most fungi, and bacteria synthesize niacin [14,419,420,421,422,423,424]. Most mammals (except cats; for instance [425]), including humans, can convert the indispensable amino acid tryptophan [426], partially, to nicotinamide, mainly in the liver, so tryptophan is considered a dietary source of niacin, too [427,428,429,430]. In this regard, niacin is unique among the water-soluble vitamins [418,430,431]. Conversion varies among people; approximately 60 mg of tryptophan is equivalent to 1 mg of niacin [92,432,433,434]. However, there is a preferential use of tryptophan for protein synthesis, before it becomes available for production of niacin [417,429]. Under common conditions, only about 2% of dietary tryptophan is converted to niacin [428,429,435]. The conversion is also affected by many factors, such as, among others, intake of adequate amounts of high quality proteins, and vitamins B_2_ and B_6_ [427,430,436,437,438,439], but not by exogenous nicotinamide itself [440]. The conversion cannot cover the needs for niacin, which must be, therefore, supplied from diet, but tryptophan, apparently, significantly contributes, as dietary niacin provides about 50% of the needed daily vitamin B_3_ [417,441,442,443]. Main dietary sources of niacin include meat, whole grains, and milk and dairy products [18,303,432,444]. Peanuts, fish, mushrooms, and yeasts are rich in niacin [308,417,435,444,445,446,447,448]. Legumes and nuts are also useful foodstuffs providing niacin [441,449]. Coffee consumption on a regular basis notably contributes to niacin intake in humans, as well [450,451,452,453,454]. The contents of niacin in some selected foodstuffs are shown in Table 8. Foods containing high amounts of proteins with tryptophan, such as milk, cheese, and eggs, are also suitable sources of niacin [435,441,444].

There are little data on interference of different food products on absorption or utilization of niacin. Tea does not affect bioavailability of niacin [33]. It has been suggested that dietary excess of leucine, for example, in populations whose staple are cereals rich in leucin, such as sorghum or corn, may antagonize niacin synthesis from tryptophan [416,428,455,456,457,458,459]. However, the pellagragenic effect of leucine remains controversial [93,416,460,461,462,463].

Food processing may influence content of niacin [415]. With respect to cereals, their primary processing consisting of milling and the associated removal of the germ and the external layers of the grain, where important micronutrient are mostly located, results also in significant losses of niacin [99,313,416,464,465]. Niacin content in refined wheat and rye flour decreases by 75–82% and 51% in comparison to whole wheat and rye flour, respectively [56,313]. Likewise, niacin is reduced in cornmeal by 47% compared to whole grain corn flour [63]. Niacin losses, in comparison to brown rice, are 64–79% and 38% in non-parboiled and parboiled white (i.e., milled) rice, respectively. The reason is similar as in the case of thiamine: a part of niacin diffuses from the vitamin-rich outer bran layer into the endosperm during parboiling and, thus, is retained during the following milling [109,140,142].

Niacin is stable during heat processing of meat, vegetables, and legumes; leaching into the cooking water or drippings is usually the main cause of its loss, which can make about 5–55% [70,72,73,74,75,78,97,108,111,113,415,473,474,475,476]. For example, niacin content in boiled beef is 45% in comparison with that in raw meat, and the rest, 55%, occurs in the soup [100]. If the cooking water is not discarded, the overall losses of niacin remain minimal [416,444]. Niacin losses are 10–25% during blanching due to washing out by water. A high temperature and a short water-blanch time gives better retention than a low temperature and long blanching time; steam blanching is superior to water blanching [415]. Niacin amount in rice is reduced due to washing before cooking (rinsing) and boiling in excess water that is discarded by 3–13% and 25–50%, respectively [109,110]. No losses of niacin occur during boiling or simmering of rice when the cooking water is kept [72]. Rinsing had almost no effect on niacin in brown rice but decreases niacin levels in enriched parboiled and non-parboiled white rice (considerably in the latter). Cooking in variable amounts of water lowers niacin contents with increasing water amounts. The loss is less expressed in brown rice but to the greatest extent in non-parboiled white rice [163]. Niacin is stable during baking of bread [415]. Losses of niacin during baking of fortified cookies were about 1–12% in dependence on baking temperature and time, while, for comparison, those of riboflavin and thiamine were 2–24% and 2–50%, respectively, under the same conditions [76]. Niacin is also stable during heat processing of milk, cheese [72,472], and eggs [72,107], as well as during canning of vegetables and pork luncheon meat [125,126].

Post-mortem aging of beef can result in up to a 30% loss of niacin over seven days, although the remaining niacin is relatively stable during cooking [415]. Effects of extrusion techniques on retention of niacin in cereal grains (oats and maize) were also investigated; no niacin losses and reduction by 10–25% in oats and maize, respectively, were observed [119]. Studies on dehydration of blanched vegetables showed that dehydration process can give rise to additional losses of niacin, e.g., 5–15%, in cabbage [415]. Niacin, as with riboflavin, is relatively stable to ionizing radiation used as a sterilization technique for foods [68,122,124,415,477]. Niacin content in an enriched pasta product decreased gradually only to a small extent (reduction of 5.4–6.4%) during eight months of storage, independently of the storage conditions (in the dark and under daylight at ambient conditions, and in a refrigerator) [130]. Niacin amounts in button mushrooms declined during frozen storage for 12 months [105].

In cereals, niacin is mostly present as esterified forms unavailable for absorption (also after cooking), namely niacytin consisting of nicotinic acid esterified to polysaccharides, and niacinogenes in which nicotinic acid is esterified to polypeptides and glycopeptides. Only a small part (about 25–30%) of these bound forms is bioavailable and may be hydrolyzed by gastric acid [424,442,444,465,478,479,480,481]. The bioavailability of esterified bound forms of niacin can be substantially improved by treatment of the food with alkali to hydrolyze ester bonds [22,415,442,444,478,482,483,484,485,486]. Nixtamalization is thermo-alkaline processing of maize kernels, which improves technological, nutritional, and sensory characteristics of maize, as well as significantly reduces mycotoxins. The process involves boiling of corn grains in a calcium hydroxide solution, followed by steeping to obtain nixtamal (the steeped maize), which is thoroughly washed and ground to make masa (a wet dough or dried into a flour); a variety of products are obtained from it, and tortilla is the most popular one [63,487,488,489,490,491,492,493,494,495]. Nixtamalization brings about losses in some B vitamins, including niacin by 31–32% [22,488,496]. However, niacin becomes more available in alkali-processed maize than in raw ones, as described above. It was found that nixtamalization effectively doubled the amount of available niacin in corn [497,498]. The process developed by the Mayans and Aztecs, and today used in other countries, such as the United States, effectively releases the bound niacin and appears to be responsible, at least in part, for protection against pellagra in populations of Mesoamerica, where the disease has a very low rate of occurrence [62,416,418,458,483,484,489,492,494,499]. Some researchers hypothesized that changes in amino acid balance, rather than in bound niacin, were responsible for the differences between raw and lime-processed maize in biological activity and pellagragenic action, e.g., increases in isoleucine/leucine ratio [458,487,495,500]; regardless, beneficial anti-pellagragenic effects of alkali treated maize have been demonstrated.

Niacin can be released from its precursors by heating [416,501]. During roasting of green coffee beans, NA is formed from the alkaloid trigonelline (the betaine N-methyl nicotinic acid) by demethylation [416,435,502]. The content of nicotinic acid in roasted beans increases approximately 10- to 25-fold, depending on the roasting time and temperature (i.e., degree of roasting) [416,503,504,505]. The degree of roasting, as well as coffee cultivar and coffee brewing technique, influences the amount of NA in a cup of coffee [416,452,502,504,506]; on average, one cup may cover about 9% of recommended daily intake of NA; thus, coffee consumption could constitute a noticeable part of the niacin daily supply [452,502]. Roasting increased niacin content in peanuts (by 11–33%) [507]. Toasting of wheat bread induced an increase of niacin by 65% due to heat-induced liberation from bound forms [508].

An increase in niacin content occurs in germinated sorghum (by 35–45%) [94,509], millets (by 22%) [94], lentils (by 9–83%) [96], wheat (by 19%) [132,510], and maize (by 64–142%) [509,511]. Natural fermentation of lentil flour increased the amount of niacin by 24–91% [310]. Content of nicotinamide rose tenfold during rye sourdough fermentation due to activity of lactic acid bacteria [512].

Industrial production of vitamin B_3_ is currently based on chemical synthesis and biocatalysis [171,513,514,515,516]. Chemical processes utilize 2-methyl-5-ethylpyridine, 3-methylpyridine (3-picoline), or 3-cyanopyridine as starting materials, which are synthesized from simple compounds, such as acetaldehyde, formaldehyde, paraldehyde, 2-methylglutaronitrile, and ammonia [417,443,517,518]. The chemical routes require high temperature, high pressure, metal catalysts (e.g., oxides of vanadium, titanium, and zirconium), and hazardous chemicals and are usually connected with formation of unwanted waste, including by-products [417,443,515,519]. Biocatalytic processes, on the other hand, make use of enzymes as catalysts, which are operated under mild conditions and are highly efficient in terms of specificity and yields [417]. Those processes are also environmentally friendly and safe [520]. Intensive research has focused on the biocatalytic way for production of vitamin B_3_ [420,519,521,522,523,524,525,526,527,528,529,530,531,532]. Nicotinamide is produced at industrial scale from 3-cyanopyridine by means of immobilized bacterial cells *Rhodococcus rhodochrous* J1, containing an enzyme nitrile hydratase, which catalyzes the hydration of a nitrile to its corresponding amide [417,443,520]; intact whole cells are used as a biocatalyst due to low stability of isolated nitrile hydratase [531]. An industrial fermentation procedure for vitamin B_3_ is still not established; there is little motivation for development a fermentative one with the high-yielding biocatalytic process in place [171,513].

Alongside natural niacin sources, fortified foods provide an additional dietary supply of the vitamin [92,173,175,178,179,183,185,186,416,424,533,534,535]. Fortification of wheat and maize flour, as well as rice, with niacin is in many countries mandatory [92,163,181,182,417,536]. Niacin is also added to bread, breakfast cereals, and pasta [174,416,443,449].

Regarding biofortification, i.e., the augmentation of natural micronutrient levels in crops through breeding and genetic engineering, no efforts have been made to enhance the niacin content of any crop [92]. However, genetic variation of niacin in wheat grain was assessed, but the content of niacin was only poorly heritable; thus, it is unlikely that it could be substantially increased by plant breeding [92,537,538]. On the other hand, different varieties of pigmented rice displayed a wide range of variation in niacin content, which could provide lead strategies for future breeding initiatives [92,539]. The second approach to biofortification, i.e., up-regulation of the niacin synthesis by genetic modification technology, seems to be improbable, at least in the short term, as the biosynthetic pathway of niacin is complex, and its genetic control is not well understood [538,540]. In addition, there are reports suggesting that plants are highly sensitive to changes in the biosynthetic pathway of vitamin B_3_; negative impacts on plant growth and development were observed [172]. Attempts have been made to improve the amino acid profile of maize, since it is naturally poor in tryptophan in contrast to other cereals, such as rice and wheat [62,416]. Doubling tryptophan content in quality protein maize via conventional plant breeding techniques was successful, and some of the tryptophan biofortified varieties have been commercialized [92,541,542,543,544,545,546].

### 4.3. Pharmacokinetics of Niacin

#### 4.3.1. Absorption and Distribution

When NAD and NADP are consumed in foods, they are enzymatically converted to nicotinamide in the gut and then absorbed, together with NA [547]. The primary site of niacin absorption is the small intestine, although some is absorbed in the stomach [412,413,414]. The absorption of nicotinamide is rapid, and it is mediated by Na^+^-dependent facilitated diffusion at low concentrations. When taken in very high doses of 3–4 g, niacin is still almost completely absorbed, but mostly by passive diffusion [548].

Niacin is transported to all tissues, where it is converted into its main active form, the coenzyme NAD. Both forms of niacin enter cells by simple diffusion; however, both nicotinic acid and nicotinamide also enter erythrocytes by facilitated transport to form a circulating reserve pool and support the function of these cells [549].

#### 4.3.2. Metabolism

##### NAD Synthesis

**Figure 10:**
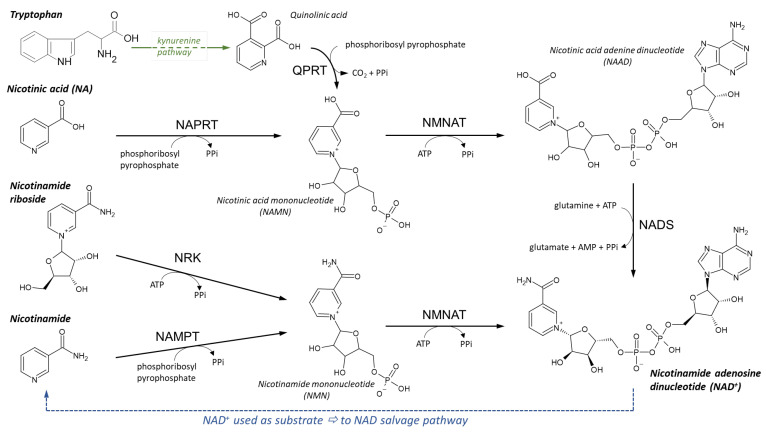
NAD synthesis pathways in mammals. Biochemical reactions involved in production of NAD from dietary sources. The blue arrow indicates activity of NAD^+^-consuming enzymes and entry of thus released nicotinamide into the salvage pathway. See text for detailed description.

The biochemically active form NAD can be synthesized in mammals from all molecules of the vitamin B_3_ group (NA, nicotinamide and nicotinamide riboside) and tryptophan (Figure 10).

NAD is formed from niacin by three metabolic steps in the Preiss-Handler pathway. First, NA is converted to nicotinic acid mononucleotide (NAMN). This reaction is catalyzed by the enzyme nicotinic acid phosphoribosyl transferase (NAPRT) and uses 5-phosphate-α-D-ribose-1-diphosphate as a co-substrate. NAMN is subsequently converted to nicotinic acid adenine dinucleotide (NAAD) by the enzyme nicotinamide mononucleotide adenylyltransferase (NMNAT), using ATP. In the final step, the nicotinic acid part of NAAD is amidated by a transfer of an amino group from glutamine by the enzyme NAD synthase (NADS), consuming another ATP molecule and forming glutamate.

Synthesis of NAD from nicotinamide follows an important salvage pathway. This pathway enables the reutilization of nicotinamide which is released during many biochemical reactions utilizing NAD. In the salvage pathway, the initial step is catalyzed by the enzyme nicotinamide phosphoribosyl transferase (NAMPT). This change of nicotinamide to nicotinamide mononucleotide (NMN) is the rate-limiting step in local NAD synthesis. Thus, NAMPT activity is an important regulator of local NAD availability and regulator of downstream NAD-consuming enzymes [550]. NAMPT has been indicated in delaying senescence in human cells by making more NAD available [551] and possibly forms a direct connection between circadian rhythms and NAD salvage pathway, as NAMPT expression is regulated by the circadian rhythms machinery [552]. The final step in the NAD salvage pathway is catalyzed by NMNAT enzymes, similar to the conversion of NAMN to NAD.

The third pathway for NAD synthesis from nicotinamine riboside is initiated by phosphorylation of this molecule by enzymes nicotinamine riboside kinases (NRK) to form NMN. NMN is subsequently used for NAD synthesis by the NMNAT adenylyl transfer, as described above.

The last important source of NAD is the de novo synthesis—the conversion of amino acid tryptophan to NAD. These synthetic reactions start with the kynurenine pathway, which converts tryptophan to NAMN [553]. Clinically important is the fact that several enzymes of this biochemical pathway can be inhibited by vitamin B_2_ and B_6_ deficiency [554]. NAMN is transformed to NAAD (catalyzed by NMNAT) and, subsequently, to NAD (catalyzed by NADS), using identical reactions which convert NAMN to NAD in the Preiss-Handler pathway.

###### NAD Recycling

There is an ongoing loss of NAD, caused by the activity of NAD^+^-consuming enzymes/sirtuins, CD38, and poly(ADP-ribose)polymerases/, which enzymatically cleave NAD^+^ and typically produce nicotinamide. Such a high degree of NAD consumption cannot be compensated by a dietary intake. Instead, nicotinamide is recycled into NAD using the aforementioned salvage pathway that involves enzymatic activity of NAMPT and NMNAT. As most NAD is recovered in this manner, the activity of NAMPT has larger effect on NAD concentrations than nicotinamide levels or dietary intake of niacin [555].

###### Synthesis of NADP

NAD^+^ can be converted to NAD^+^ phosphate (NADP^+^)—a molecule with a different redox potential—by the activity of NAD kinase. In this reaction, one phosphate group, most often from ATP, is transferred onto the 2′-hydroxyl group of the adenosine ribose moiety of NAD. NADH can also be utilized as substrate, yielding NADPH instead of NADP^+^. NADP synthesis, therefore, critically depends on the availability of NAD and may be regarded as another NAD-consuming process, in addition to NAD-signalling reactions [556].

#### 4.3.3. Niacin Elimination

Consumed NA undergoes extensive first-pass metabolism in the liver by two separate metabolic pathways: the amidation pathway and the conjugation pathway [557]. The amidation is a high-affinity, low-capacity metabolic pathway [558]. It produces nicotinamide and pyrimidine metabolites, which have been connected to hepatotoxicity of niacin. The conjugative pathway is a low-affinity, high-capacity process and results in the formation of glycine conjugates of NA, such as nicotinuric acid (NUA). NUA has been suggested to be responsible for the vasodilation and flushing, typical side effects of high-dose niacin administered as a drug [557,559].

Because of the rapid absorption, the rate of absorption is strongly dependent on the pharmaceutical formulation, which determines the speed of release of niacin from the drug preparation. Immediate-release preparations have rapid dissolution and absorption of NA and saturate the low capacity amidation pathway, causing the majority of the drug to be metabolized into NUA. This, in conjunction with high peak concentrations of NA, result in a higher incidence of flushing. Sustained-release preparations release the drug slowly over time, causing most of the drug to be metabolized by the amidation pathway, which produces, however, greater amounts of hepatotoxic metabolites [557,558].

The liver methylates any remaining excess of nicotinamide to N^1^-methyl-nicotinamide, N^1^-methyl-2-pyridone-5-carboxamide, and other pyridone oxidation products, which are then excreted in the urine. Unmetabolized NA and/or nicotinamide might be present in the urine, as well, when niacin intakes are very high [560].

### 4.4. Physiological Functions of Niacin

The broad spectrum of functions of NAD and NADP can be divided into three distinct categories:

(A) The Cofactor: The first category involves enzymes, which utilize NAD and NADP as cofactors for reduction and oxidation (redox) reactions. In these redox reactions, both NAD and NADP oscillate between the reduced forms (NADH and NADPH) and the oxidized forms (NAD^+^ and NADP^+^). Even though the ratio of reduced/oxidized forms changes and depends on many factors, the total amount of NAD or NADP is not modified.

(B) The Substrate: The second category of NAD utilization is dependent on a group of NAD^+^-consuming enzymes. These enzymes only use the oxidized form NAD^+^ as a substrate in reactions that transfer the ADP-ribose part of the NAD molecule. Because these reactions require the enzymatic breakdown of NAD^+^, they consume NAD^+^ and lead to a decrease in total NAD^+^ concentrations available for other cellular reactions.

(C) The Ligand: Relatively recently discovered is the third type of NAD^+^ function, where it serves as a ligand for a group of purine receptors. NAD^+^ release, as a signaling molecule, has been observed in vascular smooth muscle cells, urinary bladder, gastrointestinal tract, brain, and neurosecretory cells [561,562,563,564]. Receptors sensitive to NAD^+^ were detected in monocytes, vascular endothelium, or colonic cells [565,566,567].

#### 4.4.1. Redox Reactions

Both NAD and NADP form a redox couple, which are critical cofactors in over 400 enzymatic reactions in most metabolic processes [414]. The redox couple NAD^+^/NADH is involved in mostly oxidative and catabolic reactions, while NADP^+^/NADPH is most often connected to reductive and anabolic reactions. This is enabled by a difference in redox potentials between the two couples. Under physiological conditions, the prevalent forms are oxidized NAD^+^ and reduced NADPH [424].

Examples of oxidative, catabolic reactions promoted by the oxidized NAD^+^ include production of pyruvate in glycolysis, production of acetyl coenzyme A, and complete catabolism in the Krebs cycle. These generate NADH, which drives the energy production through the mitochondrial respiratory chain. Furthermore, mitochondrial β-oxidation of fatty acids requires NAD^+^, as well. A general overview of catabolic processes involving NAD^+^ is presented in Table 9.

NAD^+^/NADH are cofactors for some anabolic reactions, as well (Table 9). At insufficient glucose concentrations, the enzymatic equilibrium of the enzymes in the glycolysis pathway is reverted so that they can utilize the reduced NADH, and the cell can synthetize glucose by gluconeogenesis from substrates, such as lactate, pyruvate, and acetyl coenzyme A. NADH functions as a cofactor in the synthesis of triglycerides (the synthesis of glycerol-3-phosphate) and in the synthesis of dihydrotestosterone from testosterone (as a cofactor of 5α-reductase) [568].

The reduced form NADPH is critical for many anabolic biochemical processes, too, for example, synthesis of cholesterol and fatty acids (Table 9). It is an essential cofactor in reductive reactions involved in glutathione/fatty acid peroxidation, cytochrome P450-mediated reactions, thioredoxin defense against oxidative stress, and in immune oxidative defense reactions [569]. The critical step in the synthesis of cholesterol is the synthesis of mevalonate from 3-hydroxy-3-methylglutaryl CoA (HMG-CoA) by HMG-CoA reductase (the known pharmacological target of statins), which requires NADPH as cofactor. Various synthetic reactions that originate from cholesterol also require NADPH: the synthesis of bile acids and steroid hormones.

The erythrocytes contain high concentrations of NADPH, probably due to the fact that red blood cells lack mitochondria. This availability of NADPH allows continuous activity of glutathione reductase, which, in turn, allows glutathione/fatty acid hydroxyperoxidase enzymes to reduce the oxidative stress in the iron and oxygen-rich environment of erythrocytes.

The redox couples NAD^+^/NADH and NADP^+^/NADPH also play critical role in the degradation of ethanol and other alcohols. Through several metabolic reactions, ethanol is converted to acetaldehyde and then to acetate. The conversion to acetaldehyde can be catalyzed either by the cytochrome P450 system 2E1 (with consumption of O_2_ and NADPH, and production of superoxide) or the enzyme alcohol dehydrogenase (ADH, producing NADH from NAD^+^). The following oxidation of acetaldehyde to acetate is catalyzed by aldehyde dehydrogenase (ALDH) and also requires NAD^+^ [570]. Although metabolism by ADH is the preferential route, higher or chronic alcohol consumption will induce the ethanol metabolism by the P450 route, which increases the risk of hepatotoxicity and decreases the levels of NAD^+^. If this decrease in NAD^+^ and increase in NADH concentrations are significant, the enzymatic equilibrium of NAD-dependent enzymes is shifted. Such shift in NAD^+^/NADH ratio can lead to accumulation of lactate and inhibition of gluconeogenesis, hypoglycemia, and inhibition of the energy production in the Krebs cycle in ethanol-detoxifying tissues [424].

Both NAD^+^/NADH and NADP^+^/NADPH are critical for function of the cellular respiratory chain in all cells. NAD and NADP are present in higher concentrations in mitochondria compared to other subcellular compartments, and mitochondria-rich tissues, such as myocardium, contain more NAD than tissue with fewer mitochondria (e.g., liver).

The role of NAD forms in redox reactions is strongly dependent on the ratio of oxidized (NAD^+^/NADP^+^) to reduced (NADH/NADPH) molecules, and changes in this ratio can lead to shift in many enzymatic reaction equilibria. Furthermore, this ratio is continuously affected not only by the activity of several enzymes of NAD metabolism but also by all cellular metabolic processes (the Krebs cycle, glycolysis, gluconeogenesis, fatty acid synthesis, mitochondrial respiratory chain, etc.). The redox state of NAD/NADP represents a complex sensor of biochemical energy metabolism in any given cell and situation. NAD, therefore, serves as an indicator molecule with a capability to integrate information about overall energy production, energy consumption, and nutrition.

#### 4.4.2. NAD as Substrate

Intricately connected to the function of NAD as an indicator of cellular metabolism is the fact that NAD^+^ is the substrate for several enzymes which regulate cellular epigenetic information. NAD^+^ is the form whose concentrations fluctuate most significantly and best reflect various metabolic situations. NAD^+^ level changes with nutritional intake, exercise, and with circadian rhythm [571]. This close connection of NAD^+^ to energy metabolism has provoked an interesting idea that increased NAD^+^ availability in skeletal muscle could enhance oxidative phosphorylation, and that NAD^+^ supplementation could be used for therapeutic goals to improve energy metabolism [572,573].

In addition to being influenced by metabolic state, NAD^+^ concentrations are directly regulated by NAD^+^-consuming enzymes. This allows for direct regulation of metabolic processes in response to stimuli and situations that activate these NAD^+^-consuming enzymes. Several groups of enzymes that continuously consume NAD have been identified. These are sirtuins, poly(ADP-ribose) polymerases (PARP) and ADP-ribosyl cyclase.

#### 4.4.3. ADP-Ribosyl Cyclases

The group of ADP-ribosyl cyclases comprise the enzyme CD38 and its homologue CD157. Their function is to generate cyclic ADP-ribose from NAD^+^, concomitantly releasing nicotinamide [574,575]. Both proteins have been regarded as immune cell activation marker because they are expressed in neutrophils and endothelial cells, and they are involved in signal transduction associated with immune response. The synthesized cyclic ADP-ribose serves as a second messenger that activates intracellular signaling pathway, resulting in an increase of intracellular Ca^2+^ concentrations by activating receptors on the endoplasmic reticulum [569]. Lack of CD38 in mice leads to a defect in immune cell migration to inflammatory sites and increased rate of infections [576].

Cyclic ADP-ribose and Ca^2+^ release caused by CD38/157 are also involved in regulation of oxytocin release and may play a role in regulation of social behavior. Expression levels and genetic polymorphism of CD38/157 have been associated with autism spectrum quotient, and the dysfunction of these enzymes caused by lack of NAD^+^ may be connected to the dementia and psychosis typical for pellagra [577].

#### 4.4.4. Poly(ADP-Ribose) Polymerases (PARP)

Enzymes of the PARP superfamily generate ADP-ribose posttranslational modification at specific sites in target proteins. They cleave the glycosylic bond between nicotinamide and ribose, break down NAD^+^, release nicotinamide, and attach the ADP-ribose moiety to target protein.

So far, 17 enzymes of the PARP group have been identified in humans [578,579,580]. Only several of those have been shown to be true polymerases (PARP1, PARP2, PARP5a, and PARP5b), i.e., to keep adding more ADP-ribose units to the first attached ADP-ribose in subsequent steps, thus creating polymers of ADP-ribose (PAR) as posttranslational modification. The enzymatic activity of majority of the PARP members is limited to attaching only one ADP-ribose unit.

The most active and best characterized PARP enzyme is PARP1. PARP1 is activated after detection of and binding to DNA strand breaks. Activated PARP1 consumes NAD^+^ and attaches large PAR molecules to target proteins (including PARP1 itself, where it inactivates the enzymatic activity) [580]. This negative feedback loop, together with the rapid degradation of PAR by glycohydrolases, represents an effective, short-term inhibition of cell cycle progression as long as DNA damage is present, to allow sufficient DNA repair and prevent division of damaged cells. However, excessive DNA damage leads to hyperactivation of PARP1 and leads to NAD^+^ depletion and cell death. This mechanism has been regarded as a cancer prevention protocol [581]. On the other hand, supressing such hyperactivation of PARP1 and the associated severe depletion of NAD^+^ protects from cell death after inflammation or ischemia/reperfusion injury [582,583,584].

Pharmacological inhibition of PARP1 by drugs, such as olaparib, niraparib, and talazoparib, was recently approved for treatment of several forms of cancer. This therapeutical approach takes advantage of a defect in homologous recombinational repair (HRR) of the double-strand DNA breaks pathway, which was detected in multiple tumor types [585]. Inhibition of PARP1 leads to accumulation of unrepaired single-strand DNA breaks, which leads to formation of double-strand DNA breaks. These can be effectively repaired in cells with intact HRR; however, “paribs” cause lethality in tumor cells with HRR deficiencies [586,587].

This role of ADP-ribosylation in DNA repair is well known, but the function of these NAD^+^-consuming enzymes expands beyond this accepted view. In addition to DNA-repair, ADP-ribosylation is involved in regulation of DNA replication and cell division, transcription, responses to infection, stress, and aging [588]. This wide array of functions is based on modulation of many protein interactions, control of transcription, and epigenetic control.

#### 4.4.5. Sirtuins

Sirtuins are members of the family of class III histone deacetylases (HDACs) that require the breakdown of NAD^+^ for their enzymatic activity. So far, seven sirtuins homologues have been identified in mammals (Sirt1–Sirt7) [589]. As changes in NAD^+^ availability and NAD^+^/NADH redox ratio directly influence their catalytic activity, sirtuins function as sensors for cellular energy and redox status that directly affect chromatin structure.

**Figure 11:**
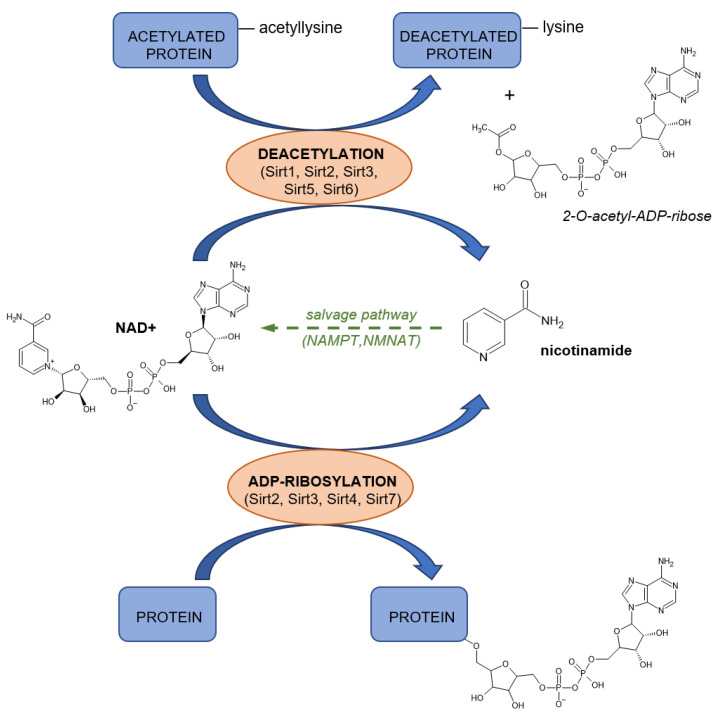
Mechanisms of NAD^+^ depletion by sirtuins. NAD^+^ is required for two types of protein modifications catalyzed by sirtuins. Nicotinamide is cleaved off in the process and regenerated into NAD via the salvage pathway.

The main function of sirtuins as HDACs is the deacetylation of lysin residues on target proteins. This is the preferred activity for Sirt1–Sirt3, Sirt5, and Sirt6. In the first step of this process, sirtuins cleave the nicotinamide moiety off an NAD^+^ molecule. Subsequently, the acetyl group from target protein is transferred to the remaining ADP-ribose, resulting in deacetylated protein and 2-O-acetyl-ADP-ribose (Figure 11) [590]. No deacetylation activity has been detected in Sirt4 and Sirt7: these enzymes utilize NAD^+^ to transfer ADP-ribose groups to target proteins. This enzymatic activity is shared with Sirt2 and Sirt3 (in addition to their deacetylase activity).

Sirtuins are connected to NAD^+^ in several ways. (1) Their activity directly depends on the levels of available NAD^+^, as well as increasing levels of NADH or nicotinamide directly inhibit sirtuins activity. This represents a direct NAD^+^ and redox status sensor mechanism. (2) Sirtuins compete for available NAD^+^ with other NAD^+^-consuming enzymes. Enzymes with high capacity for NAD^+^ utilization, such as PARP1, can deplete NAD^+^ to concentrations which change the activity of sirtuins, especially in limited subcellular microdomains. (3) Sirtuins have been reported to regulate the circadian transcription factors CLOCK and BMAL1, which, in turn, regulate the expression of NAMPT, the critical enzyme in NAD synthesis. This forms a regulatory loop that leads to circadian oscillations of NAD^+^ and is directly connected to Sirt1 and Sirt6 [571,591,592].

The general result of sirtuins activity is the change in epigenetic information in the form of altered chromatin structure, as well as direct regulation of transcription factor activities. Specifically, sirtuins target transcription factors that regulate oxidative metabolism, antioxidant defense, and gene expression in mitochondria [593]. The ubiquitous expression and differential subcellular localization further allow sirtuins to modulate myogenesis, gluconeogenesis, insulin secretion, adipogenesis, DNA repair, and senescence [594,595,596,597].

In summary, there is a complex interplay of metabolism, nutrition, and circadian rhythm, where NAD^+^ plays a central role, connecting metabolic and dietary information with transcriptional regulation. The collective activities of NAD^+^-consuming enzymes represent a mechanism integrating cellular signaling with the chromatin control and epigenetic regulation and allowing adjusting of transcription and gene expression in response to change in environmental conditions.

### 4.5. Niacin Deficiency

Niacin deficiency was first described in 1735 by the French physician Francois Thiery and Spanish physician Gaspar Casal. The first reference to this disease as “pellagra” (“rough skin”) appears in 1771 [598]. Pellagra became characterized as “3 Ds” or “4 Ds” disease which refer to the three common symptoms (dermatitis, dementia, diarrhea) possibly culminating in death (the 4th D). Pellagra epidemics had appeared in human population for several hundred years, mostly as a result of insufficient vitamin B_3_ intake due to monotonous diet (especially unprocessed corn). The methods for analytical detection of vitamin B_3_ levels in biological fluids are summarized in Table 3 and Supplementary Data Table S1.

Detailed epidemiology and symptomatology of pellagra has been reviewed elsewhere (e.g., References [599,600]). Pellagra is still present in developing countries in connection with niacin-deficient diet or emergencies [601,602,603]. Presently, in the EU or USA, pellagra is very rare due to fortification of food with B vitamins. The most common causes of niacin deficiency are alcoholism (35%), medications (26%), and insufficient intake (16%) or malabsorption (13%) [554]. Treatment of pellagra includes dietary changes and supplementation of vitamin B_3_ (NA, or nicotinamide) in doses of 10–300 mg/day, up to 1000 mg/day in most severe cases. Nicotinamide is preferred to NA in B_3_ supplementation therapy, as it does not cause vasodilation and flushing and has lower toxicity, in general.

### 4.6. Pharmacological Use of Niacin

NA and other forms of vitamin B_3_ have been in use as pharmacotherapeutics for several decades. However, recent progress, in our understanding, of underlying molecular mechanisms of different diseases has led to possible indications of niacin therapy in pathologies beyond its original use in treatment of pellagra and dyslipidaemia. Furthermore, it is important to distinguish the administration of any form of vitamin B_3_ to counteract niacin deficiency (i.e., pellagra) from the use of NA in supraphysiological, pharmacological doses in therapy of different pathological conditions.

#### 4.6.1. Atherosclerosis, Dyslipidaemia and Cardiovascular Risk

More than 60 years ago, Rudolf Altschul demonstrated that supraphysiological doses of NA (but not nicotinamide) have positive effects unrelated to those physiological associated with the function of the vitamin [604]. Administration of gram quantities of NA (1000–3000 mg/day, up to 6000 mg/day) reduce total cholesterol plasma concentrations, decrease low-density lipoprotein (LDL) cholesterol levels and increase high-density lipoprotein (HDL) cholesterol levels, while reducing total mortality in treatment of coronary artery disease [605]. Research since then has indicated that these effects are mediated by agonistic action of NA on nicotinic acid receptor.

##### Nicotinic Acid Receptor GPR109A

NA receptor GPR109A belongs to the family of G-protein-coupled receptors (GPCRs) of the class A rhodopsin-like GPCRs and is coupled to the G_i_ family. GPR109A is expressed in significant levels in both white and brown adipose tissue, in the spleen, and immune cells, such as macrophages, monocytes, dendritic cells, and neutrophils [606,607]. In immune cells, G_i_ activation generally leads to activation of phospholipase Cβ or phosphoinositide 3-kinase. Its role in immune cells remains unclear.

**Figure 12:**
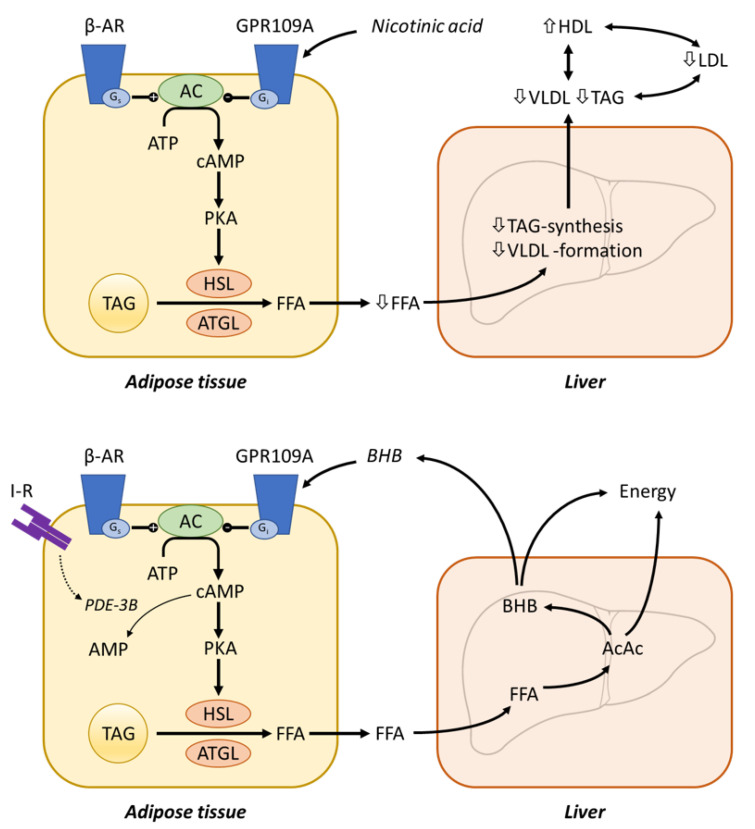
GPR109A receptor function. (upper panel) Metabolic effects of nicotinic acid. Activation of nicotinic acid receptor inhibits the mobilization of free fatty acids (FFA) from adipocytes and lowers the production of VLDL in hepatocytes with consequently decreased plasma levels of VLDL/LDL. TAG inhibit the cholesterol transfer to HDL via cholesteryl ester transfer protein (CETP). (bottom panel) Possible physiological role of GPR109A. In fasting, increased activity of the sympathetic nervous system and low insulin levels stimulate breakdown of TAG into FFA. Hepatic metabolism produces β-hydroxybutyrate (BHB) and acetoacetate (AcAc) as energy sources. BHB forms a negative feedback loop that limits the fat catabolism in adipose tissue.

In other cell types, including adipocytes, activation of G_i_ has an inhibitory effect on adenylyl cyclase and decreases intracellular cAMP levels. Activation of GPR109A by NA, therefore, counteracts the effect of G_s_-coupled receptors (such as β-adrenergic receptors, β-AR), whose activation increases cAMP concentrations and results in activation of protein kinase A. In adipocytes, protein kinase A phosphorylates several proteins, notably proteins required for triacylglycerol hydrolysis: hormone-sensitive lipase (HSL) and perilipin. Phosphorylated perilipin allows the phosphor-activated HSL (and another lipase—adipose triacylglycerol lipase, ATGL) access to the lipid droplets containing triacylglycerols. These lipases then hydrolyze triacylglycerols into free fatty acids (FFA) and glycerol (Figure 12 upper panel).

Several derivates of niacin have been developed that selectively activate GPR109A, and large efforts have been made to identify the endogenous ligands of this receptor. NA itself is unlikely to act as an endogenous agonist, as its physiological plasma concentrations are generally low. Activation of GPR109A has been demonstrated by β-hydroxybutyrate, a ketone compound produced by the liver during starvation [608]. Β-hydroxybutyrate acts as an agonist of GPR109A with EC_50_ of approximately 750 μM, even though other ketones, such as acetone or acetoacetate, have no activity on this receptor. This indicates a physiological role of nicotinic acid receptor GPR109A under starvation. Under such conditions, a high activity of the sympathetic nervous system stimulates the β-adrenoreceptors of adipocytes, while the levels of insulin are low. This leads to increased intracellular cAMP levels and stimulation of lipolysis. Released FFAs are metabolized in the liver to ketones, including β-hydroxybutyrate and acetoacetate, which serve as the energy source. Concentrations of β-hydroxybutyrate reach millimolar range under starving conditions and by activation of GPR109A β-hydroxybutyrate inhibits lipolysis [609]. In this way, β-hydroxybutyrate may counteract pro-lipolytic stimuli during starvation, and this mechanism forms a negative feedback loop to conserve energy when fasting (Figure 12 bottom panel).

##### Mechanisms of Nicotinic Acid Action

The antilipolytic effect of NA is likely similar to that of β-hydroxybutyrate and is mediated by activation of the GPR109A receptor, inhibition of cAMP/PKA signaling cascade, inhibition of lipolysis, and of release of FFA from adipocytes. This seems to be supported by temporal differences of NA effects on individual types of lipid particles. Administration of NA leads to very rapid (within minutes) decrease in plasma FFA levels. This is followed by a reduction in VLDL and triacylglycerols (2–4 h), and many days later, by a decrease in LDL concentrations [610]—a time-course which follows the established biochemical relationships.

**Figure 13:**
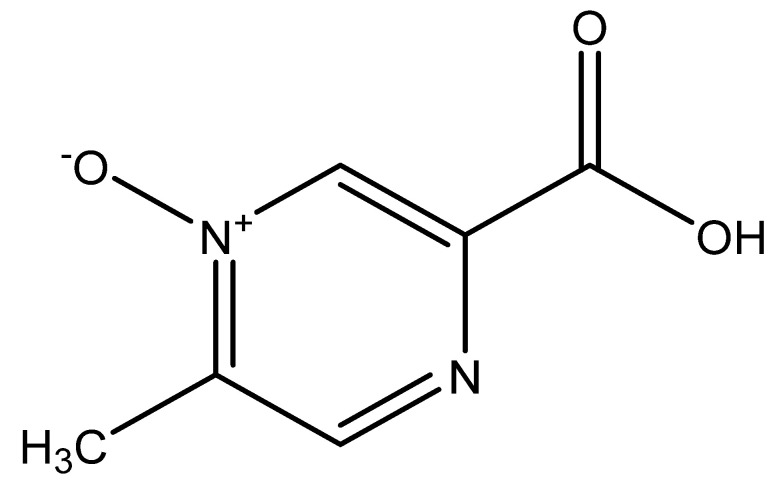
Structure of acipimox, a synthetic derivative of nicotinic acid used as a lipid lowering drug.

The activation of the GPR109A receptor with inhibition of adipose cell lipolysis seems to be the main mechanism of action of NA in dyslipidaemia and atherosclerosis; however, several alternative/complementary mechanisms have been proposed:

(1) Cell culture experiments suggested that NA inhibits the synthesis of triacylglycerols through direct inhibition of diacylglycerol acyl transferase 2 (DGAT2) in hepatocytes [611]. This, in turn, stimulates the degradation of ApoB and leads to decreased formation of ApoB-containing VLDL [612]. This effect on DGAT2 was, however, detected only in NA concentrations which were approximately 100-fold higher than plasma concentrations reached after maximal pharmacological doses of NA [613].

(2) The mechanism by which NA increases HDL concentrations is presently not clearly understood. The currently accepted hypothesis claims indirect interference with the exchange of triacylglycerols and cholesterol between VLDL/LDL and HDL, which is mediated by cholesteryl ester transfer protein (CETP). CETP promotes bidirectional transfer of cholesteryl esters from HDL to VLDL/LDL in exchange for TAG from VLDL/LDL to HDL [614]. Due to the decrease in triacylglycerol content in VLDL and LDL after the treatment with NA, this exchange of TAG and cholesterol is expected to decrease, resulting in increased HDL-cholesterol levels. This is supported by observations from mice, which physiologically do not express CETP [615], and by experiments where inhibition of CETP resulted in similar increase in HDL levels [616,617]. In any case, this HDL-increasing effect seems to be mediated by GPR109A. Acipimox (Figure 13), a synthetic derivate of nicotinic acid and an agonist of GPR109A, has similar spectrum of effects as NA (including an increase in HDL), while nicotinamide, which is not a GPR109A agonist, has no effect on plasma cholesterol [618].

(3) Another proposed mechanism by which NA increases HDL levels is inhibition of catabolism of ApoA-containing lipoproteins [619,620]. Again, these cell culture studies reported these effects at NA concentration much higher than the usual therapeutic plasma concentration of NA.

(4) In addition to the described effects on LDL/HDL concentrations, a direct antiatherosclerotic effect of NA has been suggested. In adipocytes [621] and “non-foam” macrophages [622], NA stimulates GPR109A, which leads to an increased expression of transcription factors peroxisome proliferator-activated receptor γ (PPARγ) and liver X receptor α (LXRα). PPARγ and LXRα, in turn, enhance the transcription of ATP-binding cassette A1 (ABCA1) and G1 (ABCG1) transporter proteins, which are responsible for in vitro and ex vivo cholesterol unloading [623,624] and are directly implicated in the regression of experimental atherosclerosis [625,626]. Given these observations and the documented expression of GPR109A in peripheral macrophages, it is feasible that NA exerts its antiatherosclerotic effects, at least in part, by stimulating the removal of cholesterol from macrophage foam cells in atherosclerotic lesion and by stimulation of reverse cholesterol transport.

#### 4.6.2. Aging

Aging is a complex process that involves many pathophysiological changes. As the two major contributors to aging are currently accepted to be accumulation of DNA damage, and mitochondrial dysfunction resulting from DNA damage. Because both of these factors are dependent on NAD availability, it has been stipulated that the NAD^+^ levels represent a link between aging and altered metabolism [593,627,628,629,630,631].

Data from various animal models suggest a strong correlation between age and decline in NAD^+^ levels [632,633,634]. Factors responsible for this nutrition-independent NAD^+^ loss are increased activity of sirtuins, CD38 and PARPs [635,636]. The increased PARP activity is a response to accumulation of DNA damage and contributes to the NAD^+^ depletion in aging. At the same time, inhibition of PARP1 [552,637] or restoration of NAD^+^ levels [638,639], at least in some models of aging, resulted in improvement of the aging phenotype. This reduced NAD^+^ availability inhibits normal sirtuins activity, which is critical to prevent mitochondrial dysfunction and age-related changes in metabolism [595].

It is unclear whether NAD^+^ precursor supplementation improves mitochondrial function and facilitate DNA repair in an aging human population, and if it synergizes with small molecule activators of sirtuins to further increase the health span during aging.

#### 4.6.3. Cancer and Cell Death

Through mechanisms similar to those described above, genotoxic stress causes rapid depletion of NAD^+^, which can lead to cell death. Direct NAD^+^ supplementation in cell cultured neurons protected them from apoptotic death [640], and increased mitochondrial NAD^+^ could prevent cell death induced by general NAD^+^ depletion [555].

Furthermore, NAD^+^ depletion causes a shift in enzymatic equilibria and results in changes in cell metabolism (the Warburg effect). Unlike in aging situations, where the aim is to protect the cells from such dysregulations, several studies have explored the possibility of increasing cytotoxic effects of established anti-cancer drugs with pharmacological NAD^+^ depletion. NAD^+^ reduction sensitizes cancer cells to oxidative damage by disrupting their antioxidant defense system, induces cell death by preventing DNA repair, and shifts signaling pathways towards a cytotoxic direction [641,642].

#### 4.6.4. Neurological Disorders

##### Parkinson’s Disease

A basic characteristic of Parkinson’s disease (PD) is the degeneration of dopaminergic neurons in the substantia nigra, which leads to dysregulation of motor function and development of typical symptoms caused mostly by the insufficient dopamine synthesis.

Dopamine synthetic pathway requires several coenzymes, one of which is NADPH. A direct link between NAD and PD has been established. PD patients generally have low blood concentrations of NAD compared to healthy subjects, and these low NAD concentrations correlated with high expression of GPR109A [643]. Dietary supplementation of niacin normalized both NAD levels and GPR109A expression and was followed by improvement of cognitive and motor functions of PD patients [644]. Similar positive effects of exogenous NAD were described in cell-culture human PD model, where increased NAD concentrations exerted protective effects against mitochondrial dysfunction and oxidative damage [645].

Several mechanisms of this neuroprotective effect of NAD in PD (and potentially other neurodegenerative diseases) have been proposed, e.g., increased activity of Sirt1, decreased activity of PARP1, restoration of mitochondrial function and mitochondrial biogenesis, restoration of redox NAD^+^/NADH balance and suppression of oxidative stress, decreased toxicity of misfolded proteins and DNA damage, and increased activity of neurotrophic factors and signaling pathways [428].

###### Multiple Sclerosis

Progressive neurodegeneration associated with inflammation is the basic pathoetiological mechanism in multiple sclerosis. Notwithstanding large progress, current therapeutical options targeting mostly the inflammation are not always efficient to prevent the progress of the disease and stop the neurodegeneration. Experimental data suggest contribution of NAD^+^ depletion and oxidative stress induced mitochondrial dysfunction to the development of multiple sclerosis (reviewed in Reference [646]). Shortly, both increased activity of NMNAT or Sirt1 resulted in neuroprotective effects in models of multiple sclerosis and indicates that pharmacological intervention aimed at increasing the NAD^+^ levels in neurons should be studied in more detail.

###### Schizophrenia

Patients suffering from severe cases of pellagra develop pellagrous dementia, which is similar to schizophrenia. Even though schizophrenic patients generally do not improve with increased vitamin B_3_ intake [449], subpopulations of patients exist who do respond to niacin supplementation therapy. These observations suggest some similarities in underlying mechanisms of niacin-deficient and schizophrenic brains. One study reported that chronic schizophrenic patients display a significantly reduced NAD^+^/NADH ratio [647], which would indicate a link between the etiology of schizophrenia and redox imbalance in the brain and NAD^+^ deficiency.

#### 4.6.5. Skin Cancer Prevention

Another typical symptom of niacin deficiency is skin defects, such as photosensitivity, leading to rash in sun-exposed areas, hyperkeratosis, and dermal fibrosis. The treatment with niacin and restoration of physiological NAD^+^ levels allow the skin to recover. NAD-deficient human keratinocytes have lower growth rates, increased rates of apoptosis, and increased degree of oxidative stress that leads to increased rates of DNA damage. Furthermore, reduced NAD^+^ availability results in photosensitization in skin cell cultures, with reduced DNA damage-induced PAR formation and sirtuin expression levels [648]. Experiments in animal models have demonstrated that moderate niacin deficiency increases the incidence of skin cancer in response to UV-B treatment in mice, and oral administration of nicotinamide could inhibit photocarcinogenesis [649].

This indicates that even mild vitamin B_3_ paucity, often seen in humans, could increase the skin cancer risk. The positive effects of niacin for skin function in clinical setting have been long known. Nicotinamide improves wound healing, causes reduction of wrinkle depth and smoothing of the skin surface structure, improves the epidermal barrier function, reduces light damage to the skin, and is used for treatment of psoriasis [650,651]. Another study has demonstrated protective effect of oral nicotinamide against recurrence of certain types of skin cancer [652].

### 4.7. Toxicity of Niacin

The most serious side effect of pharmacological doses (up to 6000 mg/day) of NA is flushing, with incidence as high as 80% on initial exposure [653]. The flushing is caused by cutaneous vasodilation, which is restricted to the chest and face and is often accompanied by burning sensation and pruritus. This side effect is bi-phasic: the first fast phase occurs in 20–30 min, followed by slow second face after 40–60 min.

The flushing is mediated by increased cutaneous production of prosthanoids. NA activates GPR109A receptor on dendritic cells or macrophages and induces the production of arachidonic acid by phospholipase A_2_ [654]. The first phase is mediated by increased concentrations of prostaglandin D_2_ (PGD_2_) and activation of DP_1_ receptor. The delayed second phase is thought to be induced by the dermal PGE_2_ keratinocyte pathway [655] or other mediators, e.g., histamine, substance P, adrenomedullin, or calcitonin-gene related peptide [656].

The flushing is responsible for discontinuation of NA therapy in 50% of patients [653] and represents the most serious drawback of NA use in therapy of atherosclerosis and dyslipidaemia. Various approaches have been used to minimize this side effect. The flush incidence and degree correlate with the peak plasmatic NA concentrations (c_max_) and are most common in drug formulations with immediate or rapid release. To limit the occurrence of flushing, NA dose can be timed so that it develops at night and is less distressing. Slow-release formulations which liberate NA over 12 or 24 h have reduced the incidence of flushing by up to 60–80% [657] but also have lower antihyperlipidemic and antiatherosclerotic effects and a higher degree of hepatotoxicity [658], indicated by elevated plasma transaminase activity [659,660].

Another approach to reduce flushing after NA is to reduce the production and effects of PGD_2_. Cyclooxygenase inhibitors, such as acetylsalicylic acid or indomethacin, were successfully used to improve tolerability of NA in some patients but are not universally effective [653]. Use of a DP_1_ receptor antagonist—laropiprant—has been more successful. Its main effect is the reduction of the first phase of flushing by 80% [661], which has led to combined drug formulation of NA and laropiprant.

Further side effects of NA, other than the flushing, include headache, heartburn, peptic ulcers, nausea and vomiting, lactic acidosis, and hypotension [609,662]. Reports indicate that NA increases insulin resistance, and concerns have been raised about the use of NA in diabetic patients [663,664]. Although the GPR109A agonist acipimox does not induce insulin resistance, there are distinct differences in its FFA-lowering effects compared to NA, and it remains unclear whether the NA-induced insulin resistance is a side effect of GPR109A agonists as a class, or a specific side effect of NA. Analysis of risk-benefit ratio in diabetic patient in several clinical studies has subsequently demonstrated that the use of NA in controlled type 2 diabetic patients is safe and only marginally increases glycemia or glycated hemoglobin (HbA1c) [665,666,667].

With the new trends in the development and novel application of nicotinamide in therapy of several pathological conditions, safety concerns regarding long-term use of nicotinamide have been raised. Nicotinamide produces a broad spectrum of diverse pharmacological effects, including influence on energy metabolism, DNA repair, protein translation, or epigenetic control. However, there is currently a lack of assessment of potential adverse effects of nicotinamide, and the individual reports are scarce. A recent review [668] addressed some of these concerns but raised more questions and emphasized the need for systematic evaluation of nicotinamide safety profile.

## 5. Pantothenic Acid—Vitamin B_5_

### 5.1. Introduction and Properties

Vitamin B_5_, also known as pantothenic acid, is a water-soluble vitamin ubiquitously found in food. The pantothenic acid was discovered in 1931 by Dr. R. J. Williams during his studies with *Saccharomyces cerevisiae* [669]. He presented evidence for the existence of an unidentified nutrient stimulating the growth of the yeasts in a very striking way, and, in 1933, he named it by using a Greek word “panthos”, which means “everywhere” [670]. Later, experiments with pantothenic acid-deficient animals proved its fundamental importance, which resulted in its classification as a vitamin.

Pure pantothenic acid is a viscous liquid. It has a pKa of 4.41. It is more stable in a slightly alkaline than in an acidic environment, where it will be hydrolyzed; maximum stability is at pH 5–7. Its degradation is accelerated by heat. It is moderately stable to atmospheric oxygen and light when protected from moisture. Pantothenic acid is usually administered as calcium pantothenate, which is solid and more stable than pantothenic acid against the light, heat, and oxygen, but unstable to both alkaline and acidic conditions. Another solid form of pantothenic acid is sodium pantothenate. Its use, however, is limited due to its hygroscopicity [69,415,671,672,673].

### 5.2. Sources of Pantothenic Acid

The biosynthesis of pantothenic acid occurs in plants, fungi, and most bacteria. The human or animal organism as well as some bacteria lack the capability to synthesize this vitamin and, therefore, they are dependent on its exogenous supply [14,423,431,540,671,672,674,675,676,677,678,679,680,681,682,683,684,685,686]. In the human diet, pantothenic acid is ubiquitous and is widely distributed in foods of both plant and animal origin. Major sources include meat, offal (liver and kidney), eggs, milk, cheese, nuts, mushrooms, yeast, whole grain cereals, legumes, cruciferous vegetables (such as broccoli or cauliflower), avocado, potatoes, and tomatoes [445,672,687,688,689,690,691,692,693]. The main contributors to pantothenic acid intakes are meat products, bread, milk-based products, and vegetables because of the quantities consumed [471,692,694]. One of the richest natural sources of pantothenic acid is royal jelly [672,692,695,696,697]. The contents of pantothenic acid in some foodstuffs are shown in Table 10. Besides foods, it is possible that gut microbiota in the large intestine likely contribute to the overall pantothenate supply in humans; however, currently, the extent of this contribution is not known [14,672,690,698,699,700,701,702,703]. Regardless, pantothenic acid is unambiguously synthetized de novo by the gut microbiota in the colon. This complex reaction needs two basic building blocks: 2-dihydropantoate and β-alanine. Magnúsdóttir et al., in 2015, predicted human gut bacteria with the ability to synthesize pantothenic acid. This prediction is based on the structure of the bacterial genome containing the functional enzymes needed for pantothenate and CoA biosynthesis. The ability to produce pantothenic acid is present in nearly all Bacterioides and Proteobacteria. These strains are also able to continue with biosynthesis till CoA. Pantothenic acid can be also synthesized by a few Actinobacteria and Firmicutes. On the other hand, some bacterial strains, e.g., *Fusobacteria*, are not able to produce pantothenic acid. This was subsequently proven experimentally, and, indeed, *Bacteroides fragilis*, *Bacteroides thetaiotaomicron*, *Bacteroides vulgates*, *Escherichia coli*, *Helicobacter pylori*, *Klebsiella pneumonia*, *Listeria monocytogenes*, and *Salmonella enterica* are able to synthetize vitamin B_5_ [423].

Food processing may alter the content of pantothenic acid [415,688]. The milling of cereals, in which grains, such as wheat, rice, and corn, are dehulled and ground into smaller pieces or flours to improve palatability, reduce cooking time, and create food products, but remove grain parts rich in micronutrients, resulting in considerable losses of pantothenic acid [99,109,445,464,687,704]. Milling reduces pantothenic acid contents, in comparison to whole cereals, by 50–55% and 64–88% in wheat and maize, respectively [63,99,705]. Pantothenic acid losses are 50–67% and 18–25% in non-parboiled and parboiled white rice, respectively, compared to brown rice [61,99,109,705].

Pantothenic acid is quite stable during thermal processing at pH levels of 5–7; losses of pantothenic acid during the preparation and cooking of foods are normally not very large [69,415], but substantial ones can occur through leaching into the cooking liquids, such as water, soup, gravy, or drippings; when those are consumed along with the cooked food, a great part of the vitamin is retained [70,73,74,706,707,708]. Pantothenic acid content in pork, beef, and chicken is reduced owing to steaming, braising, and, in particular, by boiling, by 15–50% solely in meat due to leaching. In the whole dish, the losses are only 10–20%. Frying decreases the vitamin level by 20%, and it only decreases by 10% when the meat is breaded [70]. Similarly, a decrease in pantothenic acid in fish during cooking by different methods comes about [70,102,706]. Steaming, boiling, baking, and frying of potatoes with the peel bring on pantothenic acid losses of 10% in all cases, but the losses might reach 30% in peeled potatoes when boiled [70,73]. In addition, in vegetables, boiling and steaming usually causes declines of 10% in the total dish, and those of 30–40% and 15%, respectively, in vegetables alone [70,73,98,709]. Stewing, frying, and baking lessen pantothenic acid amounts in vegetables by 10% [70,73]. Pantothenic acid losses of 24–67% in legumes during boiling are influenced by the pre-soaking method and cooking times [74,710,711] Boiling of rice results in a decrease of 59–66% in pantothenic acid content [99]. That is why steaming is preferred to boiling, in particular, when cooked vegetables are eaten without cooking liquids [70,98,709,712]. Poached, boiled, and fried eggs lose, due to cooking, 4%, 7%, and 9% of their pantothenic acid, respectively [107]. In milk, pantothenic acid is stable during pasteurization, since the normal pH of milk is within the optimal pH stability range; milk generally loses less than 10% during processing [74,415,713].

In breadmaking, no significant difference of pantothenic acid was observed during the kneading phase, while a mild decrease of 12% was documented during baking. This indicates that pantothenic acid is more sensitive to heat than to light and oxygen [508]. The roasting of peanuts at 160 °C and 180 °C decreases the amount of pantothenic acid by 24% and 92%, respectively; so, peanuts can be an excellent source if properly processed [726].

Canning leads to various reductions in pantothenic acid content: 1–43% in pork luncheon meat, depending on times and temperatures used during thermal processing [125]; 20–35%, 46–78%, and 51%, in foods of animal origin (such as meats, fish, and dairy products), vegetables, and fruits and fruit juices, respectively [705]. Thermal degradation kinetics of pantothenic acid in extracts of *Averrhoa bilimbi* fruits showed that increasing the temperature speeds up the decomposition, which was also linearly time-dependent [727]. Treatment of food with ionizing radiation used as a method for its preservation has insignificant effects on pantothenic acid content [124,477]. Less pantothenic acid is in food products based on nixtamalized (i.e., alkali-treated) maize [62,488].

Effects of storage conditions on pantothenic acid amount in milk powders [728] and in berry juice of *Hippophaë rhamnoides* (sea buckthorn) [729] have also been studied. The stability was affected to different extents depending on time, temperature, moisture, and oxygen presence during storage.

Lower contents of pantothenic acid in frozen foods, compared to those in raw ones, have been reported; decreases were 18–63% in vegetables, 29–71% in legumes, 7% in fruits and fruit juices, and 4–55% in fish [672,690,705]. After thawing frozen meat, pantothenic acid, together with other B vitamins, transfer in a drip; amounts of pantothenic acid from defrosted meat found in the drip were 7% and 33% in pork and in beef, respectively. For prevention of the loss of the vitamin, collection and use of the drip is recommended [707,730,731].

Pantothenic acid (mostly in the form of the calcium salt but also as pantothenyl alcohol) is commercially produced for use in the food, pharmaceutical, and cosmetic sectors, as well as in animal feeds (for the last purpose, about 80% of the produced amount are used) [671,672,732]. At present, industrial production of pantothenic acid is based on a combination of chemical and enzymatic reactions. Biocatalytic steps are especially important for circumventing the expensive and troublesome chemical racemic resolution of optical isomers because only ®-isomer of pantothenic acid possesses biological activity [171,671,672,732,733]. Pantothenic acid is obtained via condensation of two key building block®(R)-pantolactone and β-alanine. Racemic pantolactone is synthesized from isobutyraldehyde, formaldehyde, and hydrogen cyanide. Several chemical and microbiological methods have been developed for the separation of (R)-pantolactone. One of the commercialized biocatalytic processes is carried out with immobilized cells of a fungus *Fusarium oxysporum* making use of its enzyme lactonase. The enzyme stereoselectively hydrolyzes only (R)-pantolactone into (R)-pantoic acid, which is easily separated from intact (L)-pantolactone; (R)-pantoic acid is lactonized to (R)-pantolactone and further converted into (R)-pantothenic acid, and (L)-pantolactone can be recycled via racemization [170,671,672,732,733,734]. For the industrial synthesis of β-alanine, two processes utilizing acrylonitrile, ammonia, and sodium hydroxide or acrylic acid and ammonia as starting materials are mainly used [671]; biotechnological methods (biotransformation and fermentation) as an alternative way for production of β-alanine have recently drawn research attention and gained yields that are in reach of the industrial requirements [735,736,737].

Pantothenyl alcohol (panthenol), which itself has no vitamin activity but is quantitatively converted to pantothenic acid in animal and human body [672], is manufactured by chemical synthesis using (R)-pantolactone and 3-amino-1-propanol as starting materials [671,738]. Significant efforts have been devoted to development of microbial fermentation for production of pantothenic acid [171,739,740]. The main advantage of that approach is the direct formation of the desired stereoisomeric form, (R)-pantothenic acid [672]. Promising achievements as for overproduction of pantothenic acid production were gained with genetically modified bacteria *Escherichia coli*, *Corynebacterium glutamicum*, and *Bacillus subtilis*, but no fermentation process has been yet industrialized [171,671,680,732,738,740,741,742,743,744,745,746,747,748]. Despite high productivity of reported fermentations, further work is required to come up with even more suitable strains, in which the metabolic flux is mainly piped toward pantothenic acid to ensure a sufficiently high product yield on the consumed carbon source; only so the fermentation route can become economically competitive to currently used methods for pantothenic acid manufacturing [738].

Regarding fortification of foods using pantothenic acid, adult human intake of that vitamin has generally been considered adequate in view of the absence of deficiency in normal populations and the fact that the daily requirement for pantothenic acid is easily fulfilled from most natural dietary sources owing to its ubiquitous distribution [99,749]. Pantothenic acid (as calcium pantothenate or sodium pantothenate or dexpanthenol) is added to various foods (such as milk-based products, breakfast cereals, and rice powders) to prevent deficiency due to incorrect nutrition or malnutrition or for certain nutritional requirements (baby foods, e.g., for non-breastfed infants; athletes’ products; low-calorie, reduced-calorie, and vitamin-rich foods) [174,337,671,694,749,750,751,752,753]. Concerning biofortification of crops for pantothenic acid, no extensive research has been conducted in exploring possibilities of enhancing pantothenate levels in plants through breeding or genetic engineering [61,679,680,754].

### 5.3. Physiological Function of Pantothenic Acid

**Figure 14:**
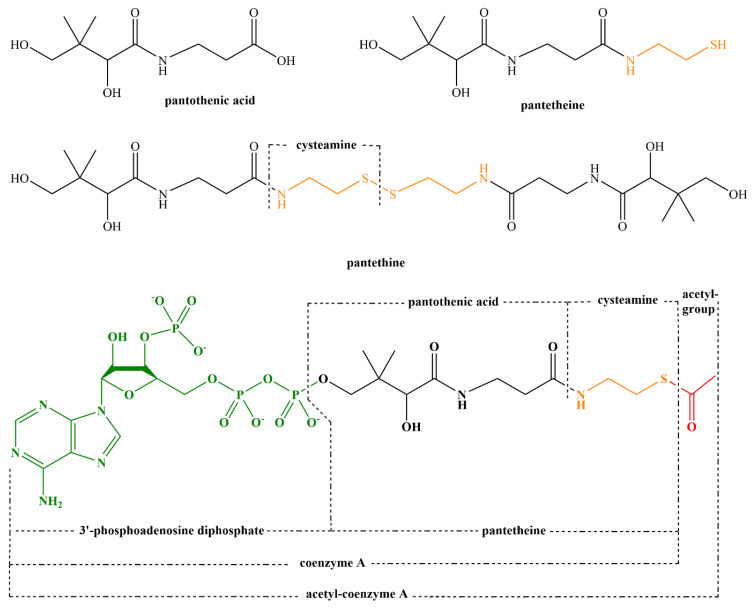
Structure of pantothenic acid, pantetheine, pantethine, and acetyl-CoA.

The vitamin B_5_ is essential for synthesis of coenzyme A (CoA) and acyl-carrier protein (ACP) in both yeast and mammalian cells [755]. CoA plays a vital role in many catabolic and anabolic reactions. It is necessary for synthesis of fatty acids, cholesterol, acetylcholine, bile acids, and others. It also plays a role in regulation of metabolism and gene expression. CoA is required for processing large organic molecules, such as lipids, carbohydrates, and proteins. These reactions generate energy with formation of acylated forms of CoA, such as acetyl-CoA, succinyl-CoA, propionyl-CoA, isovaleryl-CoA, isobutyryl-CoA, α-methylbutyryl-CoA, and fatty acyl-CoA [756]. The structure of pantothenic acid and its derivatives is shown in Figure 14.

ACP is important for synthesis of fatty acids. It is expressed in the inactive form, apo-ACP. Its activation to holo-ACP requires the attachment of a prosthetic group (the 4′-phosphopantetheinyl moiety). This happens during the reaction with CoA catalyzed by 4′-phosphopantetheinyl transferase [757]. Average daily recommended intakes of pantothenic acid are listed below in Supplementary Data Table S3 [412].

### 5.4. Pharmacokinetics of Pantothenic Acid

#### 5.4.1. Absorption

Vitamin B_5_ occurs in the diet mainly in form of its derivatives 4′-phosphopantetheine and CoA. In both cases, they must undergo a series of hydrolytic reactions to be converted to pantetheine or pantothenic acid prior the absorption (Supplementary Data Figure S1).

The first step in conversion of CoA is dephosphorylation to dephospho-CoA by alkaline phosphatase present in the intestinal lumen, followed by hydrolysis of the 5′-phosphodiester bonds in nucleotides of CoA or dephospho-CoA, to give a rise to phosphopantetheine. This reaction is catalyzed by ectonucleotide pyrophosphatase/phosphodiesterase (ENPP) [756,758]. There are 7 known isoforms of ENPPs. ENPPs 1–5 can hydrolyze diphosphate bond, and ENPP 2 is a secreted form, which preferably hydrolyzes phosphodiester bond in phospholipids. All other forms are transmembrane proteins. ENPPs 1 and 3 are soluble isoforms which have been detected in almost all tissues but are usually associated with specific cell types [758,759]. The next step is hydrolysis of phosphopantetheine to pantetheine. This reaction is again catalyzed by alkaline phosphatase. This final transformation step of pantetheine into pantothenate and cysteamine is under control of substrate specific pantotheinase presented in the lumen and intestinal tissue [756,758]. This enzyme breaks downs a single amide bond in pantetheine. There are known three human pantotheinase isoforms, called vascular non-inflammatory molecules, or, in short, vanins (VNN1-3) [760]. The VNN1 isoform is present on the apical side of enterocytes, and the same type is also present in other tissues with high CoA turnover, such as the hepatocytes and brush borders of the proximal tubule of the nephron in the kidney, where it helps with salvaging and recycling of pantothenic acid [761].

The conversion of CoA in serum is similar to conversion in the intestinal lumen; however, CoA in serum could be hydrolyzed directly to phosphopantetheine by ENPP1 or ENPP3 prior to previous dephosphorylation [756].

**Figure 15:**
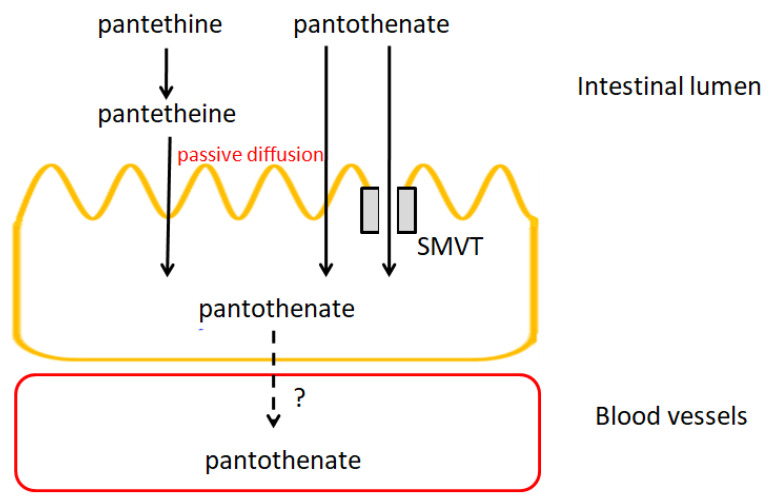
Membrane transport in the small intestine. SMVT, sodium-dependent multivitamin transporter.

An earlier rat study reported that uptake of pantothenate and pantetheine takes place in the small intestine via passive diffusion, which is mainly possible in higher vitamin concentrations [760,762]. Later studies with lower concentrations of pantothenic acid, however, have shown that the absorption occurs by a saturable sodium-dependent multivitamin transporter (SMVT) [701,763] (Figure 15). This transport system belongs to the Na^+^-dependent glucose transporter family, and it also carries biotin, its certain analogues, and lipoate. The transporter primarily interacts with a long side-chain side of molecules containing the carboxyl group, and such moiety is present in all mentioned substances. The transport is electrogenic with Na^+^: pantothenate coupling ratio 2:1. Na^+^ is crucial, and it cannot be replaced by other cations. The affinity constant for pantothenate transport is ~2 µM [763].

Similarly, as in the small intestine, pantothenic acid is absorbed in the colon via the same transporter SMVT. There are, however, no data regarding the mechanism of pantothenic acid transport from the enteric cells via the intestinal basolateral membrane into the bloodstream [701].

Shibata et al. (1983) detected only a 5.4 ± 3.5% of dose in the intestinal lumen and 10.1 ± 4.1% in the intestinal tissue 5 h after administration of the radiolabeled ^14^C-pantothenate into the intestinal lumen. Hence, most of the pantothenic acid was absorbed and distributed [760].

The pantethine (disulfide derivative of pantothenic acid and stable form of pantetheine) is hydrolyzed to pantetheine and, subsequently, to pantothenate during passing the intestinal wall to a considerable extent (80%) [764,765]. Indeed, pantothenate, but not pantethine (below detectable levels < 5–10%), was reported in plasma of patients suffering from cystinosis [765]. At least according to 24-h urine rate excretion of pantothenic acid, pantethine had about 1.5 higher rate of absorption compared to calcium pantothenate in rats [764].

#### 5.4.2. Distribution

Pantothenate is transported in blood in the free form, and it does not bind to albumin [766]. It is rapidly taken up by tissues and red blood cells. In fact, its levels in red blood cells are higher than in plasma/serum [767]. The transport into red blood cells is mediated by passive diffusion. Both the uptake and efflux are non-saturable and are not affected by presence of different Na^+^ and glucose concentrations, nor pH. Erythrocytes have only a limited ability to metabolize pantothenate; therefore, it is probable that they are used only as a transporter of pantothenate to other tissues. In red blood cells, pantothenic acid could be converted to 4′-phosphopantothenic acid, but not further to CoA [768]. Therefore pantothenic acid is transported in the form of pantothenate, 4′-phosphopantothenic acid and pantetheine in the red blood cells [760,768]. The uptake into different tissue, e.g., liver, lungs, kidney, heart, adipose tissue, and placenta, is via SMVT [769,770]. This transporter also enables the transport through the blood brain barrier [771].

In addition,, distribution studies in rats, where radiolabeled pantothenate was administered directly into the intestinal lumen, confirmed that vitamin B_5_ is concentrated in tissues, and only a low dose remains in the blood. The highest quantity of the dose was detected in muscles (34.7 ± 2.5% of dose) with lower amounts also in the liver (12.1 ± 2.2%), the kidney (5.2 ± 0.7%), and the colon (4.2 ± 0.3%). The amount in other organs (the heart, stomach, testis, brain, and lung) was around or below 1% [760].

#### 5.4.3. Metabolism

**Figure 16:**
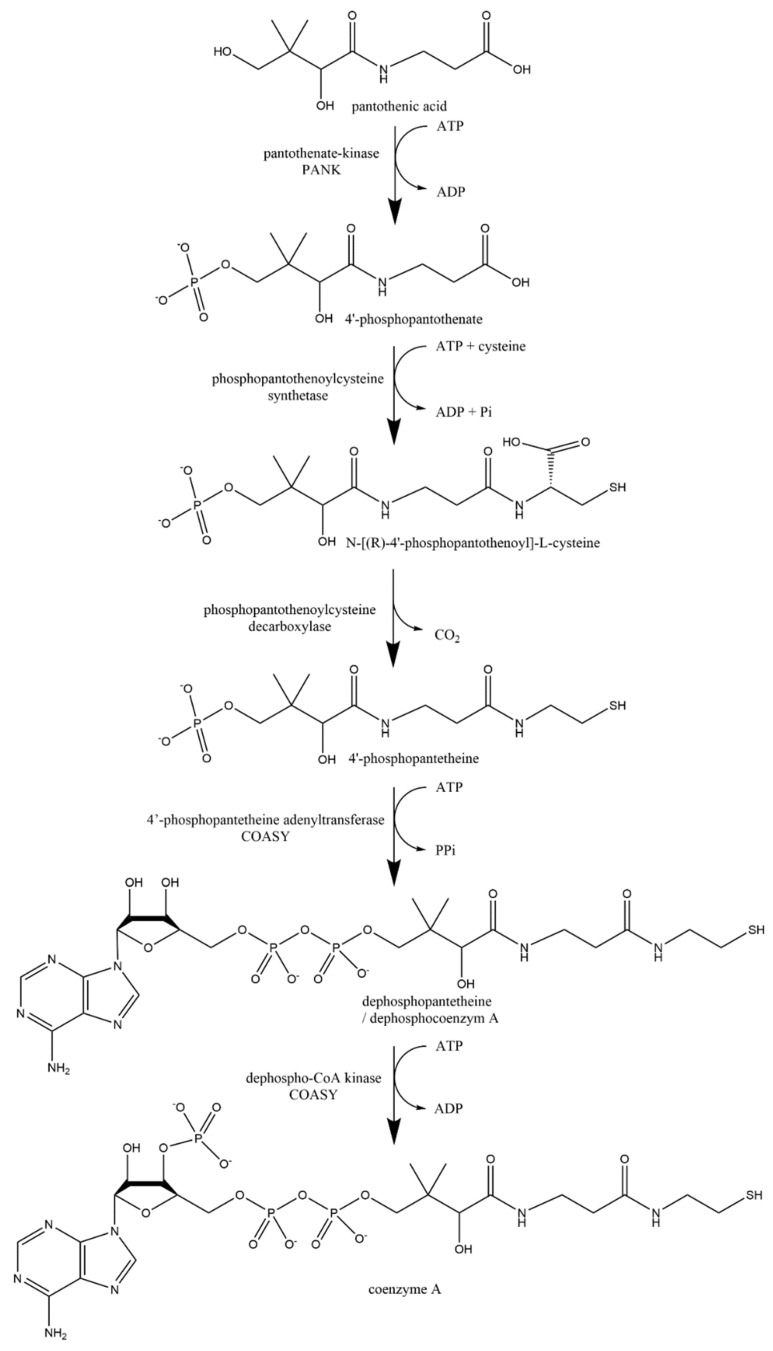
CoA biosynthesis. The five-step pathway starts with phosphorylation of pantothenic acid to 4′-phosphopantothenate by pantothenate-kinase (PANK). Then, 4′-phosphopantothenate condensates with cysteine. This reaction is catalyzed by phosphopantothenoylcysteine synthetase and gives rise to N-[(R)-4’-phosphopantothenoyl]-L-cysteine. This product is subsequently decarboxylated in presence of phosphopantothenoylcysteine decarboxylase to generate phosphopantetheine. Last two steps consist of phosphorylation of 4′-phosphopantetheine to form dephosphopantetheine and phosphorylation of 3′-hydroxy group of ribose to form CoA. Both reactions are catalyzed by an enzyme called COASY, which exhibit two enzymatic activities: 4′-phosphopantetheine adenyltransferase (catalyzes the fourth reaction) and dephospho-CoA kinase (catalyzes the fifth reaction).

The homeostasis of vitamin B_5_ is assured with 3 known sources: diet, microbial production, and degradation of endogenous CoA. The intracellular conversion of pantothenic acid to CoA is shown in Figure 16.

#### 5.4.4. Excretion

Pantothenic acid is mainly excreted via urine. Filtered pantothenic acid is reabsorbed through the brush border in proximal tubules via SMVT at physiological concentrations. This was proven in experiments with rat kidney membrane vesicles incubated with pantothenic acid in presence of NaCl in different concentration gradients. The transport via SMVT in the kidney is saturable (K_m_ 7.30 µM and V_max_ 34.8 pmol/mg protein per min) and could be inhibited by structurally similar compounds (4′-phosphopantothenate and 4′-phosphopantetheine) [766].

At higher concentrations, pantothenic acid undergoes tubular secretion. The secretory mechanism is the same as for penicillin, which can inhibit this process [766]. Logically, probenecid is also blocking this process [772].

Perfusion rat kidney studies found that panthothenic acid is metabolized in the kidney tissue to CoA (4.9 ± 0.9%), dephospho-CoA (6.3 ± 3.1%), pantetheine (8.0 ± 1.2%), and 4′-phosphopantetheine (9.5 ± 2.9%), but only the pure acid is excreted in the urine [766]. The excretion of pantothenic acid in urine was relatively slow in humans after i.m. application of 20 mg of calcium pantothenate since only 21.3 ± 1.5% was eliminated after 6 h [773]. The degree of urinary excretion of vitamin B_5_ is apparently dependent on the amount of the vitamin in the diet. Its excretion was relatively high, ranging from 60 to 72%, suggesting that its content in the diet is mostly sufficient [767,774,775].

### 5.5. Pantothenic Acid Deficiency

Vitamin B_5_ deficiency is generally rare since the vitamin is present in various foods, but it could be observed in people and animals with severe malnutrition. However, due to multi-nutrient deficiency, it is quite challenging to identify manifestations of deficiency specific for vitamin B_5_. For this reason, some experimental vitamin B_5_ deficiency studies were performed: Human subjects or animals received a diet devoid of pantothenic acid, or their diet was supplemented with a vitamin antagonist, ω-methylpantothenic acid (a pantothenate kinase inhibitor), or a combination of these approaches was employed [776,777,778,779,780]. Analytical methods for detection of vitamin B_5_ are summarized in Table 3 and in Supplementary Data Table S1.

#### 5.5.1. Symptoms of Vitamin B_5_ Deficiency in Animals

The most common symptoms of pantothenic acid deficiency in animals are growth problems, skin rash, gastrointestinal and nervous symptoms, such as ataxia, loss of coordination, and muscle weakness. Similar symptoms appeared also in human studies. Symptoms are described in more detail in Table 11.

Pantothenic acid deficiency in rats can cause breeding problems and failure of embryo implementation with subsequent resorption [781]. The deficiency throughout pregnancy has an impact on endocrine function of the placenta, which is linked to a lower production of progesterone and acetylcholine, and underdevelopment of fetuses [782]. Among the reported abnormalities belong: cerebral and eye defects, digital hemorrhages and edema, interventricular septal defects, anomalies of the aortic arch pattern, hydronephrosis and hydroureter, clubfoot, tail defects, cleft palate, and dermal defects [781].

The impact of pantothenic acid-free diet on kinetic of CoA is of interest. As expected, the deficient diet results in marked drops of CoA content in the rat liver, adrenal gland, kidney, and heart. The decrease was, however, immediate only in the adrenal gland, while there was a 3-week lag period for depletion in the other tissue. The drop was gradual but not excessive, since 35–40% of normal content remained in tissues during another 6 weeks. In ducklings, the first signs of deficiency were observed after 10–15 days. The level of CoA in the liver and the heart were measured from 5th day of experiment when it rapidly decreased to 40% of the normal values. The liver of both experimental animals showed decreased ability to utilize pyruvate [783]. Data on liver steatosis are ambiguous. Pantothenic acid-deficient dogs developed fatty livers, while fatty liver was not observed in pantothenic acid deficient rats, even those fed a high-cholesterol diet (1% cholesterol) [784,785]. It should be, however, be mentioned that these rats were resistant to fat liver deposition and the level of serum and liver cholesterol was only slightly elevated compared to controls [785].

#### 5.5.2. The Symptoms of Vitamin B_5_ Deficiency in Human Subjects

Humans administered with a vitamin B_5_ antagonist ω-methyl pantothenic acid developed personality changes with irritability, restlessness, and quarrelsomeness. Similar symptoms developed in humans on a diet deficient in vitamin B_5_ content (8 weeks) [776]. An analogous experiment was performed, as well, by Fry et al., in 1976, who tested the effect of a diet essentially free from pantothenic acid on human health. In that study, however, no clinical symptoms of deficiency were observed, but some subjects appeared listless and complained of fatigue at the end of diet deficient period (63 days) [786].

Lower levels of pantothenic acid were also detected in some brain regions affected by Alzheimer’s disease compared with controls. It is still unknown whether vitamin B_5_ depletion participates in the pathophysiology or if this is simply a consequence of the underlying neuropathological process [787].

#### 5.5.3. Mutation of Pantothenate Kinase 2

Symptoms of pantothenic acids shortage are also traceable in individuals with mutation of pantothenate kinase 2 [788]. Three active isoforms (PANK1α, PANK1β, PANK2, and PANK3) are known for pantothenate kinase (PANK), and they are located in different cellular compartments (mitochondria, cytosol, and nucleus). There is also one inactive form, PANK4, which has a weak phosphatase activity towards 4′-phosphopantetheine [756,758]. PANK is essential for phosphorylation of panthothenate (Figure 16) and subsequent formation of phosphopantothenate which condensates with cysteine in the next step in CoA biosynthesis. PANK2 isoform is the only one from PANKs located in the mitochondria. The mutation of PANK2 leads to pantothenate kinase-associated neurodegeneration. Phosphopantothenate is not produced, and cysteine is accumulated in globus pallidus where it binds iron, causing tissue damage by promoting oxidative stress, which results in neurodegeneration, and increases the risk of Parkinson’s disease and other oxidative stress disorders [788]. The pantothenate kinase-associated neurodegeneration has two forms, classic and atypical. The classic form is characterized by an early onset (before 6 years old) and rapid progression. Among the clinical features belong gait and postural disbalance, limb spasticity and spinal deformity, dystonia, choreoathetosis, retinitis pigmentosa, and bulbar dysfunction. Rarely described are also parkinsonism and neuropsychiatric features. First manifestation of an atypical type can appear at different ages, with a range from 1 to 28 years of life. Some signs overlap with the classic form, but the atypical form is less aggressive, and most individuals remain without serious motility derangement into adulthood [789,790,791].

### 5.6. Pharmacological Use of Pantothenic Acid

#### 5.6.1. Triacylglycerols, Cholesterol

The impact on lipid metabolism was studied in many clinical studies. Pantethine was used instead of pantothenic acid since its effect on inducing liver synthesis of CoA was higher in animal studies: Pantethine (0.1%) increased synthesis of CoA by 45% in rat liver homogenates, while pantothenic acid in an equivalent amount did not. Further, the level of FFA was decreased after the administration of 0.1% pantethine during 2 weeks in rats. Again, this effect was not observed when pantothenate was administered [804].

In clinical studies, the doses of pantethine reached 600 or 900 mg/day and were administered to patients suffering from different forms of dislipidemia; in some studies, patients with diabetes mellitus were also included. Most of these studies proved a lowering effect on triacylglycerols, LDL, and VLDL, while HDL level rose or was unchanged.

In patients with low to moderate cardiovascular risk, with a therapeutic lifestyle and change of diet enriched with pantethine during 16 weeks (600 mg/day from weeks 1–8 and 900 mg/day from weeks 9–16), a significant decrease in total cholesterol at 16 week and LDL cholesterol in 8–16 weeks compared to placebo was observed. The decreasing trend was also evident in non-high-density-lipoprotein cholesterol at weeks 8 and 12, which reached significance at 16 weeks. The level of CoQ10 significantly increased above the baseline in both groups, and homocysteine level did not change [805].

Similarly, in patients with mixed hyperlipidemia, who received 300 mg of pantethine 3 times daily during 8 weeks, a significant lowering of total and LDL cholesterol of 13.5% (for both) and a 10% rise in HDL cholesterol at the end of the treatment was observed. The level of blood triglycerides was reduced by 30% compared with placebo [806].

Decreases in cholesterolemia, triglyceridemia, LDL cholesterol, and apolipoproteins B and increases in HDL cholesterol and apolipoproteins A were also reported in a 3 month-study with hypercholesterolemic and/or hypertriglyceridemic patients, with or without diabetes mellitus, receiving 600 mg/day of pantethine orally, to compare with their basal values [807].

In addition, a post-registration surveillance study confirmed drops in LDL cholesterol and triglyceride levels with an increase in HDL cholesterol compared with basal values in hyperlipidemic and diabetes mellitus of II type patients. The effect in insulin-dependent diabetes mellitus (type I) patients was confirmed only for total cholesterol. In general, longer duration of treatment led to a more pronounced effect [808]. The progressive and significant reduction of triacylglycerols, VLDL, and LDL was also observed in study focused on treatment hyperlipidemia in type I and type II diabetic patients undergoing peritoneal dialysis at 2, 4, and 6 months compared with baseline values. The HDL cholesterol, however, did not change [809].

#### 5.6.2. Cystinosis

Pantethine is a source of cysteamine and, therefore, could be suggested in treatment of nephropathic cystinosis. Cysteamine effectively reduces cystine content in cystinotic cells by entering cystinotic lysosomes and reducing cystine to cysteine and cysteamine-cysteine mixed disulfide, which can freely leave lysosomes [765,810]. Leukocyte cystine depletion serves as an indicator for treatment of cystinosis. Cysteamine can deplete >90% of leukocyte cystine. In a study where pantethine was administered to four children with nephropatic cystinosis in dose of 70–1000 mg/kg orally, cystine was depleted from the leukocytes less effectively (no more than 80% was achieved). Due to this, pantethine could not be recommended for treatment of nephropathic cystinosis, but it could be used in case of cysteamine intolerance [765].

#### 5.6.3. Skin Disorders

##### Acne

The impact of orally administered pantothenic acid on mild and moderate acne vulgaris was tested in 10 men and women (average age of 31.8 ± 8.4). The pantothenic acid (2.2 g/day) was administered along with other vitamins (thiamine, riboflavin, niacin, pyridoxine, folic acid, cyanocobalamin, biotin, and L-carnitine). There was a significant reduction in lesion count from baseline 20.45 ± 10.44 to 11.18 ± 6.38 after eight weeks of treatment. Sixty percent of subjects reported a marked improvement, and 30% reported slight improvement, while one of the subjects reported no change [811]. The randomized, double blind placebo-controlled study proved the effectiveness of the same product on reduction of global facial lesions count [812].

##### Topical Treatment (Eyes, Nose Mucosa, Skin)

The reduced form of vitamin B_5_ dexpanthenol (D-panthenol) is mainly used for topical treatment of skin and mucous lesions. Its effects are, however, likely not related to the physiological function of vitamin B_5_ but are mediated by its moisturizing effect, which is based on its hygroscopic property. It could be used topically as a cream, emollient, drops, gel, lotion, oil, ointment, solution, and spray in concentration of 2–5% [813]. Dexpanthenol protects epithelium and promotes cellular proliferation. During the wound healing, it helps to recover the epidermal barrier function, has anti-inflammatory activity, and supports wound closure [814].

The healing properties of dexpanthenol-containing cream (5%) were confirmed on superficial skin lesions caused by application of 5% sodium lauryl sulfate solution for 4 h. One week, twice daily cream application led to a significant enhancement of stratum corneum hydration, as well as reduction in skin roughness and inflammation [815]. Other studies confirmed the effect of dexpanthenol containing emollient on sodium dodecyl sulfate (0.5%) induced skin barrier dysfunction. Dexpanthenol improved skin hydration and increased ceramide 3, as well as FFA and cholesterol content, in the stratum corneum, and it also supported recolonization of the skin with commensal bacteria [816].

Dexpanthenol in ointment with petroleum jelly led to a significantly faster and pronounced reduction of skin lesions size and better re-epithelialization of ablative CO_2_ laser photo-damaged skin than the petroleum-jelly cream itself [817]. Protective effect of an ointment with dexpanthenol (5%) was also seen in combination with zinc oxide in irritant diaper dermatitis in comparison to the control ointment base [818]. Two-week administration of dexpanthenol 5% water-oil formulations 4–8 time daily restored the skin barrier of freshly tattooed skin. The disadvantage of this study is that the effect was not compared with a control group [819].

In treatment of atopic dermatitis in children, dexpanthenol (5%) ointment exerted equal effectiveness to hydrocortisone (1%) ointment and, therefore, can be used as alternative to treatment of mild and moderate atopic dermatitis [820].

Use of dexpanthenol cream (5%) on treatment of traumatic nipples of breastfeeding mothers had the same therapeutic effect in comparison with pure lanolin or 0.2% peppermint oil creams administered every 8 h for 14 days [821].

The application of 2% dexpanthenol drops on corneal epithelial wounds after surface laser ablation only induced little effect on corneal epithelial regeneration, and, in general, the effect was of minimal clinical relevance after 2 months of use [822]. However, dexpanthenol has been found to be effective in treatment of dry eye, where it exerted superior improvement in disturbances of corneal epithelium permeability comparing with dexpanthenol-free drops [823].

Dexpanthenol is also added to topical nasal decongestant (sprays and droplets) containing α-sympathomimetics to treat acute allergic or non-allergic rhinitis or after nasal surgery. A combined preparation of oxymethazoline (0.05%) with dexpanthenol (5%) showed a better efficacy than xylomethazoline (0.1%) alone in patients with acute allergic rhinitis or with post-nasal surgery. The relief in nasal congestion was significantly better, recovery time was shorter, and significant improvements in sneezing, nasal discharge, and irritation were also observed [824]. Similarly, addition of dexpanthenol to xylometazoline significantly reduced nasal obstruction, rhinorrhea, hyperplasia of nasal concha, and redness of the nasal mucous membrane compared with xylometazoline alone [825,826].

### 5.7. Toxicity of Pantothenic Acid

#### 5.7.1. Acute Toxicity

Vitamin B_5_ apparently has a very low toxicity. Oral administration of 1 g/kg of calcium pantothenate to dogs and monkeys was not associated with any symptom of toxicity, which was confirmed by the absence of any pathological changes in examinated organs. Indeed, LD_50_ in mice and rats following oral, subcutaneous, intraperitoneal, and intravenous application were high. The LD_50_ for mice was 10 g/kg (oral), 2.7 g/kg (s.c.), 0.92 g/kg (i.p.), and 0.91 g/kg (i.v.). The rats survived the orally administered dose of 10 g/kg without showing any toxic symptoms, and the others’ respective LD_50_s were 3.5 g/kg (s.c.), 0.82 g/kg (i.p.), and 0.83 g/kg (i.v.). The lethal dose caused respiratory failure during two hours following i.v. and i.p. administration and during 6–12 h after oral and s.c. application [827].

#### 5.7.2. Chronic Toxicity

Similarly, chronic toxicity of vitamin B_5_ is very low; hence, this vitamin is generally considered safe. No symptoms of chronic toxicity nor pathological organ changes were detected in rats fed by 50 or 200 mg/daily of calcium pantothenate during 190 days nor in dogs (daily dose 50 mg/kg) and in monkeys (1 g daily for 4–5 kg body weight) fed for 6 months [827].

No tolerable upper intake limit for pantothenic acid has been established due to a lack of toxicity reports. Only massive doses of calcium pantothenate (10–20 g/day) can cause mild diarrhea in humans [828]. Pantethine is also well tolerated. In some studies, no side effects were reported at all [807]. Few patients treated with high doses of pantethine (900 mg/day) complained to mild gastric discomfort and pruritus [808,809]. There is one case report of eosinophilic pleurocardial effusion in a 76-year-old woman, who consumed vitamin B_5_ (300 mg/day) and vitamin H (10 mg/day) for two months. She was hospitalized with chest pain and breathing problems. Blood tests showed an inflammatory syndrome with a high eosinophil concentration (1200–1500 cells/mm^3^) [829].

## 6. Conclusions

This article summarized the information of 4 vitamins of the B-complex. These vitamins have many similar properties, ranging from their water solubility associated with significant losses when boiling their food sources through the need of specific transporters, their storage in erythrocytes, and urinary excretion, to their safety (Table 12). Concerning the last, the only exception is one form of vitamin B_3_, nicotinic acid, which can cause several side effects but can be, on the other hand, administered as a suitable drug for the treatment of hyperlipidemia. Contrarily, the use of other, here described, vitamins is unambiguously beneficial only in cases of hypovitaminosis or specific states associated with functional lack of the vitamin. Due to their low, or even non-existent, toxicity, however, they have been investigated for many possible indications.

## Figures and Tables

**Table 1:** Thiamine contents in selected foodstuffs.

Food	Thiamine Content (μg/100 g)	References
Oats	520–763	[11,90]
Wheat	276–525	[58,90,91]
Rice, brown	300–413	[11,22,59,92]
Rice, white	50–80	[11,22,59,92]
Maize	246–385	[11,90,92]
Rye	316–350	[11,90]
Barley	191–399	[11,56,91]
Millet	358–421	[11,93,94]
Sorghum	277–380	[93,94,95]
Soybean	874–1300	[11,20,59]
Lentil	433–873	[11,96,97]
Peanut	600	[20]
Macadamia nut	365–1195	[11,21]
Pistachio nut	654–870	[11,20,21]
Hazelnut	317–643	[11,21]
Walnut	227–340	[20,21]
Almond	192–210	[11,20,21]
Garlic	200	[11]
Potato	80–170	[11,59,92]
Carrot	66–130	[11,98]
Cabbage	61–230	[11,98]
Tomato	37–50	[11,98]
Broccoli	71–150	[11,98]
Cauliflower	60	[98]
Spinach	78–90	[11,98]
Orange	87	[11]
Avocado	67	[11]
Strawberry	20–24	[11,98]
Apple	17–40	[11,98]
White bread	100	[99]
Brown bread	210	[99]
Pork	600–950	[59,78,100]
Beef	50–160	[78,100]
Chicken breast	40–170	[78,100]
Liver, beef	189	[101]
Liver, pork	283	[101]
Tuna	130	[102]
Sardines	10	[102]
Baker’s yeasts	1880	[101]
Oyster mushroom	50–150	[103,104]
Button mushroom	70–94	[103,105]
Milk	30–70	[11,59,92,106]
Yogurt	50–60	[106]
Cheese, cheddar	29	[101]
Eggs	40–80	[92,107]

**Table 2:** Genetic defects in thiamine transport and metabolism.

Deficiency	Gene Mutation	Disease	Symptoms
THTR1	SLC19A2	Thiamine-responsive megaloblastic anemia (TRMA, also known as the Rogger syndrome)	Megaloblastic anemia, diabetes mellitus, hearing loss
THTR2	SLC19A3	Biotin-thiamine responsive encephalopathy	Episodic encephalopathy, gait ataxia, seizures, bulbar dysfunction
Mitochondrial TPP transporter	SLC25A19	Amish lethal microcephaly	Severe congenital microencephaly, death within the first year, episodic encephalopathy
TPK1	TPK1 gene	Thiamine metabolism dysfunction syndrome 5	Episodic encephalopathy, ataxia, dystonia and spasticity, loss of ability to walk
BCKDH	DBT gene	Maple syrup urine disease (MSUD)	Impaired mental development, seizures, lethargy, progressive neurodegeneration, maple syrup odor in the cerumen and the urine

**Table 3:** Summary of methods for determination of vitamin B_1–5_ in human biological materials.

Technique	Sensitivity nmol/L	Analytes	Matrix	Advantages	Disadvantages	Refs.	Publication Year
LC-MS	LOQ-LLOQ 0.15–246.32 (B_1_–B_5_)	B_1_, B_1_-TMP, B_1_-TPP, B_2_, B_2_-FAD, B_3_-NAM, B_5_	human milk serum whole blood dry blood (VAMS) plasma tears urine	* short analysis time * small sample volume (50–250 µL) * specificity (MRM) * simple sample preparation * sensitivity	* SIM in some methods * complicated sample preparation (breast milk) * complicated gradient elution in some methods	[227,228,229,230,231,232,233,234,235,236,237,238,239]	2011–2020
HPLC-FLD	LOQ-LLOQ 0.5–23.51 (B_1_-TPP)	B_1_, B_1_-TMP, B_1_-TPP, B_2_, B_2_-FAD, B_2_-FMN	whole blood dry blood spot plasma h uman milk	* sample volume (100–250 µL) * sensitivity	* complicated sample preparation * derivatization * long analysis time	[240,241,242,243,244,245]	2011–2020
HPLC-PDA	LOD 212.56–4.09 × 10^3^ (B_2_–B_3_-NAM)	B_1_, B_2_, B_3_-NAM	plasma urine	* simple sample preparation	* long analysis time * poor sensitivity	[246,247]	2009–2014
Sensors/nanodots/ CL/FLD/ECD	LOD 6.8 × 10^−6^–0.25 × 10^3^ B_1_	B_1_, B_2_	urine serum plasma	* simple sample preparation * cheap	* research only—not commercially available * indirect detection	[248,249,250,251,252]	2002–2020
Microbiological test kits	LLOQ 64.98–83.93 (B_3_–B_5_)	B_3_, B_5_,	serum	* small sample volume (50–100 µL)	* high price (working in duplicate recommended) * long analysis time (24 h)	[253,254]	2021
HPLC-FLD kits	LOD 1.18–12.71 (B_1_-TPP–B_2_-FAD)	B_1_, B_1_-TPP, B_1_-TMP, B_2_, B_2_-FAD, B_2_-FMN	plasma whole blood	* small sample volume (50–300 µL) * sensitivity	* long analysis time * different extraction procedures for each vitamin * high price for small sample series	[255,256,257,258]	2021
ELISA kits	LOD 0.93 × 10^−3^–6.93 (B_1_–B_2_)	B_1_, B_2_	serum plasma cell culture supernatant tissue breast milk sperm urine	* small sample volume (40–250 µL) * one kit for various matrices * sensitivity	* for research only * time and money consuming for small sample series	[259,260,261]	2021

LOD, Limit of Detection; LOQ, Limit of Quantification; LLOQ, Lower Limit of Quantification; B_1_, thiamine; B_1_-TPP, thiamine pyrophosphate/diphosphate; B_1_-TMP, thiamine monophosphate; B_2_, riboflavin; B_2_-FAD, flavin adenine dinucleotide; B_2_-FMN, flavin adenine mononucleotide; B_3_, niacin; B_3_-NAM, nicotinamide; B_5_, pantothenic acid; CL, Chemiluminescence; ECD, Electrochemical Detection; ELISA, Enzyme-Linked ImmunoSorbent Assay; FLD, Fluorescence Detection; HPLC, High Performance Liquid Chromatography; LC-MS, Coupling of Liquid Chromatography and Mass Spectrometry; MRM, Multiple Reaction Monitoring; PDA, PhotoDiode Array Detection; SIM, Selected Ion Monitoring; VAMS, Volumetric Absorptive MicroSampling.

**Table 4:** Recommended dietary allowances for thiamine.

Individuals	Condition, Age	Dose (mg/kg)
Adults male		1.2
Adults female		1.1
	pregnancy	1.4
	lactation	1.4
Children	0–6 month	0.2
	7–12 month	0.3
	1–3 years	0.5
	4–8 years	0.6
	9–13 years	0.9
Adolescent male	14–18 years	1.2
Adolescent female	14–18 years	1.0

**Table 5:** Riboflavin contents in selected foodstuffs.

Food	Riboflavin Content (μg/100 g)	References
Oat	139	[11]
Wheat	57–265	[56,58,309]
Rice, brown	40–140	[11,61]
Rice, white	20–60	[11,61]
Maize	80–201	[11,92]
Rye	200–251	[11,56]
Barley	100–114	[11,56]
Millet	210–290	[11,93]
Sorghum	50–150	[93,94,95]
Soybean	870	[11,20]
Lentil	61–211	[11,113,310]
Peanut	100	[20]
Macadamia nut	162–367	[11,21]
Pistachio nut	160–447	[11,20,21]
Hazelnut	113–370	[11,21]
Walnut	150–395	[20,21]
Almond	1138–1432	[11,20,21]
Garlic	110	[11]
Potato	32–36	[11,92,111]
Carrot	10–58	[11,98]
Cabbage	20–40	[11,98]
Tomato	20–10月	[11,98]
Broccoli	117–120	[11,98]
Spinach	180–189	[11,98]
Cauliflower	90	[98]
Orange	40	[11]
Avocado	130	[11]
Strawberry	20–22	[11,98]
Apple	26–40	[11,98]
White bread	110	[99]
Brown bread	160–322	[99,309]
Pork	100–309	[78,92,100]
Beef	90–170	[78,92,100]
Chicken breast	30–120	[78,92,100]
Liver, beef	2760	[101]
Liver, pork	3000	[101]
Tuna	70	[102]
Sardines	340	[102]
Oyster mushroom	200–210	[103,104]
Button mushroom	384–390	[103,105]
Baker´s yeast	1113	[101]
Milk	169–180	[11,92,311,312]
Yogurt	160–270	[106,311]
Cheese, cheddar	441	[101]
Eggs	457–500	[92,107]

**Table 6:** Tissue localization of riboflavin transporters in humans.

Transporter	Localization	Mechanism
SLC52A1	Placenta, small intestine	Na^+^, Cl^–^, pH-independent
SLC52A2	Ubiquitous, higher in brain and salivary gland	Na^+^, Cl^–^, pH-independent
SLC52A3	Testis, small intestine	Na^+^, Cl^–^ independent, pH sensitive with about ½ half of the activity at neutral pH and 1/3 at pH 8.5 when compared to pH 5.5

**Table 7:** Concentrations of B_2_ vitamers in plasma and erythrocytes.

Vitamer	Median Plasma Concentration (nmol/L)	Median Erythrocyte Concentration (nmol/L)
Riboflavin	10.5	negligible
FMN	6.6	44
FAD	74	469

Data are from Reference [356].

**Table 8:** Niacin contents in selected foodstuffs.

Food	Niacin Content (μg/100 g)	References
Oat	961–2370	[11,90,339,466]
Wheat	4957–5700	[56,90,92,93,339,466]
Rice, brown	3500–5433	[11,61,92,93,109,339,467]
Rice, white	1300–2400	[11,61,92,109,466,467]
Maize	1900–3630	[11,92,93,339]
Rye	1700–4270	[11,56,90,339,466]
Barley	4523–5200	[11,56,339,466]
Millet	4500–4720	[11,93,339]
Sorghum	2920–4880	[93,94,339,468]
Soybean	1623	[11,20]
Lentil	1930–2605	[11,96]
Peanut	12100	[20]
Macadamia nut	2473	[11]
Pistachio nut	1300	[11,20]
Hazelnut	1800	[11]
Walnut	570	[20]
Almond	3618	[11,20]
Garlic	700	[11]
Potato	1035–1573	[11,92,111]
Carrot	837–983	[11,448]
Cabbage	234–323	[11,448]
Tomato	400–683	[11,448]
Broccoli	639–814	[11,448]
Cauliflower	600	[98]
Spinach	724–1000	[11,448]
Orange	249–282	[11,448]
Avocado	1738	[11]
Strawberry	291–600	[11,448]
Apple	91–126	[11,448]
White bread	1600	[99]
Brown bread	3800	[99]
Pork	5600–5900	[78,92,100,469]
Beef	4600–6500	[78,92,100,466,469]
Chicken breast	6801–9181	[78,92,100,466]
Liver, pork	13,200	[101]
Liver, beef	15,300	[101]
Tuna	21,900	[102]
Sardines	10,100	[102]
Oyster mushroom	4952–5870	[103,104,308,470]
Button mushroom	2800–3300	[103,470]
Baker’s yeasts	12,300	[101]
Milk	89–130	[11,92,471]
Yogurt	90–200	[106,472]
Cheese, cheddar	52	[101]
Eggs	50–75	[92,107]

**Table 9:** Biochemical redox reactions utilizing NAD or NADP as cofactors.

NAD^+^/NADH	catabolism	NAD^+^ → NADH	Glycolysis	glucose → pyruvate
			Anaerobic glucose oxidation	Lactate ↔ pyruvate
			B-oxidation of fatty acids	palmitoyl CoA → acetyl CoA
			Amino acid catabolism	amino acid → acetyl/palmitoyl CoA
			The Krebs cycle	acetyl CoA/pyruvate → energy
	anabolism	NADH → NAD^+^	Glyconeogenesis	lactate/pyruvate ↔ glycogen
			Fat synthesis	glucose → TAG
			Steroid synthesis	cholesterol → various steroids
		NAD^+^ → NADH	Cholesterol synthesis	acetyl CoA → cholesterol
NADPH/NADP^+^	anabolism	NADPH → NADP^+^	Fatty acid synthesis	acetyl CoA → palmitate
			Cholesterol synthesis	acetyl CoA → cholesterol
			Bile acid synthesis	cholesterol → cholate, chenodeoxycholate
			Steroid synthesis	cholesterol → various steroids

**Table 10:** Pantothenic acid contents in selected foodstuffs.

Food	Pantothenic Acid Content (μg/100 g)	References
Oat	800–1350	[339,671]
Wheat	950–1200	[339,671]
Rice, brown	660–1860	[61,99,109,339,467]
Rice, white	250–1080	[61,99,109,467,671]
Maize	420–650	[339,671,705]
Rye	1340–1460	[339,512]
Barley	280	[339]
Millet	850	[339]
Sorghum	1550–1630	[468]
Soybean	793–1431	[101,710]
Lentil	1030–1430	[710]
Peanut	1412–1767	[714,715]
Macadamia nut	800	[716]
Pistachio nut	470–500	[716,717]
Hazelnut	900	[716]
Walnut	470–600	[716,717]
Almond	300–471	[716,718,719,720,721]
Garlic	596	[101]
Potato	350–440	[98,671]
Carrot	270	[98]
Cabbage	210	[98]
Tomato	290–320	[98,671]
Broccoli	610–1300	[98,671]
Cauliflower	1010–1040	[98,671]
Spinach	280	[98]
Orange	240–370	[671,699]
Avocado	1390–1460	[101,717]
Strawberry	300–370	[98,671,699]
Apple	61–100	[98,101,671]
Pear	70	[699]
White bread	300–460	[99,699]
Brown bread	630–760	[99,699,705]
Pork	500–700	[101,671,722]
Beef	500–750	[101,671,722,723]
Chicken breast	870–1500	[101,724,725]
Liver, beef	7170–7900	[101,671]
Liver, pork	6650–6800	[101,671]
Tuna	230–500	[102,705]
Sardines	690–1090	[102,705]
Oyster mushroom	1300	[470]
Button mushroom	1360	[470]
Baker’s yeasts	4900	[101]
Milk	320–580	[106,471,671,699]
Yogurt	450–500	[106,699]
Cheese, cheddar	413–500	[101,699]
Eggs	1350–1600	[107,671]

**Table 11:** Symptoms of pantothenic acid deficiency.

	Symptoms	Sources
Rodents (rats, mice, guinea pigs)	Growth: retardation, decrease in weight	[778,779,783,792,793,794,795,796]
	Skin and mucosa: ruffing and discoloration of the fur, thinning of hair, alopecia, dryness of the skin with scaly desquamation, nasal discharge, watering of the eyes	
	Digestive track: diarrhea, duodenal changes (Lieberkühn crypts—enlargement, hyperplasia, increase in space between crypts, atrophy; villi diminution, epithelial changes to cuboid or flat, leading to ulcerations, perforation and chronic lesions), salivation	
	Nervous system: muscle weakness of the hind legs, convulsions, coma	
	Glands: adrenal lesions	
Birds (ducklings and chicks)	Growth: retardation, decrease in weight	[783,797,798,799]
	Skin: scaly dermatitis, skin lesions, scabs around beak and eyes, feather depigmentation, dermal edema	
	Nervous system: severe ataxia, tendency to fall and inability to rise and laying panting	
	Glands: lymphoid cell necrosis in the bursa of Fabricius and the thymus, and a lymphocytic paucity in the spleen	
Pigs	Growth: failure to gain in weight, loss of appetite	[800,801,802]
	Skin: loss of hair, roughness of the coat	
	Digestive track: diarrhea, severe colonic lesions	
	Nervous system: ataxia, lesions in sensory neurons, sudden lifting one of the limbs from the ground, unusual walk, inability to walk or stand	
	Respiratory system: cough and nasal secretion	
Dogs	Growth: retardation Nervous system: sudden weakness, coma, rapid respiratory and heart rate, convulsions, spasticity of the hind legs	[784,803]
	Digestive track: decreased appetite, gastrointestinal symptoms, gastritis or enteritis	
	Glands: fatty liver, mottled thymusis	
	Blood: blood level of glucose and chlorides were lower and non-protein nitrogen was elevated	
	Urinary system: hemorrhagic kidney degeneration	
Humans	Nervous system: headache, irritability, restlessness, quarrelsomeness, excessive fatigue, numbness, paresthesia, muscle cramps, faulty coordination associated with tremor and peculiar gait	[776]
	Digestive track: abdominal rumbling, diarrhea, epigastric burning, regurgitation	
	Glands: loss of eosinophilic response to adrenocorticotropic hormone, increased sensitivity to insulin	

**Table 12:** Selected common biological properties of described vitamins.

Vitamin	Transport	Blood Storage	Excretion	Toxicity/Adverse Effects
Thiamine	transporter mediated + passive diffusion	erythrocytes—as TPP	urinary	none/minor
Riboflavin	transporter mediated	erythrocytes—as FAD	urinary	none/minor
Niacin	transporter mediated + passive diffusion	erythrocytes	urinary	None/minor with exception of high doses of nicotinic acid but not of nicotinamide which can cause flushing, headache, lactic acidosis or hepatotoxicity
Pantothenic acid	transporter mediated + passive diffusion	erythrocytes	Urinary	none/minor

## Supplementary Materials

The following are available online at https://www.mdpi.com/article/10.3390/nu14030484/s1. Table S1: Detailed summary of methods for determination of vitamin B_1–5_ in human biological materials; Table S2: Brief overview of human flavoproteins; Table S3: Average recommended intake of pantothenic acid for different age groups; Figure S1: Hydrolytic reactions leading to release of vitamin B_5_.

## References

[1] Spedding S. (2013). Vitamins are more Funky than Casimir thought. Australas. Med. J..

[2] Tylicki A., Lotowski Z., Siemieniuk M., Ratkiewicz A. (2018). Thiamine and selected thiamine antivitamins-biological activity and methods of synthesis. Biosci. Rep..

[3] Goodman L.S., Brunton L.L., Chabner B., Knollmann B.R.C. (2011). Goodman & Gilman’s Pharmacological Basis of Therapeutics.

[4] Brown G. (2014). Defects of thiamine transport and metabolism. J. Inherit. Metab. Dis..

[5] Manzetti S., Zhang J., Van der Spoel D. (2014). Thiamin function, metabolism, uptake, and transport. Biochemistry.

[6] Lonsdale D. (2006). A review of the biochemistry, metabolism and clinical benefits of thiamin(e) and its derivatives. Evid. Based Complement. Altern. Med..

[7] Bettendorff L., Wirtzfeld B., Makarchikov A.F., Mazzucchelli G., Frederich M., Gigliobianco T., Gangolf M., De Pauw E., Angenot L., Wins P. (2007). Discovery of a natural thiamine adenine nucleotide. Nat. Chem. Biol..

[8] Jurgenson C.T., Begley T.P., Ealick S.E. (2009). The structural and biochemical foundations of thiamin biosynthesis. Annu. Rev. Biochem..

[9] Bocobza S.E., Aharoni A. (2008). Switching the light on plant riboswitches. Trends Plant Sci..

[10] Du Q., Wang H., Xie J. (2011). Thiamin (vitamin B1) biosynthesis and regulation: A rich source of antimicrobial drug targets?. Int. J. Biol. Sci..

[11] Wolak N., Zawrotniak M., Gogol M., Kozik A., Rapala-Kozik M. (2017). Vitamins B1, B2, B3 and B9-Occurrence, Biosynthesis Pathways and Functions in Human Nutrition. Mini Rev. Med. Chem..

[12] Fitzpatrick T.B., Chapman L.M. (2020). The importance of thiamine (vitamin B1) in plant health: From crop yield to biofortification. J. Biol. Chem..

[13] Ejsmond M.J., Blackburn N., Fridolfsson E., Haecky P., Andersson A., Casini M., Belgrano A., Hylander S. (2019). Modeling vitamin B1 transfer to consumers in the aquatic food web. Sci. Rep..

[14] Yoshii K., Hosomi K., Sawane K., Kunisawa J. (2019). Metabolism of Dietary and Microbial Vitamin B Family in the Regulation of Host Immunity. Front. Nutr..

[15] Fattal-Valevski A. (2011). Thiamine (Vitamin B1). J. Evid. Based Integr. Med..

[16] Turck D., Bresson J.L., Burlingame B., Dean T., Fairweather-Tait S., Heinonen M., Hirsch-Ernst K.I., Mangelsdorf I., McArdle H.J., Naska A. (2016). Dietary reference values for thiamin. EFSA J..

[17] Chawla J., Kvarnberg D. (2014). Hydrosoluble Vitamins.

[18] O’Connor A. (2012). An overview of the role of bread in the UK diet. Nutr. Bull..

[19] Lockyer S., Spiro A. (2020). The role of bread in the UK diet: An update. Nutr. Bull..

[20] Bonku R., Yu J.M. (2020). Health aspects of peanuts as an outcome of its chemical composition. Food Sci. Hum. Wellness.

[21] Stuetz W., Schlormann W., Glei M. (2017). B-vitamins, carotenoids and alpha-/gamma-tocopherol in raw and roasted nuts. Food Chem..

[22] Prinzo Z.W. (1999). Thiamine Deficiency and Its Prevention and Control in Major Emergencies.

[23] Whitfield K.C., Bourassa M.W., Adamolekun B., Bergeron G., Bettendorff L., Brown K.H., Cox L., Fattal-Valevski A., Fischer P.R., Frank E.L. (2018). Thiamine deficiency disorders: Diagnosis, prevalence, and a roadmap for global control programs. Ann. N. Y. Acad. Sci..

[24] Pacei F., Tesone A., Laudi N., Laudi E., Cretti A., Pnini S., Varesco F., Colombo C. (2020). The Relevance of Thiamine Evaluation in a Practical Setting. Nutrients.

[25] Panijpan B., Ratanaubolchai K. (1980). Kinetics of thiamine-polyphenol interactions and mechanism of thiamine disulphide formation. Int. J. Vitam. Nutr. Res..

[26] Dhir S., Tarasenko M., Napoli E., Giulivi C. (2019). Neurological, Psychiatric, and Biochemical Aspects of Thiamine Deficiency in Children and Adults. Front. Psychiatry.

[27] Hilker D.M., Somogyi J.C. (1982). Antithiamins of plant origin: Their chemical nature and mode of action. Ann. N. Y. Acad. Sci..

[28] Frank L.L. (2015). Thiamin in Clinical Practice. JPEN J. Parenter Enter. Nutr..

[29] Vimokesant S., Kunjara S., Rungruangsak K., Nakornchai S., Panijpan B. (1982). Beriberi caused by antithiamin factors in food and its prevention. Ann. N. Y. Acad. Sci..

[30] Fabre B., Geay B., Beaufils P. (1993). Thiaminase activity in Equisetum arvense and its extracts. Plant Méd. Phytothér..

[31] Yang P.F., Pratt D.E. (1984). Antithiamin Activity of Polyphenolic Antioxidants. J. Food Sci..

[32] Sannino D., Angert E.R. (2017). Genomic insights into the thiamin metabolism of *Paenibacillus thiaminolyticus* NRRL B-4156 and *P. apiarius* NRRL B-23460. Stand. Genom. Sci..

[33] Wang R.S., Kies C. (1991). Niacin, thiamin, iron and protein status of humans as affected by the consumption of tea (*Camellia sinensis*) infusions. Plant Foods Hum. Nutr..

[34] Nishimune T., Watanabe Y., Okazaki H., Akai H. (2000). Thiamin is decomposed due to Anaphe spp. entomophagy in seasonal ataxia patients in Nigeria. J. Nutr..

[35] Ringe H., Schuelke M., Weber S., Dorner B.G., Kirchner S., Dorner M.B. (2014). Infant botulism: Is there an association with thiamine deficiency?. Pediatrics.

[36] Taungbodhitham A.K. (1995). Thiamin Content and Activity of Antithiamin Factor in Vegetables of Southern Thailand. Food Chem..

[37] Somogyi J.C. (1971). On antithiamine factors of fern. J. Vitam..

[38] Murata K., Tanaka R., Yamaoka M. (1974). Reaction mechanisms of thiamine with thermostable factors. J. Nutr. Sci. Vitam..

[39] Rungruangsak K., Tosukhowong P., Panijpan B., Vimokesant S.L. (1977). Chemical interactions between thiamin and tannic acid. I. Kinetics, oxygen dependence and inhibition by ascorbic acid. Am. J. Clin. Nutr..

[40] Wills R.B.H., McBrien K.J. (1980). Antithiamin activity of tea fractions. Food Chem..

[41] Somogyi J.C., Bonicke R. (1970). Connection between chemical structure and antithiamine activity of various phenol derivatives. Bibl. Nutr. Dieta.

[42] Somogyi J.C., Nageli U. (1976). Antithiamine effect of coffee. Int. J. Vitam. Nutr. Res..

[43] Hilker D.M. (1968). Antithiamine factors in blueberries. Int. Z. Vitam..

[44] Schaller K., Holler H. (1976). Thiamine absorption in the rat. IV. Effects of caffeic acid (3,4-dihydroxycinnamic acid) upon absorption and active transport of thiamine. Int. J. Vitam. Nutr. Res..

[45] Beruter J., Somogyi J.C. (1967). 3,4-Dihydroxycinnamic acid, an antithiamine factor of fern. Experientia.

[46] Horman I., Brambilla E., Stalder R. (1981). Evidence against the reported antithiamine effect of caffeic and chlorogenic acids. Int. J. Vitam. Nutr. Res..

[47] Zhang F., Masania J., Anwar A., Xue M., Zehnder D., Kanji H., Rabbani N., Thornalley P.J. (2016). The uremic toxin oxythiamine causes functional thiamine deficiency in end-stage renal disease by inhibiting transketolase activity. Kidney Int..

[48] Burns A., Gleadow R., Cliff J., Zacarias A., Cavagnaro T. (2010). Cassava: The Drought, War and Famine Crop in a Changing World. Sustainability.

[49] Leichter J., Joslyn M.A. (1969). Kinetics of thiamin cleavage by sulphite. Biochem. J..

[50] Vanier N.L., Paraginski R.T., Berrios J.D., Oliveira L.D., Elias M.C. (2015). Thiamine content and technological quality properties of parboiled rice treated with sodium bisulfite: Benefits and food safety risk. J. Food Compos. Anal..

[51] Ottaway P.B. (2010). Stability of Vitamins during Food Processing and Storage.

[52] Yagi N., Itokawa Y. (1979). Cleavage of thiamine by chlorine in tap water. J. Nutr. Sci. Vitam..

[53] Kimura M., Itokawa Y., Fujiwara M. (1990). Cooking losses of thiamin in food and its nutritional significance. J. Nutr. Sci. Vitam..

[54] Dwivedi B.K., Arnold R.G. (1973). Chemistry of thiamine degradation in food products and model systems: A review. J. Agric. Food Chem..

[55] Kaplan Evlice A., Özkaya H. (2020). Effects of wheat cultivar, cooking method, and bulgur type on nutritional quality characteristics of bulgur. J. Cereal Sci..

[56] Calinoiu L.F., Vodnar D.C. (2018). Whole Grains and Phenolic Acids: A Review on Bioactivity, Functionality, Health Benefits and Bioavailability. Nutrients.

[57] Oghbaei M., Prakash J., Yildiz F. (2016). Effect of primary processing of cereals and legumes on its nutritional quality: A comprehensive review. Cogent. Food Agric..

[58] Batifoulier F., Verny M.A., Chanliaud E., Remesy C., Demigne C. (2006). Variability of B vitamin concentrations in wheat grain, milling fractions and bread products. Eur. J. Agron..

[59] Létinois U., Moine G., Hohmann H.P. (2020). 6. Vitamin B1(Thiamin). Ullmann’s Encyclopedia of Industrial Chemistry.

[60] Liu K.L., Zheng J.B., Chen F.S. (2017). Relationships between degree of milling and loss of Vitamin B, minerals, and change in amino acid composition of brown rice. LWT Food Sci. Technol..

[61] Tiozon R.N., Fernie A.R., Sreenivasulu N. (2021). Meeting human dietary vitamin requirements in the staple rice via strategies of biofortification and post-harvest fortification. Trends Food Sci. Technol..

[62] Suri D.J., Tanumihardjo S.A. (2016). Effects of Different Processing Methods on the Micronutrient and Phytochemical Contents of Maize: From A to Z. Compr. Rev. Food Sci. Food Saf..

[63] Gwirtz J.A., Garcia-Casal M.N. (2014). Processing maize flour and corn meal food products. Ann. N. Y. Acad. Sci..

[64] Voelker A.L., Miller J., Running C.A., Taylor L.S., Mauer L.J. (2018). Chemical stability and reaction kinetics of two thiamine salts (thiamine mononitrate and thiamine chloride hydrochloride) in solution. Food Res. Int..

[65] Rekha P.N., Singhal S., Pandit A.B. (2004). A study on degradation kinetics of thiamine in red gram splits (*Cajanus cajan* L.). Food Chem..

[66] European Food Safety Authority (2008). Benfotiamine, thiamine monophosphate chloride and thiamine pyrophosphate chloride, as sources of vitamin B1 added for nutritional purposes to food supplements-Scientific Opinion of the Panel on Food Additives and Nutrient Sources added to Food (ANS). EFSA J..

[67] Voelker A.L., Taylor L.S., Mauer L.J. (2021). Chemical stability and reaction kinetics of thiamine mononitrate in the aqueous phase of bread dough. Food Res. Int..

[68] Dionísio A.P., Gomes R.T., Oetterer M. (2009). Ionizing radiation effects on food vitamins: A review. Braz. Arch. Biol. Technol..

[69] Godoy H.T., Amaya-Farfan J., Rodriguez-Amaya D.B., Rodriguez-Amaya D.B., Amaya-Farfan J. (2021). Degradation of vitamins. Chemical Changes During Processing and Storage of Foods.

[70] Bognár A. (2002). Tables on Weight Yield of Food and Retention Factors of Food Constituents for the Calculation of Nutrient Composition of Cooked Foods (Dishes).

[71] Öhrvik V., Carlsen M.H., Källman A., Martinsen T.A. (2015). Improving Food Composition Data by Standardizing Calculation Methods.

[72] USDA USDA Table of Nutrient Retention Factors. https://www.ars.usda.gov/ARSUserFiles/80400525/Data/retn/retn06.pdf.

[73] Bell S., Becker W., Vásquez-Caicedo A., Hartmann B., Møller A., Butriss J. (2006). Report on Nutrient Losses and Gains Factors Used in European Food Composition Databases.

[74] Lešková E., Kubíková J., Kováčiková E., Košická M., Porubská J., Holčíková K. (2006). Vitamin losses: Retention during heat treatment and continual changes expressed by mathematical models. J. Food Compos. Anal..

[75] Kumar S., Aalbersberg B. (2006). Nutrient retention in foods after earth-oven cooking compared to other forms of domestic cooking-2. Vitamins. J. Food Compos. Anal..

[76] Aktas-Akyildiz E., Koksel H. (2021). Minimisation of vitamin losses in fortified cookies by response surface methodology and validation of the determination methods. Eur. Food Res. Technol..

[77] Fillion L., Henry C.J. (1998). Nutrient losses and gains during frying: A review. Int. J. Food Sci. Nutr..

[78] Lombardi-Boccia G., Lanzi S., Aguzzi A. (2005). Aspects of meat quality: Trace elements and B vitamins in raw and cooked meats. J. Food Compos. Anal..

[79] Bognar A. (1998). Comparative study of frying to other cooking techniques influence on the nutritive value. Grasas Aceites.

[80] Silveira C.M., Moreira A.V., Martino H.S., Gomide R.S., Pinheiro S.S., Della Lucia C.M., Pinheiro-Sant’ana H.M. (2017). Effect of cooking methods on the stability of thiamin and folic acid in fortified rice. Int. J. Food Sci. Nutr..

[81] Jaworska G., Bernas E. (2009). The effect of preliminary processing and period of storage on the quality of frozen *Boletus edulis* (Bull: Fr.) mushrooms. Food Chem..

[82] Liu K., Zheng J., Wang X., Chen F. (2019). Effects of household cooking processes on mineral, vitamin B, and phytic acid contents and mineral bioaccessibility in rice. Food Chem..

[83] Szymandera-Buszka K., Piechocka J., Zaremba A., Przeor M., Jedrusek-Golinska A. (2021). Pumpkin, Cauliflower and Broccoli as New Carriers of Thiamine Compounds for Food Fortification. Foods.

[84] Özdemir M., Açkurt F., Yildiz M., Biringen G., Gürcan T., Löker M. (2001). Effect of roasting on some nutrients of hazelnuts (*Corylus avellena* L.). Food Chem..

[85] Pinheiro-Sant’Ana H.M., Penteado M., Brandão S., Stringheta P. (1999). Stability of B-vitamin in meats prepared by foodservice. 1. Thiamin. Foodserv. Res. Int..

[86] Williams P.G. (1996). Vitamin retention in cook/chill and cook/hot-hold hospital food-services. J. Am. Diet Assoc..

[87] Ryley J., Kajda P. (1994). Vitamins in Thermal-Processing. Food Chem..

[88] Hill M.A. (1994). Vitamin Retention in Microwave Cooking and Cook-Chill Foods. Food Chem..

[89] Severi S., Bedogni G., Manzieri A.M., Poli M., Battistini N. (1997). Effects of cooking and storage methods on the micronutrient content of foods. Eur. J. Cancer Prev..

[90] Hubner F., Arendt E.K. (2013). Germination of cereal grains as a way to improve the nutritional value: A review. Crit. Rev. Food Sci. Nutr..

[91] Freitag S., Verrall S.R., Pont S.D.A., McRae D., Sungurtas J.A., Palau R., Hawes C., Alexander C.J., Allwood J.W., Foito A. (2018). Impact of Conventional and Integrated Management Systems on the Water-Soluble Vitamin Content in Potatoes, Field Beans, and Cereals. J. Agric. Food Chem..

[92] Titcomb T.J., Tanumihardjo S.A. (2019). Global Concerns with B Vitamin Statuses: Biofortification, Fortification, Hidden Hunger, Interactions, and Toxicity. Compr. Rev. Food Sci. Food Saf..

[93] FAO (1995). Sorghum and millets in human nutrition. FAO Food and Nutrition Series.

[94] Malleshi N.G., Klopfenstein C.E. (1998). Nutrient composition, amino acid and vitamin contents of malted sorghum, pearl millet, finger millet and their rootlets. Int. J. Food Sci. Technol..

[95] Pinheiro S.S., Anunciacao P.C., Cardoso L.M., Della Lucia C.M., de Carvalho C.W.P., Queiroz V.A.V., Pinheiro Sant’Ana H.M. (2021). Stability of B vitamins, vitamin E, xanthophylls and flavonoids during germination and maceration of sorghum (*Sorghum bicolor* L.). Food Chem..

[96] Prodanov M., Sierra I., Vidal-Valverde C. (1997). Effect of germination on the thiamine, riboflavin and niacin contents in legumes. Eur. Food Res. Technol..

[97] Frias J., Prodanov M., Sierra I., Vidal-Valverde C. (1995). Effect of Light on Carbohydrates and Hydrosoluble Vitamins of Lentils during Soaking. J. Food Prot..

[98] Roe M., Church S., Pinchen H., Finglas P. (2013). Nutrient Analysis of Fruit and Vegetables.

[99] Garg M., Sharma A., Vats S., Tiwari V., Kumari A., Mishra V., Krishania M. (2021). Vitamins in Cereals: A Critical Review of Content, Health Effects, Processing Losses, Bioaccessibility, Fortification, and Biofortification Strategies for Their Improvement. Front. Nutr..

[100] Maskova E.R., Fiedlerova V., Holasova M. (1994). Vitamin and mineral retention in meat in various cooking methods. Czech J. Food Sci..

[101] USDA USDA Food Composition Databases. https://fdc.nal.usda.gov/.

[102] Roe M., Church S., Pinchen H., Finglas P. (2013). Nutrient Analysis of Fish and Fish Products.

[103] Mattila P., Konko K., Eurola M., Pihlava J.M., Astola J., Vahteristo L., Hietaniemi V., Kumpulainen J., Valtonen M., Piironen V. (2001). Contents of vitamins, mineral elements, and some phenolic compounds in cultivated mushrooms. J. Agric. Food Chem..

[104] Sałata A., Lemieszek M., Parzymies M. (2018). The Nutritional and Health Properties of an Oyster Mushroom (*Pleurotus ostreatus* (Jacq. Fr) P. Kumm.). Acta Sci. Pol. Hortorum Cultus.

[105] Bernaś E., Jaworska G. (2016). Vitamins profile as an indicator of the quality of frozen *Agaricus bisporus* mushrooms. J. Food Compos. Anal..

[106] Hashemi Gahruie H., Eskandari M.H., Mesbahi G., Hanifpour M.A. (2015). Scientific and technical aspects of yogurt fortification: A review. Food Sci. Hum. Wellness.

[107] Roe M., Church S., Pinchen H., Finglas P. (2013). Nutrient Analysis of Eggs.

[108] Awonorin S.O., Rotimi D.K. (1991). Effects of oven temperature and time on the losses of some B vitamins in roasted beef and pork. Foodserv. Res. Int..

[109] Kyritsi A., Tzia C., Karathanos V.T. (2011). Vitamin fortified rice grain using spraying and soaking methods. LWT Food Sci. Technol..

[110] Atungulu G.G., Pan Z. (2014). Rice industrial processing worldwide and impact on macro- and micronutrient content, stability, and retention. Ann. N. Y. Acad. Sci..

[111] Rumm-Kreuter D., Demmel I. (1990). Comparison of vitamin losses in vegetables due to various cooking methods. J. Nutr. Sci. Vitaminol..

[112] Díaz-Gómez J., Twyman R.M., Zhu C., Farré G., Serrano J.C., Portero-Otin M., Muñoz P., Sandmann G., Capell T., Christou P. (2017). Biofortification of crops with nutrients: Factors affecting utilization and storage. Curr. Opin. Biotechnol..

[113] Prodanov M., Sierra I., Vidal-Valverde C. (2004). Influence of soaking and cooking on the thiamin, riboflavin and niacin contents of legumes. Food Chem..

[114] Batifoulier F., Verny M.A., Chanliaud E., Remesy C., Demigne C. (2005). Effect of different breadmaking methods on thiamine, riboflavin and pyridoxine contents of wheat bread. J. Cereal Sci..

[115] Martinez-Villaluenga C., Michalska A., Frias J., Piskula M.K., Vidal-Valverde C., Zielinski H. (2009). Effect of flour extraction rate and baking on thiamine and riboflavin content and antioxidant capacity of traditional rye bread. J. Food Sci..

[116] Haddad G.S., Loewenstein M. (1983). Effect of several heat treatments and frozen storage on thiamine, riboflavin, and ascorbic acid content of milk. J. Dairy Sci..

[117] Graham D.M. (1974). Alteration of nutritive value resulting from processing and fortification of milk and milk products. J. Dairy Sci..

[118] Lima H., Vogel K., Wagner-Gillespie M., Wimer C., Dean L., Fogleman A. (2018). Nutritional Comparison of Raw, Holder Pasteurized, and Shelf-stable Human Milk Products. J. Pediatr. Gastroenterol. Nutr..

[119] Athar N., Hardacre A., Taylor G., Clark S., Harding R., McLaughlin J. (2006). Vitamin retention in extruded food products. J. Food Compos. Anal..

[120] Riaz M.N., Asif M., Ali R. (2009). Stability of vitamins during extrusion. Crit. Rev. Food Sci. Nutr..

[121] Aylangan A., Ic E., Ozyardimci B. (2017). Investigation of gamma irradiation and storage period effects on the nutritional and sensory quality of chickpeas, kidney beans and green lentils. Food Control.

[122] Fox J.B., Thayer D.W., Jenkins R.K., Phillips J.G., Ackerman S.A., Beecher G.R., Holden J.M., Morrow F.D., Quirbach D.M. (1989). Effect of gamma irradiation on the B vitamins of pork chops and chicken breasts. Int. J. Radiat. Biol..

[123] Fox J.B., Lakritz L., Hampson J.R., Ward K., Thayer D.W. (1995). Gamma Irradiation Effects on Thiamin and Riboflavin in Beef, Lamb, Pork, and Turkey. J. Food Sci..

[124] Woodside J. (2015). Nutritional aspects of irradiated food. Stewart Postharvest Rev..

[125] Greenwood D.A., Kraybill H.R., Feaster J.F., Jackson J.M. (1944). Vitamin Retention in Processed Meat. Ind. Eng. Chem..

[126] Rickman J.C., Barrett D.M., Bruhn C.M. (2007). Nutritional comparison of fresh, frozen and canned fruits and vegetables. Part 1. Vitamins C and B and phenolic compounds. J. Sci. Food Agric..

[127] Martín-Belloso O., Llanos-Barriobero E. (2001). Proximate composition, minerals and vitamins in selected canned vegetables. Eur. Food Res. Technol..

[128] Marçal S., Sousa A.S., Taofiq O., Antunes F., Morais A.M., Freitas A.C., Barros L., Ferreira I.C., Pintado M. (2021). Impact of postharvest preservation methods on nutritional value and bioactive properties of mushrooms. Trends Food Sci. Technol..

[129] Coad R., Bui L. (2020). Stability of Vitamins B1, B2, B6 and E in a Fortified Military Freeze-Dried Meal During Extended Storage. Foods.

[130] Ayhan D.K., Koksel H. (2019). Investigation of the effect of different storage conditions on vitamin content of enriched pasta product. Qual. Assur. Saf. Crops.

[131] Walker G.J. (1979). The nutritional value of processed foods. CSIRO Food Proc..

[132] Gan R.Y., Lui W.Y., Wu K., Chan C.L., Dai S.H., Sui Z.Q., Corke H. (2017). Bioactive compounds and bioactivities of germinated edible seeds and sprouts: An updated review. Trends Food Sci. Technol..

[133] Lemmens E., Moroni A.V., Pagand J., Heirbaut P., Ritala A., Karlen Y., Le K.A., Van den Broeck H.C., Brouns F., De Brier N. (2019). Impact of Cereal Seed Sprouting on Its Nutritional and Technological Properties: A Critical Review. Compr. Rev. Food Sci. Food Saf..

[134] Lonsdale D. (2004). Thiamine tetrahydrofurfuryl disulfide: A little known therapeutic agent. Med. Sci. Monit..

[135] Fujiwara M., Watanabe H., Matsui K. (1954). “Allithiamine” a Newly Found Derivative of Vitamin B1. J. Biochem..

[136] Fujiwara M. (1976). Allithiamine and its properties. J. Nutr. Sci. Vitam..

[137] Matsukawa T., Kawasaki H., Iwatsu T., Yurugi S. (1954). Syntheses of allithiamine and its homologues. J. Vitam..

[138] Miah M.A.K., Haque A., Douglass M.P., Clarke B. (2002). Parboiling of rice. Part II: Effect of hot soaking time on the degree of starch gelatinization. Int. J. Food Sci. Technol..

[139] Ituen E., Ukpakha A. (2011). Improved method of par-boiling paddy for better quality rice. World J. Appl. Sci. Technol..

[140] Oli P., Ward R., Adhikari B., Torley P. (2014). Parboiled rice: Understanding from a materials science approach. J. Food Eng..

[141] Hinton J.J. (1948). Parboiling treatment of rice. Nature.

[142] Villota S.M.A., Tuates A.M., Capariño O.A. (2016). Cooking Qualilites and Nutritional Contents of Parboiled Milled Rice. Asian J. Appl. Sci..

[143] Manful J., Swetman A., Coker R., Drunis A. (2007). Changes in the thiamine and riboflavin contents of rice during artisanal parboiling in Ghana. Trop. Sci..

[144] Padua A.B., Juliano B.O. (1974). Effect of parboiling on thiamin, protein and fat of rice. J. Sci. Food Agric..

[145] Cubadda F., Jackson B.P., Cottingham K.L., Van Horne Y.O., Kurzius-Spencer M. (2017). Human exposure to dietary inorganic arsenic and other arsenic species: State of knowledge, gaps and uncertainties. Sci. Total Environ..

[146] Davis M.A., Signes-Pastor A.J., Argos M., Slaughter F., Pendergrast C., Punshon T., Gossai A., Ahsan H., Karagas M.R. (2017). Assessment of human dietary exposure to arsenic through rice. Sci. Total Environ..

[147] Sun G.X., Williams P.N., Carey A.M., Zhu Y.G., Deacon C., Raab A., Feldmann J., Islam R.M., Meharg A.A. (2008). Inorganic arsenic in rice bran and its products are an order of magnitude higher than in bulk grain. Environ. Sci. Technol..

[148] Lombi E., Scheckel K.G., Pallon J., Carey A.M., Zhu Y.G., Meharg A.A. (2009). Speciation and distribution of arsenic and localization of nutrients in rice grains. New Phytol..

[149] Meharg A.A., Lombi E., Williams P.N., Scheckel K.G., Feldmann J., Raab A., Zhu Y., Islam R. (2008). Speciation and localization of arsenic in white and brown rice grains. Environ. Sci. Technol..

[150] Wang X., Peng B., Tan C., Ma L., Rathinasabapathi B. (2015). Recent advances in arsenic bioavailability, transport, and speciation in rice. Environ. Sci. Pollut. Res. Int..

[151] USFDA, U.S.F.a.D Arsenic in Rice and Rice Products Risk Assessment Report. http://www.fda.gov/Food/FoodScienceResearch/RiskSafetyAssessment/default.htm.

[152] Upadhyay M.K., Shukla A., Yadav P., Srivastava S. (2019). A review of arsenic in crops, vegetables, animals and food products. Food Chem..

[153] Arcella D., Cascio C., Gomez Ruiz J.A., European Food Safety Authority (2021). Chronic dietary exposure to inorganic arsenic. EFSA J..

[154] EFSA (2014). Dietary exposure to inorganic arsenic in the European population. EFSA J..

[155] Rasheed H., Kay P., Slack R., Gong Y.Y. (2018). Arsenic species in wheat, raw and cooked rice: Exposure and associated health implications. Sci. Total Environ..

[156] Lai P.Y., Cottingham K.L., Steinmaus C., Karagas M.R., Miller M.D. (2015). Arsenic and Rice: Translating Research to Address Health Care Providers’ Needs. J. Pediatr..

[157] Islam S., Rahman M.M., Rahman M.A., Naidu R. (2017). Inorganic arsenic in rice and rice-based diets: Health risk assessment. Food Control.

[158] Mwale T., Rahman M.M., Mondal D. (2018). Risk and Benefit of Different Cooking Methods on Essential Elements and Arsenic in Rice. Int. J. Environ. Res. Public Health.

[159] Nachman K.E., Ginsberg G.L., Miller M.D., Murray C.J., Nigra A.E., Pendergrast C.B. (2017). Mitigating dietary arsenic exposure: Current status in the United States and recommendations for an improved path forward. Sci. Total Environ..

[160] IARC Working Group on the Evaluation of Carcinogenic Risks to Humans, International Agency for Research on Cancer, and World Health Organization (2012). Arsenic, metals, fibres, and dusts. IARC Monogr. Eval. Carcinog. Risks Hum..

[161] EFSA (2009). Scientific Opinion on Arsenic in Food. EFSA J..

[162] Rahman M.A., Rahman A., Khan M.Z.K., Renzaho A.M.N. (2018). Human health risks and socio-economic perspectives of arsenic exposure in Bangladesh: A scoping review. Ecotoxicol. Environ. Saf..

[163] Gray P.J., Conklin S.D., Todorov T.I., Kasko S.M. (2016). Cooking rice in excess water reduces both arsenic and enriched vitamins in the cooked grain. Food Addit. Contam. Part A.

[164] Kumarathilaka P., Seneweera S., Ok Y.S., Meharg A., Bundschuh J. (2019). Arsenic in cooked rice foods: Assessing health risks and mitigation options. Environ. Int..

[165] Pedron T., Segura F.R., Paniz F.P., Souz F.D., dos Santos M.C., de Magalhaes A.M., Batista B.L. (2019). Mitigation of arsenic in rice grains by polishing and washing: Evidencing the benefit and the cost. J. Cereal Sci..

[166] Menon M., Dong W., Chen X., Hufton J., Rhodes E.J. (2021). Improved rice cooking approach to maximise arsenic removal while preserving nutrient elements. Sci. Total Environ..

[167] Naito S., Matsumoto E., Shindoh K., Nishimura T. (2015). Effects of polishing, cooking, and storing on total arsenic and arsenic species concentrations in rice cultivated in Japan. Food Chem..

[168] Atiaga O., Nunes L.M., Otero X.L. (2020). Effect of cooking on arsenic concentration in rice. Environ. Sci. Pollut. Res. Int..

[169] Raab A., Baskaran C., Feldmann J., Meharg A.A. (2009). Cooking rice in a high water to rice ratio reduces inorganic arsenic content. J. Environ. Monit..

[170] Eggersdorfer M., Laudert D., Letinois U., McClymont T., Medlock J., Netscher T., Bonrath W. (2012). One hundred years of vitamins-a success story of the natural sciences. Angew. Chem. Int. Ed. Engl..

[171] Acevedo-Rocha C.G., Gronenberg L.S., Mack M., Commichau F.M., Genee H.J. (2019). Microbial cell factories for the sustainable manufacturing of B vitamins. Curr. Opin. Biotechnol..

[172] Fitzpatrick T.B., Basset G.J., Borel P., Carrari F., DellaPenna D., Fraser P.D., Hellmann H., Osorio S., Rothan C., Valpuesta V. (2012). Vitamin deficiencies in humans: Can plant science help?. Plant Cell.

[173] Fulgoni V.L., Keast D.R., Bailey R.L., Dwyer J. (2011). Foods, fortificants, and supplements: Where do Americans get their nutrients?. J. Nutr..

[174] Liberato S.C., Pinheiro-Sant’Ana H.M. (2006). Fortification of industrialized foods with vitamins. Rev. Nutr..

[175] Berner L.A., Keast D.R., Bailey R.L., Dwyer J.T. (2014). Fortified foods are major contributors to nutrient intakes in diets of US children and adolescents. J. Acad. Nutr. Diet.

[176] Whitfield K.C., Smith T.J., Rohner F., Wieringa F.T., Green T.J. (2021). Thiamine fortification strategies in low- and middle-income settings: A review. Ann. N. Y. Acad. Sci..

[177] Allen L., Benoist B., Dary O., Hurrell R. (2006). Guidelines on Food Fortification with Micronutrients.

[178] Newman J.C., Malek A.M., Hunt K.J., Marriott B.P. (2019). Nutrients in the US Diet: Naturally Occurring or Enriched/Fortified Food and Beverage Sources, Plus Dietary Supplements: NHANES 2009–2012. J. Nutr..

[179] EU Parliament (2006). E. Regulation (EC) No 1925/2006 of the European Parliament and of the Council of 20 December 2006 on the addition of vitamins and minerals and of certain other substances to foods. OJ L 404. https://eur-lex.europa.eu/legal-content/EN/ALL/?uri=CELEX%3A32006R1925.

[180] Gomes F., Bergeron G., Bourassa M.W., Fischer P.R. (2021). Thiamine deficiency unrelated to alcohol consumption in high-income countries: A literature review. Ann. N. Y. Acad. Sci..

[181] GFD Global Fortification Data Exchange. https://fortificationdata.org/.

[182] FFI Food Fortification Initiative. https://www.ffinetwork.org/country-profiles.

[183] De Pee S. (2014). Proposing nutrients and nutrient levels for rice fortification. Ann. N. Y. Acad. Sci..

[184] Saha S., Roy A. (2020). Whole grain rice fortification as a solution to micronutrient deficiency: Technologies and need for more viable alternatives. Food Chem..

[185] Steiger G., Muller-Fischer N., Cori H., Conde-Petit B. (2014). Fortification of rice: Technologies and nutrients. Ann. N. Y. Acad. Sci..

[186] Alavi S., Bugusu B., Cramer G., Dary O., Lee T.-C., Martin L., McEntire J., Wailes E. (2008). Rice Fortification in Developing Countries: A Critical Review of the Technical and Economic Feasibility.

[187] Strobbe S., Van Der Straeten D. (2018). Toward Eradication of B-Vitamin Deficiencies: Considerations for Crop Biofortification. Front. Plant Sci..

[188] Minhas A.P., Tuli R., Puri S. (2018). Pathway Editing Targets for Thiamine Biofortification in Rice Grains. Front. Plant Sci..

[189] Dong W., Thomas N., Ronald P.C., Goyer A. (2016). Overexpression of thiamin biosynthesis genes in rice increases leaf and unpolished grain thiamin content but not resistance to *Xanthomonas oryzae pv. oryzae*. Front. Plant Sci..

[190] Strobbe S., Verstraete J., Stove C., Van Der Straeten D. (2021). Metabolic engineering of rice endosperm towards higher vitamin B1 accumulation. Plant Biotechnol. J..

[191] Goyer A. (2017). Thiamin biofortification of crops. Curr. Opin. Biotechnol..

[192] Smithline H.A., Donnino M., Greenblatt D.J. (2012). Pharmacokinetics of high-dose oral thiamine hydrochloride in healthy subjects. BMC Clin. Pharmacol..

[193] Gangolf M., Czerniecki J., Radermecker M., Detry O., Nisolle M., Jouan C., Martin D., Chantraine F., Lakaye B., Wins P. (2010). Thiamine status in humans and content of phosphorylated thiamine derivatives in biopsies and cultured cells. PLoS ONE.

[194] Rindi G., Laforenza U. (2000). Thiamine intestinal transport and related issues: Recent aspects. Proc. Soc. Exp. Biol. Med..

[195] Said H.M., Balamurugan K., Subramanian V.S., Marchant J.S. (2004). Expression and functional contribution of hTHTR-2 in thiamin absorption in human intestine. Am. J. Physiol. Gastrointest. Liver. Physiol..

[196] Ganapathy V., Smith S.B., Prasad P.D. (2004). SLC19: The folate/thiamine transporter family. Pflug. Arch..

[197] Nabokina S.M., Said H.M. (2012). A high-affinity and specific carrier-mediated mechanism for uptake of thiamine pyrophosphate by human colonic epithelial cells. Am. J. Physiol. Gastrointest. Liver. Physiol..

[198] Ott M., Werneke U. (2020). Wernicke’s encephalopathy-from basic science to clinical practice. Part 1: Understanding the role of thiamine. Adv. Ther. Psychopharmacol..

[199] Lu J., Frank E.L. (2008). Rapid HPLC measurement of thiamine and its phosphate esters in whole blood. Clin. Chem..

[200] Labay V., Raz T., Baron D., Mandel H., Williams H., Barrett T., Szargel R., McDonald L., Shalata A., Nosaka K. (1999). Mutations in SLC19A2 cause thiamine-responsive megaloblastic anaemia associated with diabetes mellitus and deafness. Nat. Genet..

[201] Zhao R., Gao F., Goldman I.D. (2001). Molecular cloning of human thiamin pyrophosphokinase. Biochim. Biophys. Acta.

[202] Bettendorff L. (1994). The compartmentation of phosphorylated thiamine derivatives in cultured neuroblastoma cells. Biochim. Biophys. Acta.

[203] Eudy J.D., Spiegelstein O., Barber R.C., Wlodarczyk B.J., Talbot J., Finnell R.H. (2000). Identification and characterization of the human and mouse SLC19A3 gene: A novel member of the reduced folate family of micronutrient transporter genes. Mol. Genet. Metab..

[204] Casteels M., Sniekers M., Fraccascia P., Mannaerts G.P., Van Veldhoven P.P. (2007). The role of 2-hydroxyacyl-CoA lyase, a thiamin pyrophosphate-dependent enzyme, in the peroxisomal metabolism of 3-methyl-branched fatty acids and 2-hydroxy straight-chain fatty acids. Biochem. Soc. Trans..

[205] Ashokkumar B., Vaziri N.D., Said H.M. (2006). Thiamin uptake by the human-derived renal epithelial (HEK-293) cells: Cellular and molecular mechanisms. Am. J. Physiol. Ren. Physiol..

[206] Patel M.S., Nemeria N.S., Furey W., Jordan F. (2014). The pyruvate dehydrogenase complexes: Structure-based function and regulation. J. Biol. Chem..

[207] Hutson S.M., Sweatt A.J., Lanoue K.F. (2005). Branched-chain amino acid metabolism: Implications for establishing safe intakes. J. Nutr..

[208] Sperringer J.E., Addington A., Hutson S.M. (2017). Branched-Chain Amino Acids and Brain Metabolism. Neurochem. Res..

[209] Schenk G., Duggleby R.G., Nixon P.F. (1998). Properties and functions of the thiamin diphosphate dependent enzyme transketolase. Int. J. Biochem. Cell Biol..

[210] Foulon V., Sniekers M., Huysmans E., Asselberghs S., Mahieu V., Mannaerts G.P., Van Veldhoven P.P., Casteels M. (2005). Breakdown of 2-hydroxylated straight chain fatty acids via peroxisomal 2-hydroxyphytanoyl-CoA lyase: A revised pathway for the alpha-oxidation of straight chain fatty acids. J. Biol. Chem..

[211] Lonsdale D. (2018). Thiamin. Adv. Food. Nutr. Res..

[212] Kelley R.I., Robinson D., Puffenberger E.G., Strauss K.A., Morton D.H. (2002). Amish lethal microcephaly: A new metabolic disorder with severe congenital microcephaly and 2-ketoglutaric aciduria. Am. J. Med. Genet..

[213] Marce-Grau A., Marti-Sanchez L., Baide-Mairena H., Ortigoza-Escobar J.D., Perez-Duenas B. (2019). Genetic defects of thiamine transport and metabolism: A review of clinical phenotypes, genetics, and functional studies. J. Inherit. Metab. Dis..

[214] Shible A.A., Ramadurai D., Gergen D., Reynolds P.M. (2019). Dry Beriberi Due to Thiamine Deficiency Associated with Peripheral Neuropathy and Wernicke’s Encephalopathy Mimicking Guillain-Barre syndrome: A Case Report and Review of the Literature. Am. J. Case Rep..

[215] Chisolm-Straker M., Cherkas D. (2013). Altered and unstable: Wet beriberi, a clinical review. J. Emerg. Med..

[216] DiNicolantonio J.J., Liu J., O’Keefe J.H. (2018). Thiamine and Cardiovascular Disease: A Literature Review. Prog. Cardiovasc. Dis..

[217] Greenspon J., Perrone E.E., Alaish S.M. (2010). Shoshin beriberi mimicking central line sepsis in a child with short bowel syndrome. World J. Pediatr..

[218] Dabar G., Harmouche C., Habr B., Riachi M., Jaber B. (2015). Shoshin Beriberi in Critically-Ill patients: Case series. Nutr. J..

[219] Fattal-Valevski A., Bloch-Mimouni A., Kivity S., Heyman E., Brezner A., Strausberg R., Inbar D., Kramer U., Goldberg-Stern H. (2009). Epilepsy in children with infantile thiamine deficiency. Neurology.

[220] Nazir M., Lone R., Charoo B.A. (2019). Infantile Thiamine Deficiency: New Insights into an Old Disease. Indian Pediatr..

[221] Chandrakumar A., Bhardwaj A., Geert W., Jong G.W. (2018). Review of thiamine deficiency disorders: Wernicke encephalopathy and Korsakoff psychosis. J. Basic Clin. Physiol. Pharmacol..

[222] Butterworth R.F. (2003). Thiamin deficiency and brain disorders. Nutr. Res. Rev..

[223] Kopelman M.D., Thomson A.D., Guerrini I., Marshall E.J. (2009). The Korsakoff syndrome: Clinical aspects, psychology and treatment. Alcohol Alcohol..

[224] Arts N.J., Walvoort S.J., Kessels R.P. (2017). Korsakoff’s syndrome: A critical review. Neuropsychiatr. Dis. Treat..

[225] Kril J.J., Harper C.G. (2012). Neuroanatomy and neuropathology associated with Korsakoff’s syndrome. Neuropsychol. Rev..

[226] Yates A.A., Schlicker S.A., Suitor C.W. (1998). Dietary Reference Intakes: The new basis for recommendations for calcium and related nutrients, B vitamins, and choline. J. Am. Diet Assoc..

[227] Armah S., Ferruzzi M.G., Gletsu-Miller N. (2020). Feasibility of Mass-Spectrometry to Lower Cost and Blood Volume Requirements for Assessment of B Vitamins in Patients Undergoing Bariatric Surgery. Metabolites.

[228] Bishop A.M., Fernandez C., Whitehead R.D., Morales A.P., Barr D.B., Wilder L.C., Baker S.E. (2011). Quantification of riboflavin in human urine using high performance liquid chromatography-tandem mass spectrometry. J. Chromatogr. B Anal. Technol. Biomed. Life Sci..

[229] Diniz M., Dias N., Andrade F., Paulo B., Ferreira A. (2019). Isotope dilution method for determination of vitamin B2 in human plasma using liquid chromatography-tandem mass spectrometry. J. Chromatogr. B Anal. Technol. Biomed. Life Sci..

[230] Hampel D., York E.R., Allen L.H. (2012). Ultra-performance liquid chromatography tandem mass-spectrometry (UPLC-MS/MS) for the rapid, simultaneous analysis of thiamin, riboflavin, flavin adenine dinucleotide, nicotinamide and pyridoxal in human milk. J. Chromatogr. B Anal. Technol. Biomed. Life Sci..

[231] Cheng X., Ma D., Fei G., Ma Z., Xiao F., Yu Q., Pan X., Zhou F., Zhao L., Zhong C. (2018). A single-step method for simultaneous quantification of thiamine and its phosphate esters in whole blood sample by ultra-performance liquid chromatography-mass spectrometry. J. Chromatogr. B Anal. Technol. Biomed. Life Sci..

[232] Jeong Hyeon M., Shin Beom S., Shin S. (2020). Liquid Chromatography-Tandem Mass Spectrometry Analysis of Riboflavin in Beagle Dog Plasma for Pharmacokinetic Studies. Mass Spectrom. Lett..

[233] Kahoun D., Fojtíková P., Vácha F., Nováková E., Hypša V. (2021). Development and validation of an LC-MS/MS method for determination of B vitamins and some its derivatives in whole blood. bioRxiv.

[234] Khaksari M., Mazzoleni L.R., Ruan C.H., Song P., Hershey N.D., Kennedy R.T., Burns M.A., Minerick A.R. (2018). Detection and quantification of vitamins in microliter volumes of biological samples by LC-MS for clinical screening. Aiche J..

[235] Meisser Redeuil K., Longet K., Benet S., Munari C., Campos-Gimenez E. (2015). Simultaneous quantification of 21 water soluble vitamin circulating forms in human plasma by liquid chromatography-mass spectrometry. J. Chromatogr. A.

[236] Ren X.N., Yin S.A., Yang Z.Y., Yang X.G., Shao B., Ren Y.P., Zhang J. (2015). Application of UPLC-MS/MS Method for Analyzing B-vitamins in Human Milk. Biomed. Environ. Sci..

[237] Roelofsen-de Beer R., Van Zelst B.D., Wardle R., Kooij P.G., de Rijke Y.B. (2017). Simultaneous measurement of whole blood vitamin B1 and vitamin B6 using LC-ESI-MS/MS. J. Chromatogr. B Anal. Technol. Biomed. Life Sci..

[238] Verstraete J., Stove C. (2021). Patient-Centric Assessment of Thiamine Status in Dried Blood Volumetric Absorptive Microsamples Using LC-MS/MS Analysis. Anal. Chem..

[239] Zhang Q., Ford L.A., Goodman K.D., Freed T.A., Hauser D.M., Conner J.K., Vroom K.E., Toal D.R. (2016). LC-MS/MS method for quantitation of seven biomarkers in human plasma for the assessment of insulin resistance and impaired glucose tolerance. J. Chromatogr. B Anal. Technol. Biomed. Life Sci..

[240] Huang Y., Gibson R.A., Green T.J. (2020). Measuring thiamine status in dried blood spots. Clin. Chim. Acta.

[241] Jenčo J., Krčmová L.K., Sobotka L., Bláha V., Solich P., Švec F. (2020). Development of novel liquid chromatography method for clinical monitoring of vitamin B1 metabolites and B6 status in the whole blood. Talanta.

[242] Mathew E.M., Sakore P., Lewis L., Manokaran K., Rao P., Moorkoth S. (2019). Development and validation of a dried blood spot test for thiamine deficiency among infants by HPLC-fluorimetry. Biomed. Chromatogr..

[243] Nguyen V.L., Darman M., Ireland A., Fitzpatrick M. (2020). A high performance liquid chromatography fluorescence method for the analysis of both pyridoxal-5-phosphate and thiamine pyrophosphate in whole blood. Clin. Chim. Acta.

[244] Petteys B.J., Frank E.L. (2011). Rapid determination of vitamin B(2) (riboflavin) in plasma by HPLC. Clin. Chim. Acta.

[245] Stuetz W., Carrara V.I., McGready R., Lee S.J., Biesalski H.K., Nosten F.H. (2012). Thiamine diphosphate in whole blood, thiamine and thiamine monophosphate in breast-milk in a refugee population. PLoS ONE.

[246] Heydari R., Elyasi N.S. (2014). Ion-pair cloud-point extraction: A new method for the determination of water-soluble vitamins in plasma and urine. J. Sep. Sci..

[247] Mandal S.M., Mandal M., Ghosh A.K., Dey S. (2009). Rapid determination of vitamin B2 and B12 in human urine by isocratic liquid chromatography. Anal. Chim. Acta.

[248] Asgharian Marzabad M., Jafari B., Norouzi P. (2020). Determination of Riboflavin by Nanocomposite Modified Carbon Paste Electrode in Biological Fluids Using Fast Fourier Transform Square Wave Voltammetry. Int. J. Eng..

[249] Prasad B.B., Singh R., Singh K. (2017). Development of highly electrocatalytic and electroconducting imprinted film using Ni nanomer for ultra-trace detection of thiamine. Sens. Actuators B Chem..

[250] Shankar S., John S.A. (2015). Sensitive and highly selective determination of vitamin B1 in the presence of other vitamin B complexes using functionalized gold nanoparticles as fluorophore. Rsc. Adv..

[251] Song Z., Hou S. (2002). Determination of picomole amounts of thiamine through flow-injection analysis based on the suppression of luminol-KIO(4) chemiluminescence system. J. Pharm. Biomed. Anal..

[252] Zhang H., Chen H., Li H., Pan S., Ran Y., Hu X. (2018). Construction of a novel turn-on-off fluorescence sensor used for highly selective detection of thiamine via its quenching effect on o-phen-Zn(2^+^) complex. Luminescence.

[253] Immundiagnostik AG. ID-Vit^®^ Pantothenic acid. https://www.immundiagnostik.com/media/pages/testkits/kif004/1c6c7f961a-1633917660/kif004_2019-05-23_pantothensaeure.pdf.

[254] Immundiagnostik AG. ID-Vit^®^ Niacin. https://www.immundiagnostik.com/media/pages/testkits/kif003/2d1c628e3b-1633917660/kif003_2019-05-23_niacin.pdf.

[255] RECIPE Chemicals+Instruments GmbH VITAMIN B1, B2 AND B6 (COMBIKIT). https://recipe.de/products/combikit-vitamin-b1-b2-b6-whole-blood/.

[256] Immundiagnostik AG. Vitamin B1 HPLC Kit. https://www.immundiagnostik.com/media/pages/testkits/kc2201/59011e2c72-1633658467/vitamin-b1_kc2201.pdf.

[257] RECIPE Chemicals+Instruments GmbH VITAMIN B1. https://recipe.de/products/vitamin-b1-whole-blood/.

[258] RECIPE Chemicals+Instruments GmbH VITAMIN B2. https://recipe.de/products/vitamin-b2-whole-blood/.

[259] MYBioSource Thiamine Elisa Kit: Human Thiamine ELISA Kit. https://www.mybiosource.com/human-elisa-kits/thiamine/167383.

[260] LSBio Vitamin B2/Riboflavin (Competitive EIA) ELISA Kit-LS-F55485. https://www.lsbio.com/elisakits/vitamin-b2-riboflavin-competitive-eia-elisa-kit-ls-f55485/55485.

[261] Antibodiesonline GmbH Vitamin B2 (Riboflavin) ELISA Kit. https://www.antibodies-online.com/kit/1059863/Vitamin+B2+Riboflavin+ELISA+Kit/.

[262] Amrein K., Oudemans-van Straaten H.M., Berger M.M. (2018). Vitamin therapy in critically ill patients: Focus on thiamine, vitamin C, and vitamin D. Intensive Care Med..

[263] Russell R.M., Suter P.M. (1993). Vitamin requirements of elderly people: An update. Am. J. Clin. Nutr..

[264] Thomson A., Guerrini I., Marshall E.J. (2019). Incidence of Adverse Reactions to Parenteral Thiamine in the Treatment of Wernicke’s Encephalopathy, and Recommendations. Alcohol Alcohol..

[265] Claus D., Eggers R., Warecka K., Neundorfer B. (1985). Thiamine deficiency and nervous system function disturbances. Eur. Arch. Psych. Neurol. Sci..

[266] Alaei Shahmiri F., Soares M.J., Zhao Y., Sherriff J. (2013). High-dose thiamine supplementation improves glucose tolerance in hyperglycemic individuals: A randomized, double-blind cross-over trial. Eur. J. Nutr..

[267] Gibson G.E., Hirsch J.A., Cirio R.T., Jordan B.D., Fonzetti P., Elder J. (2013). Abnormal thiamine-dependent processes in Alzheimer’s Disease. Lessons from diabetes. Mol. Cell. Neurosci..

[268] Kv L.N., Nguyen L.T. (2013). The role of thiamine in HIV infection. Int. J. Infect. Dis..

[269] Volvert M.L., Seyen S., Piette M., Evrard B., Gangolf M., Plumier J.C., Bettendorff L. (2008). Benfotiamine, a synthetic S-acyl thiamine derivative, has different mechanisms of action and a different pharmacological profile than lipid-soluble thiamine disulfide derivatives. BMC Pharmacol..

[270] Loew D. (1996). Pharmacokinetics of thiamine derivatives especially of benfotiamine. Int. J. Clin. Pharmacol. Ther..

[271] Nishikawa T., Edelstein D., Du X.L., Yamagishi S., Matsumura T., Kaneda Y., Yorek M.A., Beebe D., Oates P.J., Hammes H.P. (2000). Normalizing mitochondrial superoxide production blocks three pathways of hyperglycaemic damage. Nature.

[272] Raj V., Ojha S., Howarth F.C., Belur P.D., Subramanya S.B. (2018). Therapeutic potential of benfotiamine and its molecular targets. Eur. Rev. Med. Pharmacol. Sci..

[273] Babaei-Jadidi R., Karachalias N., Ahmed N., Battah S., Thornalley P.J. (2003). Prevention of incipient diabetic nephropathy by high-dose thiamine and benfotiamine. Diabetes.

[274] Stracke H., Lindemann A., Federlin K. (1996). A benfotiamine-vitamin B combination in treatment of diabetic polyneuropathy. Exp. Clin. Endocrinol. Diabetes.

[275] Huang W.C., Huang H.Y., Hsu Y.J., Su W.H., Shen S.Y., Lee M.C., Lin C.L., Huang C.C. (2018). The Effects of Thiamine Tetrahydrofurfuryl Disulfide on Physiological Adaption and Exercise Performance Improvement. Nutrients.

[276] Scientific Committee on Food (2001). Opinion of the Scientific Committee on Food on the Tolerable Upper Intake Level of Vitamin B1.

[277] Wrenn K.D., Murphy F., Slovis C.M. (1989). A toxicity study of parenteral thiamine hydrochloride. Ann. Emerg. Med..

[278] Sica D.A. (2007). Loop diuretic therapy, thiamine balance, and heart failure. Congest. Heart Fail..

[279] Schumann K. (1999). Interactions between drugs and vitamins at advanced age. Int. J. Vitam. Nutr. Res..

[280] Vora B., Green E.A.E., Khuri N., Ballgren F., Sirota M., Giacomini K.M. (2020). Drug-nutrient interactions: Discovering prescription drug inhibitors of the thiamine transporter ThTR-2 (SLC19A3). Am. J. Clin. Nutr..

[281] Giacomini M.M., Hao J., Liang X., Chandrasekhar J., Twelves J., Whitney J.A., Lepist E.I., Ray A.S. (2017). Interaction of 2,4-Diaminopyrimidine-Containing Drugs Including Fedratinib and Trimethoprim with Thiamine Transporters. Drug Metab. Dispos..

[282] Hohmann H.P., Bretzel W., Hans M., Friedel A., Litta G., Lehmann M., Kurth R., Paust J., Haehnlein W. (2020). Vitamins, 7. Vitamin B2 (Riboflavin). Ullmann’s Encyclopedia of Industrial Chemistry.

[283] Saedisomeolia A., Ashoori M. (2018). Riboflavin in Human Health: A Review of Current Evidences. Adv. Food Nutr. Res..

[284] Powers H.J. (2003). Riboflavin (vitamin B-2) and health. Am. J. Clin. Nutr..

[285] Mestdagh F., De Meulenaer B., De Clippeleer J., Devlieghere F., Huyghebaert A. (2005). Protective influence of several packaging materials on light oxidation of milk. J. Dairy Sci..

[286] Cardoso D.R., Libardi S.H., Skibsted L.H. (2012). Riboflavin as a photosensitizer. Effects on human health and food quality. Food Funct..

[287] Sheraz M.A., Kazi S.H., Ahmed S., Anwar Z., Ahmad I. (2014). Photo, thermal and chemical degradation of riboflavin. Beilstein J. Org. Chem..

[288] Choe E., Huang R.M., Min D.B. (2005). Chemical reactions and stability of riboflavin in foods. J. Food Sci..

[289] Gaylord A.M., Warthesen J.J., Smith D.E. (1986). Influence of milk fat, milk solids, and light intensity on the light stability of vitamin A and riboflavin in lowfat milk. J. Dairy Sci..

[290] Semba R.D. (2012). The discovery of the vitamins. Int. J. Vitam. Nutr. Res..

[291] Northrop-Clewes C.A., Thurnham D.I. (2012). The discovery and characterization of riboflavin. Ann. Nutr. Metab..

[292] Fischer M., Bacher A. (2011). Biosynthesis of vitamin B2 and flavocoenzymes in plants. Adv. Bot. Res..

[293] Fischer M., Bacher A. (2008). Biosynthesis of vitamin B2: Structure and mechanism of riboflavin synthase. Arch. Biochem. Biophys..

[294] Fischer M., Bacher A. (2011). Biosynthesis of vitamin B2: A unique way to assemble a xylene ring. Chembiochem.

[295] Bacher A., Eberhardt S., Fischer M., Kis K., Richter G. (2000). Biosynthesis of vitamin b2 (riboflavin). Annu. Rev. Nutr..

[296] Garcia-Angulo V.A. (2017). Overlapping riboflavin supply pathways in bacteria. Crit. Rev. Microbiol..

[297] Gutierrez-Preciado A., Torres A.G., Merino E., Bonomi H.R., Goldbaum F.A., Garcia-Angulo V.A. (2015). Extensive Identification of Bacterial Riboflavin Transporters and Their Distribution across Bacterial Species. PLoS ONE.

[298] Zylberman V., Klinke S., Haase I., Bacher A., Fischer M., Goldbaum F.A. (2006). Evolution of vitamin B2 biosynthesis: 6,7-dimethyl-8-ribityllumazine synthases of Brucella. J. Bacteriol..

[299] Schwechheimer S.K., Park E.Y., Revuelta J.L., Becker J., Wittmann C. (2016). Biotechnology of riboflavin. Appl. Microbiol. Biotechnol..

[300] Zhang J.-R., Ge Y.-Y., Liu P.-H., Wu D.-T., Liu H.-Y., Li H.-B., Corke H., Gan R.-Y. (2021). Biotechnological Strategies of Riboflavin Biosynthesis in Microbes. Engineering.

[301] Revuelta J.L., Ledesma-Amaro R., Lozano-Martinez P., Diaz-Fernandez D., Buey R.M., Jimenez A. (2017). Bioproduction of riboflavin: A bright yellow history. J. Ind. Microbiol. Biotechnol..

[302] Auclair O., Han Y., Burgos S.A. (2019). Consumption of Milk and Alternatives and Their Contribution to Nutrient Intakes among Canadian Adults: Evidence from the 2015 Canadian Community Health Survey-Nutrition. Nutrients.

[303] Mielgo-Ayuso J., Aparicio-Ugarriza R., Olza J., Aranceta-Bartrina J., Gil A., Ortega R.M., Serra-Majem L., Varela-Moreiras G., Gonzalez-Gross M. (2018). Dietary Intake and Food Sources of Niacin, Riboflavin, Thiamin and Vitamin B (6) in a Representative Sample of the Spanish Population. The Anthropometry, Intake, and Energy Balance in Spain (ANIBES) Study dagger. Nutrients.

[304] Gorska-Warsewicz H., Rejman K., Laskowski W., Czeczotko M. (2019). Milk and Dairy Products and Their Nutritional Contribution to the Average Polish Diet. Nutrients.

[305] Turck D., Bresson J.L., Burlingame B., Dean T., Fairweather-Tait S., Heinonen M., Hirsch-Ernst K.I., Mangelsdorf I., Efsa Panel on Dietetic Products, Nutrition and Allergies (2017). Dietary Reference Values for riboflavin. EFSA J..

[306] Revuelta J.L., Ledesma-Amaro R., Jiménez A. (2016). Industrial production of vitamin B2 by microbial fermentation. Industrial Biotechnology of Vitamins, Biopigments, and Antioxidants.

[307] Mosegaard S., Dipace G., Bross P., Carlsen J., Gregersen N., Olsen R.K.J. (2020). Riboflavin Deficiency-Implications for General Human Health and Inborn Errors of Metabolism. Int. J. Mol. Sci..

[308] Agarwal S., Fulgoni Iii V.L. (2021). Nutritional impact of adding a serving of mushrooms to USDA Food Patterns-a dietary modeling analysis. Food Nutr. Res..

[309] Škrovánková S., Sikorová P. (2010). Vitamin B2 (riboflavin) content in cereal products. Acta Univ. Agric. Silvic. Mendel. Brun..

[310] Vidal-Valverde C., Prodanov M., Sierra I. (1997). Natural fermentation of lentils. Z. Lebensm. Unters. Forsch..

[311] Melse-Boonstra A. (2020). Bioavailability of Micronutrients From Nutrient-Dense Whole Foods: Zooming in on Dairy, Vegetables, and Fruits. Front. Nutr..

[312] Kanno C., Kanehara N., Shirafuji K., Tanji R., Imai T. (1991). Binding form of vitamin B2 in bovine milk: Its concentration, distribution and binding linkage. J. Nutr. Sci. Vitam..

[313] Thielecke F., Lecerf J.M., Nugent A.P. (2021). Processing in the food chain: Do cereals have to be processed to add value to the human diet?. Nutr. Res. Rev..

[314] Pinheiro-Sant’Ana H.M., Stringheta P.C.P., Penteado M.V., Brandão S.C. (1999). Stability of B-vitamins in meats prepared by foodservice. 2.Riboflavin. Foodserv. Res. Int..

[315] Guneser O., Karagul Yuceer Y. (2012). Effect of ultraviolet light on water- and fat-soluble vitamins in cow and goat milk. J. Dairy Sci..

[316] Asadullah, Khair-un-nisa, Tarar O.M., Ali S.A., Jamil K., Begum A. (2010). Study to evaluate the impact of heat treatment on water soluble vitamins in milk. J. Pak. Med. Assoc..

[317] Golbach J.L., Ricke S.C., O’Bryan C.A., Crandall P.G. (2014). Riboflavin in nutrition, food processing, and analysis-A Review. J. Food Res..

[318] Sharabi S., Okun Z., Shpigelman A. (2018). Changes in the shelf life stability of riboflavin, vitamin C and antioxidant properties of milk after (ultra) high pressure homogenization: Direct and indirect effects. Innov. Food Sci. Emerg. Technol..

[319] Allen C., Parks O.W. (1979). Photodegradation of riboflavin in milks exposed to fluorescent light. J. Dairy Sci..

[320] Dror D.K., Allen L.H. (2018). Overview of Nutrients in Human Milk. Adv. Nutr..

[321] Bates C.J., Liu D.S., Fuller N.J., Lucas A. (1985). Susceptibility of riboflavin and vitamin A in breast milk to photodegradation and its implications for the use of banked breast milk in infant feeding. Acta Paediatr. Scand..

[322] Lima H.K., Vogel K., Hampel D., Wagner-Gillespie M., Fogleman A.D. (2020). The Associations Between Light Exposure During Pumping and Holder Pasteurization and the Macronutrient and Vitamin Concentrations in Human Milk. J. Hum. Lact..

[323] Rico D., Penas E., Garcia M.D.C., Martinez-Villaluenga C., Rai D.K., Birsan R.I., Frias J., Martin-Diana A.B. (2020). Sprouted Barley Flour as a Nutritious and Functional Ingredient. Foods.

[324] Tishler M., Pfister K., Babson R.D., Ladenburg K., Fleming A.J. (1947). The reaction between o-aminoazo compounds and barbituric acid; a new synthesis of riboflavin. J. Am. Chem. Soc..

[325] Tischler M., Wellman J.W., Ladenburg K. (1945). The preparation of riboflavin; the synthesis of alloxazines and isoalloxazines. J. Am. Chem. Soc..

[326] Liu S., Hu W., Wang Z., Chen T. (2020). Production of riboflavin and related cofactors by biotechnological processes. Microb. Cell Fact..

[327] Revuelta J.L., Buey R.M., Ledesma-Amaro R., Vandamme E.J. (2016). Microbial biotechnology for the synthesis of (pro)vitamins, biopigments and antioxidants: Challenges and opportunities. Microb. Biotechnol..

[328] Perkins J.B., Sloma A., Hermann T., Theriault K., Zachgo E., Erdenberger T., Hannett N., Chatterjee N.P., Williams V., Rufo G.A. (1999). Genetic engineering of Bacillus subtilis for the commercial production of riboflavin. J. Ind. Microbiol. Biotechnol..

[329] Aguiar T.Q., Silva R., Domingues L. (2015). Ashbya gossypii beyond industrial riboflavin production: A historical perspective and emerging biotechnological applications. Biotechnol. Adv..

[330] Man Z.W., Rao Z.M., Cheng Y.P., Yang T.W., Zhang X., Xu M.J., Xu Z.H. (2014). Enhanced riboflavin production by recombinant Bacillus subtilis RF1 through the optimization of agitation speed. World J. Microbiol. Biotechnol..

[331] Stahmann K.P., Revuelta J.L., Seulberger H. (2000). Three biotechnical processes using *Ashbya gossypii*, *Candida famata*, or *Bacillus subtilis* compete with chemical riboflavin production. Appl. Microbiol. Biotechnol..

[332] Shi T., Wang Y., Wang Z., Wang G., Liu D., Fu J., Chen T., Zhao X. (2014). Deregulation of purine pathway in *Bacillus subtilis* and its use in riboflavin biosynthesis. Microb. Cell Fact..

[333] Abbas C.A., Sibirny A.A. (2011). Genetic control of biosynthesis and transport of riboflavin and flavin nucleotides and construction of robust biotechnological producers. Microbiol. Mol. Biol. Rev..

[334] Wang G., Shi T., Chen T., Wang X., Wang Y., Liu D., Guo J., Fu J., Feng L., Wang Z. (2018). Integrated whole-genome and transcriptome sequence analysis reveals the genetic characteristics of a riboflavin-overproducing *Bacillus subtilis*. Metab. Eng..

[335] Averianova L.A., Balabanova L.A., Son O.M., Podvolotskaya A.B., Tekutyeva L.A. (2020). Production of Vitamin B2 (Riboflavin) by Microorganisms: An Overview. Front. Bioeng. Biotechnol..

[336] Kato T., Park E.Y. (2012). Riboflavin production by *Ashbya gossypii*. Biotechnol. Lett..

[337] EU Commision E. Commission Directive 2006/125/EC of 5 December 2006 on Processed Cereal-Based Foods and Baby Foods for Infants and Young Children. https://eur-lex.europa.eu/eli/dir/2006/125/oj.

[338] Levit R., Savoy de Giori G., de Moreno de LeBlanc A., LeBlanc J.G. (2021). Recent update on lactic acid bacteria producing riboflavin and folates: Application for food fortification and treatment of intestinal inflammation. J. Appl. Microbiol..

[339] Capozzi V., Russo P., Duenas M.T., Lopez P., Spano G. (2012). Lactic acid bacteria producing B-group vitamins: A great potential for functional cereals products. Appl. Microbiol. Biotechnol..

[340] Thakur K., Tomar S.K., De S. (2016). Lactic acid bacteria as a cell factory for riboflavin production. Microb. Biotechnol..

[341] LeBlanc J.G., Laino J.E., del Valle M.J., Vannini V., Van Sinderen D., Taranto M.P., de Valdez G.F., de Giori G.S., Sesma F. (2011). B-group vitamin production by lactic acid bacteria-current knowledge and potential applications. J. Appl. Microbiol..

[342] LeBlanc J.G., Milani C., de Giori G.S., Sesma F., Van Sinderen D., Ventura M. (2013). Bacteria as vitamin suppliers to their host: A gut microbiota perspective. Curr. Opin. Biotechnol..

[343] Solopova A., Bottacini F., Venturi Degli Esposti E., Amaretti A., Raimondi S., Rossi M., Van Sinderen D. (2020). Riboflavin Biosynthesis and Overproduction by a Derivative of the Human Gut Commensal *Bifidobacterium longum* subsp. infantis ATCC 15697. Front. Microbiol..

[344] Burgess C.M., Smid E.J., Rutten G., van Sinderen D. (2006). A general method for selection of riboflavin-overproducing food grade micro-organisms. Microb. Cell Fact..

[345] del Valle M.J., Laiño J.E., de Giori G.S., LeBlanc J. (2014). Riboflavin producing lactic acid bacteria as a biotechnological strategy to obtain bio-enriched soymilk. Food Res. Int..

[346] Daniel H., Binninger E., Rehner G. (1983). Hydrolysis of FMN and FAD by alkaline phosphatase of the intestinal brush-border membrane. Int. J. Vitam. Nutr. Res..

[347] LeBlanc J.G., Burgess C., Sesma F., de Giori G.S., van Sinderen D. (2005). *Lactococcus lactis* is capable of improving the riboflavin status in deficient rats. Br. J. Nutr..

[348] Kasper H. (1970). Vitamin absorption in the colon. Am. J. Proctol.

[349] Iinuma S. (1955). Synthesis of riboflavin by intestinal bacteria. J. Vitam..

[350] Yonezawa A., Inui K. (2013). Novel riboflavin transporter family RFVT/SLC52: Identification, nomenclature, functional characterization and genetic diseases of RFVT/SLC52. Mol. Asp. Med..

[351] Yonezawa A., Masuda S., Katsura T., Inui K. (2008). Identification and functional characterization of a novel human and rat riboflavin transporter, RFT1. Am. J. Physiol. Cell Physiol..

[352] Yao Y., Yonezawa A., Yoshimatsu H., Masuda S., Katsura T., Inui K. (2010). Identification and comparative functional characterization of a new human riboflavin transporter hRFT3 expressed in the brain. J. Nutr..

[353] Yamamoto S., Inoue K., Ohta K.Y., Fukatsu R., Maeda J.Y., Yoshida Y., Yuasa H. (2009). Identification and functional characterization of rat riboflavin transporter 2. J. Biochem..

[354] Jaeger B., Bosch A.M. (2016). Clinical presentation and outcome of riboflavin transporter deficiency: Mini review after five years of experience. J. Inherit. Metab. Dis..

[355] Barile M., Giancaspero T.A., Leone P., Galluccio M., Indiveri C. (2016). Riboflavin transport and metabolism in humans. J. Inherit. Metab. Dis..

[356] Hustad S., McKinley M.C., McNulty H., Schneede J., Strain J.J., Scott J.M., Ueland P.M. (2002). Riboflavin, flavin mononucleotide, and flavin adenine dinucleotide in human plasma and erythrocytes at baseline and after low-dose riboflavin supplementation. Clin. Chem..

[357] Frago S., Martinez-Julvez M., Serrano A., Medina M. (2008). Structural analysis of FAD synthetase from *Corynebacterium ammoniagenes*. BMC Microbiol..

[358] Herguedas B., Martinez-Julvez M., Frago S., Medina M., Hermoso J.A. (2010). Oligomeric state in the crystal structure of modular FAD synthetase provides insights into its sequential catalysis in prokaryotes. J. Mol. Biol..

[359] Barile M., Giancaspero T.A., Brizio C., Panebianco C., Indiveri C., Galluccio M., Vergani L., Eberini I., Gianazza E. (2013). Biosynthesis of flavin cofactors in man: Implications in health and disease. Curr. Pharm. Des..

[360] Serrano A., Ferreira P., Martinez-Julvez M., Medina M. (2013). The prokaryotic FAD synthetase family: A potential drug target. Curr. Pharm. Des..

[361] Chastain J.L., McCormick D.B. (1987). Flavin catabolites: Identification and quantitation in human urine. Am. J. Clin. Nutr..

[362] Lienhart W.D., Gudipati V., Macheroux P. (2013). The human flavoproteome. Arch. Biochem. Biophys..

[363] Macheroux P., Kappes B., Ealick S.E. (2011). Flavogenomics–A genomic and structural view of flavin-dependent proteins. FEBS J..

[364] Singal A.K., Anderson K.E., Adam M.P., Ardinger H.H., Pagon R.A., Wallace S.E., Bean L.J.H., Mirzaa G., Amemiya A. (1993). Variegate Porphyria. GeneReviews^®^.

[365] Musayev F.N., Di Salvo M.L., Saavedra M.A., Contestabile R., Ghatge M.S., Haynes A., Schirch V., Safo M.K. (2009). Molecular basis of reduced pyridoxine 5′-phosphate oxidase catalytic activity in neonatal epileptic encephalopathy disorder. J. Biol. Chem..

[366] Manoj N., Ealick S.E. (2003). Unusual space-group pseudosymmetry in crystals of human phosphopantothenoylcysteine decarboxylase. Acta Cryst. D Biol. Cryst..

[367] Di Meo I., Carecchio M., Tiranti V. (2019). Inborn errors of coenzyme A metabolism and neurodegeneration. J. Inherit. Metab. Dis..

[368] Heeringa S.F., Chernin G., Chaki M., Zhou W., Sloan A.J., Ji Z., Xie L.X., Salviati L., Hurd T.W., Vega-Warner V. (2011). COQ6 mutations in human patients produce nephrotic syndrome with sensorineural deafness. J. Clin. Investig..

[369] Acosta M.J., Vazquez Fonseca L., Desbats M.A., Cerqua C., Zordan R., Trevisson E., Salviati L. (2016). Coenzyme Q biosynthesis in health and disease. Biochim. Biophys. Acta.

[370] Cao Q., Li G.M., Xu H., Shen Q., Sun L., Fang X.Y., Liu H.M., Guo W., Zhai Y.H., Wu B.B. (2017). Coenzyme Q(10) treatment for one child with COQ6 gene mutation induced nephrotic syndrome and literature review. Zhonghua Er Ke Za Zhi.

[371] Afink G., Kulik W., Overmars H., de Randamie J., Veenboer T., van Cruchten A., Craen M., Ris-Stalpers C. (2008). Molecular characterization of iodotyrosine dehalogenase deficiency in patients with hypothyroidism. J. Clin. Endocrinol. Metab..

[372] Friedman J.E., Watson J.A., Lam D.W., Rokita S.E. (2006). Iodotyrosine deiodinase is the first mammalian member of the NADH oxidase/flavin reductase superfamily. J. Biol. Chem..

[373] Moreno J.C., Klootwijk W., Van Toor H., Pinto G., D’Alessandro M., Leger A., Goudie D., Polak M., Gruters A., Visser T.J. (2008). Mutations in the iodotyrosine deiodinase gene and hypothyroidism. N. Engl. J. Med..

[374] O’Brien M.M., Kiely M., Harrington K.E., Robson P.J., Strain J.J., Flynn A. (2001). The North/South Ireland Food Consumption Survey: Vitamin intakes in 18–64-year-old adults. Public Health Nutr..

[375] Thakur K., Tomar S.K., Singh A.K., Mandal S., Arora S. (2017). Riboflavin and health: A review of recent human research. Crit. Rev. Food Sci. Nutr..

[376] Hoppel C.L., Tandler B. (1975). Riboflavin and mouse hepatic cell structure and function. Mitochondrial oxidative metabolism in severe deficiency states. J. Nutr..

[377] Mushtaq S., Su H., Hill M.H., Powers H.J. (2009). Erythrocyte pyridoxamine phosphate oxidase activity: A potential biomarker of riboflavin status?. Am. J. Clin. Nutr..

[378] Grunert S.C. (2014). Clinical and genetical heterogeneity of late-onset multiple acyl-coenzyme A dehydrogenase deficiency. Orphanet J. Rare Dis..

[379] Balasubramaniam S., Christodoulou J., Rahman S. (2019). Disorders of riboflavin metabolism. J. Inherit. Metab. Dis..

[380] O’Callaghan B., Bosch A.M., Houlden H. (2019). An update on the genetics, clinical presentation, and pathomechanisms of human riboflavin transporter deficiency. J. Inherit. Metab. Dis..

[381] Hellebrekers D., Sallevelt S., Theunissen T.E.J., Hendrickx A.T.M., Gottschalk R.W., Hoeijmakers J.G.J., Habets D.D., Bierau J., Schoonderwoerd K.G., Smeets H.J.M. (2017). Novel SLC25A32 mutation in a patient with a severe neuromuscular phenotype. Eur. J. Hum. Genet..

[382] Schiff M., Veauville-Merllie A., Su C.H., Tzagoloff A., Rak M., Ogier de Baulny H., Boutron A., Smedts-Walters H., Romero N.B., Rigal O. (2016). SLC25A32 Mutations and Riboflavin-Responsive Exercise Intolerance. N. Engl. J. Med..

[383] Thompson D.F., Saluja H.S. (2017). Prophylaxis of migraine headaches with riboflavin: A systematic review. J. Clin. Pharmacol. Ther..

[384] Namazi N., Heshmati J., Tarighat-Esfanjani A. (2015). Supplementation with Riboflavin (Vitamin B2) for Migraine Prophylaxis in Adults and Children: A Review. Int. J. Vitam. Nutr. Res..

[385] Tripathi A.K., Dwivedi A., Pal M.K., Rastogi N., Gupta P., Ali S., Prabhu M.B., Kushwaha H.N., Ray R.S., Singh S.K. (2014). Attenuated neuroprotective effect of riboflavin under UV-B irradiation via miR-203/c-Jun signaling pathway in vivo and in vitro. J. Biomed. Sci..

[386] Barbre A.B., Hoane M.R. (2006). Magnesium and riboflavin combination therapy following cortical contusion injury in the rat. Brain Res. Bull..

[387] Seekamp A., Hultquist D.E., Till G.O. (1999). Protection by vitamin B2 against oxidant-mediated acute lung injury. Inflammation.

[388] Mack C.P., Hultquist D.E., Shlafer M. (1995). Myocardial flavin reductase and riboflavin: A potential role in decreasing reoxygenation injury. Biochem. Biophys. Res. Commun..

[389] Suwannasom N., Kao I., Pruss A., Georgieva R., Baumler H. (2020). Riboflavin: The Health Benefits of a Forgotten Natural Vitamin. Int. J. Mol. Sci..

[390] George B.O., Ojegbemi O. (2009). Oxidative stress and the effect of riboflavin supplementation in individuals with uncomplicated malaria infection. Afr. J. Biotechnol..

[391] Akompong T., Ghori N., Haldar K. (2000). In vitro activity of riboflavin against the human malaria parasite *Plasmodium falciparum*. Antimicrob. Agents Chemother..

[392] Araki S., Suzuki M., Fujimoto M., Kimura M. (1995). Enhancement of resistance to bacterial infection in mice by vitamin B2. J. Vet. Med. Sci..

[393] Mazur-Bialy A.I., Buchala B., Plytycz B. (2013). Riboflavin deprivation inhibits macrophage viability and activity-a study on the RAW 264.7 cell line. Br. J. Nutr..

[394] Bertollo C.M., Oliveira A.C., Rocha L.T., Costa K.A., Nascimento E.B., Coelho M.M. (2006). Characterization of the antinociceptive and anti-inflammatory activities of riboflavin in different experimental models. Eur. J. Pharmacol..

[395] Buehler B.A. (2011). Vitamin B2: Riboflavin. J. Evid. Based Integr. Med..

[396] Mazzotta C., Caragiuli S., Caporossi A., Preedy V.R. (2014). Riboflavin and the Cornea and Implications for Cataracts. Handbook of Nutrition, Diet and the Eye.

[397] Chocano-Bedoya P.O., Manson J.E., Hankinson S.E., Willett W.C., Johnson S.R., Chasan-Taber L., Ronnenberg A.G., Bigelow C., Bertone-Johnson E.R. (2011). Dietary B vitamin intake and incident premenstrual syndrome. Am. J. Clin. Nutr..

[398] Alam M.M., Iqbal S., Naseem I. (2015). Ameliorative effect of riboflavin on hyperglycemia, oxidative stress and DNA damage in type-2 diabetic mice: Mechanistic and therapeutic strategies. Arch. Biochem. Biophys..

[399] Schoenen J., Lenaerts M., Bastings E. (1994). High-dose riboflavin as a prophylactic treatment of migraine: Results of an open pilot study. Cephalalgia.

[400] MacLennan S.C., Wade F.M., Forrest K.M., Ratanayake P.D., Fagan E., Antony J. (2008). High-dose riboflavin for migraine prophylaxis in children: A double-blind, randomized, placebo-controlled trial. J. Child Neurol..

[401] Pinto J.T., Rivlin R.S. (1987). Drugs that promote renal excretion of riboflavin. Drug Nutr. Interact..

[402] Pinto J., Huang Y.P., McConnell R.J., Rivlin R.S. (1978). Increased urinary riboflavin excretion resulting from boric acid ingestion. J. Lab. Clin. Med..

[403] Ogura R., Ueta H., Hino Y., Hidaka T., Sugiyama M. (1991). Riboflavin deficiency caused by treatment with adriamycin. J. Nutr. Sci. Vitam..

[404] Pinto J.T., Delman B.N., Dutta P., Nisselbaum J. (1990). Adriamycin-induced increase in serum aldosterone levels: Effects in riboflavin-sufficient and riboflavin-deficient rats. Endocrinology.

[405] Pinto J., Wolinsky M., Rivlin R.S. (1979). Chlorpromazine antagonism of thyroxine-induced flavin formation. Biochem. Pharmacol..

[406] Rivlin R.S., Langdon R.G. (1969). Effects of thyroxine upon biosynthesis of flavin mononucleotide and flavin adenine dinucleotide. Endocrinology.

[407] Pinto J., Huang Y.P., Rivlin R.S. (1981). Inhibition of riboflavin metabolism in rat tissues by chlorpromazine, imipramine, and amitriptyline. J. Clin. Investig..

[408] Rivlin R.S., Menendez C., Langdon R.G. (1968). Biochemical similarities between hypothyroidism and riboflavin deficiency. Endocrinology.

[409] Pelliccione N., Pinto J., Huang Y.P., Rivlin R.S. (1983). Accelerated development of riboflavin deficiency by treatment with chlorpromazine. Biochem. Pharmacol..

[410] Lee S.S., McCormick D.B. (1985). Thyroid hormone regulation of flavocoenzyme biosynthesis. Arch. Biochem. Biophys..

[411] Pinto J., Huang Y.P., Pelliccione N., Rivlin R.S. (1982). Cardiac sensitivity to the inhibitory effects of chlorpromazine, imipramine and amitriptyline upon formation of flavins. Biochem. Pharmacol..

[412] Institute of Medicine (US) Standing Committee on the Scientific Evaluation of Dietary Reference Intakes and its Panel on Folate, Other B Vitamins, and Choline (1998). Dietary Reference Intakes for Thiamin, Riboflavin, Niacin, Vitamin B6, Folate, Vitamin B12, Pantothenic Acid, Biotin, and Choline.

[413] Ross A.C., Caballero B., Cousins R.J., Tucker K.L., Ziegler T.R. (2012). Modern Nutrition in Health and Disease.

[414] Erdman J.W., MacDonald I.A., Zeisel S.H., Penberthy WT K.J. (2012). Present Knowledge in Nutrition.

[415] Berry Ottaway P., Skibsted L.H., Risbo J., Andersen M.L. (2010). Stability of vitamins during food processing and storage. Chemical Deterioration and Physical Instability of Food and Beverages.

[416] World Health Organization (2000). Pellagra and Its Prevention and Control in Major Emergencies.

[417] Bhalla T.C., Vandamme E.J., Revuelta J.L. (2016). Vitamin B3, Niacin. Industrial Biotechnology of Vitamins, Biopigments, and Antioxidants.

[418] Allen L., Benoist B., Dary O., Hurrell R. (2006). WHO/FAO Guidelines on Food Fortification with Micronutrients.

[419] Gazzaniga F., Stebbins R., Chang S.Z., McPeek M.A., Brenner C. (2009). Microbial NAD metabolism: Lessons from comparative genomics. Microbiol. Mol. Biol. Rev..

[420] Li Y.F., Bao W.G. (2007). Why do some yeast species require niacin for growth? Different modes of NAD synthesis. FEMS Yeast Res..

[421] Kurnasov O., Goral V., Colabroy K., Gerdes S., Anantha S., Osterman A., Begley T.P. (2003). NAD biosynthesis: Identification of the tryptophan to quinolinate pathway in bacteria. Chem. Biol..

[422] Noctor G., Hager J., Li S., Rébeillé F., Douce R. (2011). Biosynthesis of NAD and Its Manipulation in Plants. Advances in Botanical Research.

[423] Magnusdottir S., Ravcheev D., de Crecy-Lagard V., Thiele I. (2015). Systematic genome assessment of B-vitamin biosynthesis suggests co-operation among gut microbes. Front. Genet..

[424] Kirkland J.B., Meyer-Ficca M.L. (2018). Chapter Three-Niacin. Adv. Food. Nutr. Res..

[425] Bauer J.E. (1998). Nutritional uniqueness of cats. Vet. Q.

[426] Reeds P.J. (2000). Dispensable and indispensable amino acids for humans. J. Nutr..

[427] Shibata K. (2018). Organ Co-Relationship in Tryptophan Metabolism and Factors That Govern the Biosynthesis of Nicotinamide from Tryptophan. J. Nutr. Sci. Vitam..

[428] Gasperi V., Sibilano M., Savini I., Catani M.V. (2019). Niacin in the Central Nervous System: An Update of Biological Aspects and Clinical Applications. Int. J. Mol. Sci..

[429] Murray M.F. (2003). Tryptophan depletion and HIV infection: A metabolic link to pathogenesis. Lancet Infect. Dis..

[430] Fukuwatari T., Shibata K. (2013). Nutritional aspect of tryptophan metabolism. Int. J. Tryptophan Res..

[431] Meir Z., Osherov N. (2018). Vitamin Biosynthesis as an Antifungal Target. J. Fungi.

[432] EFSA (2017). Dietary Reference Values for Nutrients Summary report. EFSA Support. Publ..

[433] Food and Drug Administration Converting Units of Measure for Folate, Niacin, and Vitamins A, D, and E on the Nutrition and Supplement Facts Labels: Guidance for Industry. https://www.fda.gov/regulatory-information/search-fda-guidance-documents/guidance-industry-converting-units-measure-folate-niacin-and-vitamins-d-and-e-nutrition-and.

[434] Fukuwatari T., Ohta M., Kimtjra N., Sasaki R., Shibata K. (2004). Conversion ratio of tryptophan to niacin in Japanese women fed a purified diet conforming to the Japanese Dietary Reference Intakes. J. Nutr. Sci. Vitam..

[435] Combs G.F., McClung J.P. (2017). Niacin. The Vitamins.

[436] Lanska D.J. (2012). The discovery of niacin, biotin, and pantothenic acid. Ann. Nutr. Metab..

[437] Henderson L.M., Koski R.E., D’Angeli F. (1955). The role of riboflavin and vitamin B6 in tryptophan metabolism. J. Biol. Chem..

[438] Shibata K., Mushiage M., Kondo T., Hayakawa T., Tsuge H. (1995). Effects of vitamin B6 deficiency on the conversion ratio of tryptophan to niacin. Biosci. Biotechnol. Biochem..

[439] Shibata K., Kobayashi R., Fukuwatari T. (2015). Vitamin B1 deficiency inhibits the increased conversion of tryptophan to nicotinamide in severe food-restricted rats. Biosci. Biotechnol. Biochem..

[440] Fukuwatari T., Shibata K. (2007). Effect of nicotinamide administration on the tryptophan-nicotinamide pathway in humans. Int. J. Vitam. Nutr. Res..

[441] Lule V.K., Garg S., Gosewade S.C., Tomar S.K., Khedkar C.D., Caballero B., Finglas P.M., Toldrá F. (2016). Niacin. Encyclopedia of Food and Health.

[442] Wall J.S., Carpenter K.J. (1988). Variation in Availability of Niacin in Grain Products. Food Technol..

[443] Blum R. (2020). Vitamins, 8. Vitamin B3 (Niacin). Ullmann’s Encyclopedia of Industrial Chemistry.

[444] EFSA (2014). Scientific opinion on dietary reference values for niacin. EFSA J..

[445] Chawla J., Kvarnberg D., Biller J., Ferro J.M. (2014). Chapter 59—Hydrosoluble vitamins. Handbook of Clinical Neurology.

[446] Raman J., Jang K.Y., Oh Y.L., Oh M., Im J.H., Lakshmanan H., Sabaratnam V. (2020). Cultivation and Nutritional Value of Prominent *Pleurotus spp*.: An Overview. Mycobiology.

[447] Kumar K. (2020). Nutraceutical Potential and Processing Aspects of Oyster Mushrooms (*Pleurotus* Species). Curr. Nutr. Food Sci..

[448] Çatak J., Yaman M. (2019). Determination of Nicotinic Acid and Nicotinamide Forms of Vitamin B3 (Niacin) in Fruits and Vegetables by HPLC Using Postcolumn Derivatization System. Pak. J. Nutr..

[449] Prousky J., Millman C.G., Kirkland J.B. (2011). Pharmacologic Use of Niacin. J. Evid. Based Integr. Med..

[450] Angelino D., Tassotti M., Brighenti F., Del Rio D., Mena P. (2018). Niacin, alkaloids and (poly)phenolic compounds in the most widespread Italian capsule-brewed coffees. Sci. Rep..

[451] Lang R., Yagar E.F., Eggers R., Hofmann T. (2008). Quantitative investigation of trigonelline, nicotinic acid, and nicotinamide in foods, urine, and plasma by means of LC-MS/MS and stable isotope dilution analysis. J. Agric. Food Chem..

[452] Carvalho A. (1962). Variability of the Niacin Content in Coffee. Nature.

[453] Stadler R.H., Varga N., Hau J., Vera F.A., Welti D.H. (2002). Alkylpyridiniums. 1. Formation in model systems via thermal degradation of trigonelline. J. Agric. Food Chem..

[454] Kremer J.I., Gompel K., Bakuradze T., Eisenbrand G., Richling E. (2018). Urinary Excretion of Niacin Metabolites in Humans After Coffee Consumption. Mol. Nutr. Food Res..

[455] Ghafoorunissa, Rao B.S. (1973). Effect of leucine on enzymes of the tryptophan-niacin metabolic pathway in rat liver and kidney. Biochem. J..

[456] Badawy A.A., Lake S.L., Dougherty D.M. (2014). Mechanisms of the pellagragenic effect of leucine: Stimulation of hepatic tryptophan oxidation by administration of branched-chain amino acids to healthy human volunteers and the role of plasma free tryptophan and total kynurenines. Int. J. Tryptophan Res..

[457] Badawy A.A. (2017). Kynurenine Pathway of Tryptophan Metabolism: Regulatory and Functional Aspects. Int. J. Tryptophan Res..

[458] Katz S.H., Hediger M.L., Valleroy L.A. (1974). Traditional maize processing techniques in the new world. Science.

[459] Bender D.A. (1983). Effects of a dietary excess of leucine on the metabolism of tryptophan in the rat: A mechanism for the pellagragenic action of leucine. Br. J. Nutr..

[460] Salter M., Bender D.A., Pogson C.I. (1985). Leucine and tryptophan metabolism in rats. Biochem. J..

[461] Bates C.J., Caballero B. (2013). Niacin and Pellagra. Encyclopedia of Human Nutrition.

[462] Cook N.E., Carpenter K.J. (1987). Leucine excess and niacin status in rats. J. Nutr..

[463] Manson J.A., Carpenter K.J. (1978). The effect of a high level of dietary leucine on the niacin status of dogs. J. Nutr..

[464] Hegedus M., Pedersen B., Eggum B.O. (1985). The influence of milling on the nutritive value of flour from cereal grains. 7. Vitamins and tryptophan. Plant Foods Hum. Nutr..

[465] Chamlagain B., Rautio S., Edelmann M., Ollilainen V., Piironen V. (2020). Niacin contents of cereal-milling products in food-composition databases need to be updated. J. Food Compos. Anal..

[466] Çatak J. (2019). Determination of niacin profiles in some animal and plant based foods by high performance liquid chromatography: Association with healthy nutrition. J. Anim. Sci. Technol..

[467] Saleh A.S.M., Wang P., Wang N., Yang L., Xiao Z. (2019). Brown Rice Versus White Rice: Nutritional Quality, Potential Health Benefits, Development of Food Products, and Preservation Technologies. Compr. Rev. Food Sci. Food Saf..

[468] Adebo O.A. (2020). African Sorghum-Based Fermented Foods: Past, Current and Future Prospects. Nutrients.

[469] Wyness L. (2016). The role of red meat in the diet: Nutrition and health benefits. Proc. Nutr. Soc..

[470] Feeney M.J., Dwyer J., Hasler-Lewis C.M., Milner J.A., Noakes M., Rowe S., Wach M., Beelman R.B., Caldwell J., Cantorna M.T. (2014). Mushrooms and Health Summit proceedings. J. Nutr..

[471] Muehlhoff E., Bennett A., McMahon D. (2013). Milk and Dairy Products in Human Nutrition.

[472] Biesalksi H.K., Back E.I., Roginski H. (2002). VITAMINS|Niacin, Nutritional Significance. Encyclopedia of Dairy Sciences.

[473] Satya S., Kaushik G., Naik S.N. (2010). Processing of food legumes: A boon to human nutrition. Med. J. Nutr. Metab..

[474] Sobral M.M.C., Cunha S.C., Faria M.A., Ferreira I.M. (2018). Domestic Cooking of Muscle Foods: Impact on Composition of Nutrients and Contaminants. Compr. Rev. Food Sci. Food Saf..

[475] Pinheiro-Sant’Ana H.M., Penteado M.V.C., Stringheta P.C., Chaves J.B.P. (1999). Stability of B-Vitamins in Meats Prepared by Foodservice. 3. Nicotinic Acid. Foodserv. Res. Int..

[476] Meyer B.H., Hinman W.F., Halliday E.G. (1947). Retention of some vitamins of the B-complex in beef during cooking. Food Res..

[477] Kilcast D. (1994). Effect of Irradiation on Vitamins. Food Chem..

[478] Yaman M., Catak J., Ugur H., Gurbuz M., Belli I., Tanyildiz S.N., Yildirim H., Cengiz S., Yavuz B.B., Kismiroglu C. (2021). The bioaccessibility of water-soluble vitamins: A review. Trends Food Sci. Technol..

[479] Akça S.N., Sargın H.S., Mızrak Ö.F., Yaman M. (2019). Determination and assessment of the bioaccessibility of vitamins B1, B2, and B3 in commercially available cereal-based baby foods. Microchem. J..

[480] Gregory J.F. (2012). Accounting for differences in the bioactivity and bioavailability of vitamers. Food Nutr. Res..

[481] Zaupa M., Scazzina F., Dall’Asta M., Calani L., Del Rio D., Bianchi M.A., Melegari C., De Albertis P., Tribuzio G., Pellegrini N. (2014). In vitro bioaccessibility of phenolics and vitamins from durum wheat aleurone fractions. J. Agric. Food Chem..

[482] Carter E.G., Carpenter K.J. (1982). The bioavailability for humans of bound niacin from wheat bran. Am. J. Clin. Nutr..

[483] Harper A.E., Punekar B.D., Elvehjem C.A. (1958). Effect of alkali treatment on the availability of niacin and amino acids in maize. J. Nutr..

[484] Carpenter K.J. (1983). The relationship of pellagra to corn and the low availability of niacin in cereals. Experientia Suppl..

[485] Kodicek E., Braude R., Kon S.K., Mitchell K.G. (1959). The availability to pigs of nicotinic acid in tortilla baked from maize treated with lime-water. Br. J. Nutr..

[486] Kodicek E., Braude R., Kon S.K., Mitchell K.G. (1956). The effect of alkaline hydrolysis of maize on the availability of its nicotinic acid to the pig. Br. J. Nutr..

[487] Wacher C. Nixtamalization, a Mesoamerican technology to process maize at small-scale with great potential for improving the nutritional quality of maize based foods. Proceedings of the Food-Based Approaches for a Healthy Nutrition.

[488] Escalante-Aburto A., Mariscal-Moreno R.M., Santiago-Ramos D., Ponce-García N. (2020). An Update of Different Nixtamalization Technologies, and Its Effects on Chemical Composition and Nutritional Value of Corn Tortillas. Food Rev. Int..

[489] Salazar R., Arambula-Villa G., Luna-Barcenas G., Figueroa-Cardenas J.D., Azuara E., Vazquez-Landaverde P.A. (2014). Effect of added calcium hydroxide during corn nixtamalization on acrylamide content in tortilla chips. LWT Food Sci. Technol..

[490] Maureen N., Kaaya A.N., Kauffman J., Narrod C., Atukwase A. (2020). Enhancing Nutritional Benefits and Reducing Mycotoxin Contamination of Maize through Nixtamalization. J. Biol. Sci..

[491] Sefa-Dedeh S., Cornelius B., Sakyi-Dawson E., Afoakwa E.O. (2004). Effect of nixtamalization on the chemical and functional properties of maize. Food Chem..

[492] Kamau E.H., Nkhata S.G., Ayua E.O. (2020). Extrusion and nixtamalization conditions influence the magnitude of change in the nutrients and bioactive components of cereals and legumes. Food Sci. Nutr..

[493] de la Parra C., Saldivar S.O., Liu R.H. (2007). Effect of processing on the phytochemical profiles and antioxidant activity of corn for production of masa, tortillas, and tortilla chips. J. Agric. Food Chem..

[494] Schaarschmidt S., Fauhl-Hassek C. (2019). Mycotoxins during the Processes of Nixtamalization and Tortilla Production. Toxins.

[495] FAO (1992). Maize in Human Nutrition.

[496] Bressani R., Paz y Paz R., Scrimshaw N.S. (1958). Corn Nutrient Losses, Chemical Changes in Corn during Preparation of Tortillas. J. Agric. Food Chem..

[497] Carter E.G., Carpenter K.J. (1982). The available niacin values of foods for rats and their relation to analytical values. J. Nutr..

[498] Dunn M.L., Jain V., Klein B.P. (2014). Stability of key micronutrients added to fortified maize flours and corn meal. Ann. N. Y. Acad. Sci..

[499] Laguna J., Carpenter K.J. (1951). Raw versus processed corn in niacin-deficient diets. J. Nutr..

[500] Braham J.E., Villarreal A., Bressani R. (1962). Effect of lime treatment of corn on the availability of niacin for cats. J. Nutr..

[501] Kodicek E., Ashby D.R., Muller M., Carpenter K.J. (1974). The conversion of bound nicotinic acid to free nicotinamide on roasting sweet corn. Proc. Nutr. Soc..

[502] Buckel L., Kremer J.I., Stegmüller S., Richling E. (2019). Fast, Sensitive and Robust Determination of Nicotinic Acid (Vitamin B3) Contents in Coffee Beverages Depending on the Degree of Roasting and Brewing Technique. Proceedings.

[503] Taguchi H., Sakaguchi M., Shimabayashi Y. (1985). Trigonelline Content in Coffee Beans and the Thermal-Conversion of Trigonelline into Nicotinic-Acid during the Roasting of Coffee Beans. Agr. Biol. Chem..

[504] Bressani R., Navarrete D.A. (1959). Niacin Content of Coffee in Central America. J. Food Sci..

[505] Teply L.J., Prier R.F. (1957). Nutrients in Coffee-Nutritional Evaluation of Coffee Including Niacin Bioassay. J. Agric. Food Chem..

[506] Caprioli G., Cortese M., Maggi F., Minnetti C., Odello L., Sagratini G., Vittori S. (2014). Quantification of caffeine, trigonelline and nicotinic acid in espresso coffee: The influence of espresso machines and coffee cultivars. Int. J. Food Sci. Nutr..

[507] Chaturvedi A., Geervani P. (1986). Bioavailability of niacin from processed groundnuts. J. Nutr. Sci. Vitam..

[508] Nurit E., Lyan B., Pujos-Guillot E., Branlard G., Piquet A. (2016). Change in B and E vitamin and lutein, β-sitosterol contents in industrial milling fractions and during toasted bread production. J. Cereal Sci..

[509] Asiedu M., Lied E., Nilsen R., Sandnes K. (1993). Effect of processing (sprouting and/or fermentation) on sorghum and maize: II. Vitamins and amino acid composition. Biological utilization of maize protein. Food Chem..

[510] Žilić S., Basić Z., Hadži-Tašković Šukalović V., Maksimović V., Janković M., Filipović M. (2014). Can the sprouting process applied to wheat improve the contents of vitamins and phenolic compounds and antioxidant capacity of the flour?. Int. J. Food Sci. Technol..

[511] Lay M.M.G., Fields M.L. (1981). Nutritive-Value of Germinated Corn and Corn Fermented after Germination. J. Food Sci..

[512] Mihhalevski A., Nisamedtinov I., Halvin K., Oseka A., Paalme T. (2013). Stability of B-complex vitamins and dietary fiber during rye sourdough bread production. J. Cereal Sci..

[513] Mani I. (2020). Microbial Production of Vitamins.

[514] Kumar S., Babu B.V. (2009). Process Intensification of Nicotinic Acid Production via Enzymatic Conversion using Reactive Extraction. Chem. Biochem. Eng. Q..

[515] Chuck R. (2009). Green Sustainable Chemistry in the Production of Nicotinates. Sustainable Industrial Chemistry.

[516] Chuck R. (2000). A catalytic green process for the production of niacin. Chimia.

[517] Eschenmoser W. (1997). 100 years of progress with LONZA. Chimia.

[518] Chuck R. (2005). Technology development in nicotinate production. Appl. Catal. A-Gen..

[519] Gong J.S., Zhang Q., Gu B.C., Dong T.T., Li H., Li H., Lu Z.M., Shi J.S., Xu Z.H. (2018). Efficient biocatalytic synthesis of nicotinic acid by recombinant nitrilase via high density culture. Bioresour. Technol..

[520] Shaw N.M., Robins K.T., Kiener A. (2003). Lonza: 20 years of biotransformations. Adv. Synth. Catal..

[521] de Carvalho C.C. (2017). Whole cell biocatalysts: Essential workers from Nature to the industry. Microb. Biotechnol..

[522] Wang Z., Liu Z., Cui W., Zhou Z. (2017). Establishment of Bioprocess for Synthesis of Nicotinamide by Recombinant Escherichia coli Expressing High-Molecular-Mass Nitrile Hydratase. Appl. Biochem. Biotechnol..

[523] Prasad S., Raj J., Bhalla T.C. (2007). Bench scale conversion of 3-cyanopyidine to nicotinamide using resting cells of *Rhodococcus rhodochrous* PA-34. Indian J. Microbiol..

[524] Shen J.D., Cai X., Liu Z.Q., Zheng Y.G. (2021). Nitrilase: A promising biocatalyst in industrial applications for green chemistry. Crit. Rev. Biotechnol..

[525] Mathew C.D., Nagasawa T., Kobayashi M., Yamada H. (1988). Nitrilase-Catalyzed Production of Nicotinic Acid from 3-Cyanopyridine in *Rhodococcus rhodochrous* J1. Appl. Environ. Microbiol..

[526] Prasad S., Bhalla T.C. (2010). Nitrile hydratases (NHases): At the interface of academia and industry. Biotechnol. Adv..

[527] Nagasawa T., Mathew C.D., Mauger J., Yamada H. (1988). Nitrile Hydratase-Catalyzed Production of Nicotinamide from 3-Cyanopyridine in Rhodococcus rhodochrous J1. Appl. Environ. Microbiol..

[528] Bhalla T.C., Kumar V., Kumar V., Thakur N., Savitri (2018). Nitrile Metabolizing Enzymes in Biocatalysis and Biotransformation. Appl. Biochem. Biotechnol..

[529] Gong J.S., Lu Z.M., Li H., Shi J.S., Zhou Z.M., Xu Z.H. (2012). Nitrilases in nitrile biocatalysis: Recent progress and forthcoming research. Microb. Cell Fact..

[530] Gong J.S., Shi J.S., Lu Z.M., Li H., Zhou Z.M., Xu Z.H. (2017). Nitrile-converting enzymes as a tool to improve biocatalysis in organic synthesis: Recent insights and promises. Crit. Rev. Biotechnol..

[531] Cheng Z., Xia Y., Zhou Z. (2020). Recent Advances and Promises in Nitrile Hydratase: From Mechanism to Industrial Applications. Front. Bioeng. Biotechnol..

[532] Busch H., Hagedoorn P.L., Hanefeld U. (2019). *Rhodococcus* as a Versatile Biocatalyst in Organic Synthesis. Int. J. Mol. Sci..

[533] Berner L.A., Clydesdale F.M., Douglass J.S. (2001). Fortification contributed greatly to vitamin and mineral intakes in the United States, 1989–1991. J. Nutr..

[534] Muthayya S., Hall J., Bagriansky J., Sugimoto J., Gundry D., Matthias D., Prigge S., Hindle P., Moench-Pfanner R., Maberly G. (2012). Rice fortification: An emerging opportunity to contribute to the elimination of vitamin and mineral deficiency worldwide. Food Nutr. Bull..

[535] Meyer-Ficca M., Kirkland J.B. (2016). Niacin. Adv. Nutr..

[536] De Dios Figueroa Cardenas J., Godinez M.G., Mendez N.L., Guzman A.L., Acosta L.M. (2003). Nutritional quality of nixtamal tortillas fortified with vitamins and soy proteins. Int. J. Food Sci. Nutr..

[537] Shewry P.R., Hawkesford M.J., Piironen V., Lampi A.M., Gebruers K., Boros D., Andersson A.A., Aman P., Rakszegi M., Bedo Z. (2013). Natural variation in grain composition of wheat and related cereals. J. Agric. Food Chem..

[538] Shewry P.R., Van Schaik F., Ravel C., Charmet G., Rakszegi M., Bedo Z., Ward J.L. (2011). Genotype and environment effects on the contents of vitamins B1, B2, B3, and B6 in wheat grain. J. Agric. Food Chem..

[539] Kim G.R., Jung E.S., Lee S., Lim S.H., Ha S.H., Lee C.H. (2014). Combined mass spectrometry-based metabolite profiling of different pigmented rice (*Oryza sativa* L.) seeds and correlation with antioxidant activities. Molecules.

[540] Gerdes S., Lerma-Ortiz C., Frelin O., Seaver S.M., Henry C.S., de Crecy-Lagard V., Hanson A.D. (2012). Plant B vitamin pathways and their compartmentation: A guide for the perplexed. J. Exp. Bot..

[541] Nuss E.T., Tanumihardjo S.A. (2011). Quality protein maize for Africa: Closing the protein inadequacy gap in vulnerable populations. Adv. Nutr..

[542] Prasanna B.M., Vasal S.K., Kassahun B., Singh N.N. (2001). Quality protein maize. Curr. Sci..

[543] Prasanna B.M., Palacios-Rojas N., Hossain F., Muthusamy V., Menkir A., Dhliwayo T., Ndhlela T., San Vicente F., Nair S.K., Vivek B.S. (2019). Molecular Breeding for Nutritionally Enriched Maize: Status and Prospects. Front. Genet..

[544] Goredema-Matongera N., Ndhlela T., Magorokosho C., Kamutando C.N., Van Biljon A., Labuschagne M. (2021). Multinutrient Biofortification of Maize (*Zea mays* L.) in Africa: Current Status, Opportunities and Limitations. Nutrients.

[545] Maqbool M.A., Issa A.B., Khokhar E.S. (2021). Quality protein maize (QPM): Importance, genetics, timeline of different events, breeding strategies and varietal adoption. Plant Breed..

[546] Bhat J.S., Patil B.S., Hariprasanna K., Hossain F., Muthusamy V., Mukri G., Mallikarjuna M.G., Zunjare R., Singh S.P., Sankar S.M. (2018). Genetic Enhancement of Micronutrient Content in Cereals. SABRAO J. Breed. Genet..

[547] Coates P.M., Betz J.M., Blackman M.R., Cragg G.M., Levine M., Moss J., White J.D. (2010). Encyclopedia of Dietary Supplements.

[548] Bechgaard H., Jespersen S. (1977). GI absorption of niacin in humans. J. Pharm. Sci..

[549] Lan S.J., Henderson L.M. (1968). Uptake of nicotinic acid and nicotinamide by rat erythrocytes. J. Biol. Chem..

[550] Revollo J.R., Grimm A.A., Imai S. (2004). The NAD biosynthesis pathway mediated by nicotinamide phosphoribosyltransferase regulates Sir2 activity in mammalian cells. J. Biol. Chem..

[551] Van der Veer E., Ho C., O’Neil C., Barbosa N., Scott R., Cregan S.P., Pickering J.G. (2007). Extension of human cell lifespan by nicotinamide phosphoribosyltransferase. J. Biol. Chem..

[552] Ramsey K.M., Yoshino J., Brace C.S., Abrassart D., Kobayashi Y., Marcheva B., Hong H.K., Chong J.L., Buhr E.D., Lee C. (2009). Circadian clock feedback cycle through NAMPT-mediated NAD+ biosynthesis. Science.

[553] Savitz J. (2020). The kynurenine pathway: A finger in every pie. Mol. Psychiatry.

[554] Li R., Yu K., Wang Q., Wang L., Mao J., Qian J. (2016). Pellagra Secondary to Medication and Alcoholism: A Case Report and Review of the Literature. Nutr. Clin. Pract..

[555] Yang H., Yang T., Baur J.A., Perez E., Matsui T., Carmona J.J., Lamming D.W., Souza-Pinto N.C., Bohr V.A., Rosenzweig A. (2007). Nutrient-sensitive mitochondrial NAD+ levels dictate cell survival. Cell.

[556] Agledal L., Niere M., Ziegler M. (2010). The phosphate makes a difference: Cellular functions of NADP. Redox Rep..

[557] Piepho R.W. (2000). The pharmacokinetics and pharmacodynamics of agents proven to raise high-density lipoprotein cholesterol. Am. J. Cardiol..

[558] Pieper J.A. (2003). Overview of niacin formulations: Differences in pharmacokinetics, efficacy, and safety. Am. J. Health Syst. Pharm..

[559] Wilkin J.K., Wilkin O., Kapp R., Donachie R., Chernosky M.E., Buckner J. (1982). Aspirin blocks nicotinic acid-induced flushing. Clin. Pharmacol. Ther..

[560] Lenglet A., Liabeuf S., Bodeau S., Louvet L., Mary A., Boullier A., Lemaire-Hurtel A.S., Jonet A., Sonnet P., Kamel S. (2016). N-methyl-2-pyridone-5-carboxamide (2PY)-Major Metabolite of Nicotinamide: An Update on an Old Uremic Toxin. Toxins.

[561] Breen L.T., Smyth L.M., Yamboliev I.A., Mutafova-Yambolieva V.N. (2006). beta-NAD is a novel nucleotide released on stimulation of nerve terminals in human urinary bladder detrusor muscle. Am. J. Physiol. Ren. Physiol..

[562] Mutafova-Yambolieva V.N. (2012). Neuronal and extraneuronal release of ATP and NAD(+) in smooth muscle. IUBMB Life.

[563] Gruenbacher G., Gander H., Rahm A., Dobler G., Drasche A., Troppmair J., Nussbaumer W., Thurnher M. (2019). The Human G Protein-Coupled ATP Receptor P2Y11 Is Associated With IL-10 Driven Macrophage Differentiation. Front. Immunol..

[564] Durnin L., Dai Y., Aiba I., Shuttleworth C.W., Yamboliev I.A., Mutafova-Yambolieva V.N. (2012). Release, neuronal effects and removal of extracellular beta-nicotinamide adenine dinucleotide (beta-NAD(+)) in the rat brain. Eur. J. Neurosci..

[565] Durnin L., Kurahashi M., Sanders K.M., Mutafova-Yambolieva V.N. (2020). Extracellular metabolism of the enteric inhibitory neurotransmitter beta-nicotinamide adenine dinucleotide (beta-NAD) in the murine colon. J. Physiol..

[566] Umapathy N.S., Zemskov E.A., Gonzales J., Gorshkov B.A., Sridhar S., Chakraborty T., Lucas R., Verin A.D. (2010). Extracellular beta-nicotinamide adenine dinucleotide (beta-NAD) promotes the endothelial cell barrier integrity via PKA- and EPAC1/Rac1-dependent actin cytoskeleton rearrangement. J. Cell Physiol..

[567] Hiller S.D., Heldmann S., Richter K., Jurastow I., Kullmar M., Hecker A., Wilker S., Fuchs-Moll G., Manzini I., Schmalzing G. (2018). beta-Nicotinamide Adenine Dinucleotide (beta-NAD) Inhibits ATP-Dependent IL-1beta Release from Human Monocytic Cells. Int. J. Mol. Sci..

[568] Nikiforov A., Kulikova V., Ziegler M. (2015). The human NAD metabolome: Functions, metabolism and compartmentalization. Crit. Rev. Biochem. Mol. Biol..

[569] Pollak N., Dolle C., Ziegler M. (2007). The power to reduce: Pyridine nucleotides—Small molecules with a multitude of functions. Biochem. J..

[570] Edenberg H.J. (2007). The genetics of alcohol metabolism: Role of alcohol dehydrogenase and aldehyde dehydrogenase variants. Alcohol Res. Health.

[571] Nakahata Y., Sahar S., Astarita G., Kaluzova M., Sassone-Corsi P. (2009). Circadian control of the NAD+ salvage pathway by CLOCK-SIRT1. Science.

[572] Frederick D.W., Davis J.G., Davila A., Agarwal B., Michan S., Puchowicz M.A., Nakamaru-Ogiso E., Baur J.A. (2015). Increasing NAD synthesis in muscle via nicotinamide phosphoribosyltransferase is not sufficient to promote oxidative metabolism. J. Biol. Chem..

[573] Canto C., Menzies K.J., Auwerx J. (2015). NAD(+) Metabolism and the Control of Energy Homeostasis: A Balancing Act between Mitochondria and the Nucleus. Cell Metab..

[574] Guse A.H., Lee H.C. (2008). NAADP: A universal Ca2+ trigger. Sci. Signal..

[575] Ferrero E., Lo Buono N., Horenstein A.L., Funaro A., Malavasi F. (2014). The ADP-ribosyl cyclases—The current evolutionary state of the ARCs. Front. Biosci..

[576] Partida-Sanchez S., Cockayne D.A., Monard S., Jacobson E.L., Oppenheimer N., Garvy B., Kusser K., Goodrich S., Howard M., Harmsen A. (2001). Cyclic ADP-ribose production by CD38 regulates intracellular calcium release, extracellular calcium influx and chemotaxis in neutrophils and is required for bacterial clearance in vivo. Nat. Med..

[577] Chong A., Malavasi F., Israel S., Khor C.C., Yap V.B., Monakhov M., Chew S.H., Lai P.S., Ebstein R.P. (2017). ADP ribosyl-cyclases (CD38/CD157), social skills and friendship. Psychoneuroendocrinology.

[578] Leung A.K.L. (2017). PARPs. Curr. Biol..

[579] Perina D., Mikoc A., Ahel J., Cetkovic H., Zaja R., Ahel I. (2014). Distribution of protein poly (ADP-ribosyl) ation systems across all domains of life. DNA Repair.

[580] Aravind L., Zhang D., de Souza R.F., Anand S., Iyer L.M. (2015). The natural history of ADP-ribosyltransferases and the ADP-ribosylation system. Curr. Top. Microbiol. Immunol..

[581] Trucco C., Rolli V., Oliver F.J., Flatter E., Masson M., Dantzer F., Niedergang C., Dutrillaux B., Menissier-de Murcia J., de Murcia G. (1999). A dual approach in the study of poly (ADP-ribose) polymerase: In vitro random mutagenesis and generation of deficient mice. Mol. Cell. Biochem..

[582] Shall S., de Murcia G. (2000). Poly (ADP-ribose) polymerase-1: What have we learned from the deficient mouse model?. Mutat. Res..

[583] Herceg Z., Wang Z.Q. (2001). Functions of poly (ADP-ribose) polymerase (PARP) in DNA repair, genomic integrity and cell death. Mutat. Res..

[584] Berger N.A., Besson V.C., Boulares A.H., Burkle A., Chiarugi A., Clark R.S., Curtin N.J., Cuzzocrea S., Dawson T.M., Dawson V.L. (2018). Opportunities for the repurposing of PARP inhibitors for the therapy of non-oncological diseases. Br. J. Pharmacol..

[585] Buisson R., Dion-Cote A.M., Coulombe Y., Launay H., Cai H., Stasiak A.Z., Stasiak A., Xia B., Masson J.Y. (2010). Cooperation of breast cancer proteins PALB2 and piccolo BRCA2 in stimulating homologous recombination. Nat. Struct. Mol. Biol..

[586] Bochum S., Berger S., Martens U.M. (2018). Olaparib. Recent Results Cancer Res..

[587] Lord C.J., Ashworth A. (2017). PARP inhibitors: Synthetic lethality in the clinic. Science.

[588] Palazzo L., Mikoc A., Ahel I. (2017). ADP-ribosylation: New facets of an ancient modification. FEBS J..

[589] Moraes D.S., Moreira D.C., Andrade J.M.O., Santos S.H.S. (2020). Sirtuins, brain and cognition: A review of resveratrol effects. IBRO Rep..

[590] Tanner K.G., Landry J., Sternglanz R., Denu J.M. (2000). Silent information regulator 2 family of NAD- dependent histone/protein deacetylases generates a unique product, 1-*O*-acetyl-ADP-ribose. Proc. Natl. Acad. Sci. USA.

[591] Asher G., Gatfield D., Stratmann M., Reinke H., Dibner C., Kreppel F., Mostoslavsky R., Alt F.W., Schibler U. (2008). SIRT1 regulates circadian clock gene expression through PER2 deacetylation. Cell.

[592] Nakahata Y., Kaluzova M., Grimaldi B., Sahar S., Hirayama J., Chen D., Guarente L.P., Sassone-Corsi P. (2008). The NAD+-dependent deacetylase SIRT1 modulates CLOCK-mediated chromatin remodeling and circadian control. Cell.

[593] Imai S.I., Guarente L. (2016). It takes two to tango: NAD(+) and sirtuins in aging/longevity control. NPJ Aging Mech. Dis..

[594] Jiang Y., Liu J., Chen D., Yan L., Zheng W. (2017). Sirtuin Inhibition: Strategies, Inhibitors, and Therapeutic Potential. Trends Pharmacol. Sci..

[595] Van de Ven R.A.H., Santos D., Haigis M.C. (2017). Mitochondrial Sirtuins and Molecular Mechanisms of Aging. Trends Mol. Med..

[596] Bayele H.K. (2020). Sirtuins transduce STACs signals through steroid hormone receptors. Sci. Rep..

[597] Gaal Z., Csernoch L. (2020). Impact of Sirtuin Enzymes on the Altered Metabolic Phenotype of Malignantly Transformed Cells. Front. Oncol..

[598] Carpenter K.J. (1981). Pellagra.

[599] Prabhu D., Dawe R.S., Mponda K. (2021). Pellagra a review exploring causes and mechanisms, including isoniazid-induced pellagra. Photodermatol. Photoimmunol. Photomed..

[600] Williams A.C., Hill L.J. (2020). The 4 D’s of Pellagra and Progress. Int. J. Tryptophan Res..

[601] Ramirez-Cabral N.Y.Z., Kumar L., Shabani F. (2017). Global alterations in areas of suitability for maize production from climate change and using a mechanistic species distribution model (CLIMEX). Sci. Rep..

[602] Schmid M.A., Salomeyesudas B., Satheesh P., Hanley J., Kuhnlein H.V. (2007). Intervention with traditional food as a major source of energy, protein, iron, vitamin C and vitamin A for rural Dalit mothers and young children in Andhra Pradesh, South India. Asia Pac. J. Clin. Nutr..

[603] Malfait P., Moren A., Dillon J.C., Brodel A., Begkoyian G., Etchegorry M.G., Malenga G., Hakewill P. (1993). An outbreak of pellagra related to changes in dietary niacin among Mozambican refugees in Malawi. Int. J. Epidemiol..

[604] Altschul R., Hoffer A., Stephen J.D. (1955). Influence of nicotinic acid on serum cholesterol in man. Arch. Biochem. Biophys..

[605] Carlson L.A. (2005). Nicotinic acid: The broad-spectrum lipid drug. A 50th anniversary review. J. Intern. Med..

[606] Wise A., Foord S.M., Fraser N.J., Barnes A.A., Elshourbagy N., Eilert M., Ignar D.M., Murdock P.R., Steplewski K., Green A. (2003). Molecular identification of high and low affinity receptors for nicotinic acid. J. Biol. Chem..

[607] Soga T., Kamohara M., Takasaki J., Matsumoto S., Saito T., Ohishi T., Hiyama H., Matsuo A., Matsushime H., Furuichi K. (2003). Molecular identification of nicotinic acid receptor. Biochem. Biophys. Res. Commun..

[608] Taggart A.K., Kero J., Gan X., Cai T.Q., Cheng K., Ippolito M., Ren N., Kaplan R., Wu K., Wu T.J. (2005). (D)-beta-Hydroxybutyrate inhibits adipocyte lipolysis via the nicotinic acid receptor PUMA-G. J. Biol. Chem..

[609] Gille A., Bodor E.T., Ahmed K., Offermanns S. (2008). Nicotinic acid: Pharmacological effects and mechanisms of action. Annu. Rev. Pharmacol. Toxicol..

[610] Carlson L.A., Oro L., Ostman J. (1968). Effect of a single dose of nicotinic acid on plasma lipids in patients with hyperlipoproteinemia. Acta Med. Scand..

[611] Ganji S.H., Tavintharan S., Zhu D., Xing Y., Kamanna V.S., Kashyap M.L. (2004). Niacin noncompetitively inhibits DGAT2 but not DGAT1 activity in HepG2 cells. J. Lipid Res..

[612] Jin F.Y., Kamanna V.S., Kashyap M.L. (1999). Niacin accelerates intracellular ApoB degradation by inhibiting triacylglycerol synthesis in human hepatoblastoma (HepG2) cells. Arter. Thromb. Vasc. Biol..

[613] Svedmyr N., Harthon L., Lundholm L. (1969). The relationship between the plasma concentration of free nicotinic acid and some of its pharmacologic effects in man. Clin. Pharmacol. Ther..

[614] Barter P.J., Brewer H.B., Chapman M.J., Hennekens C.H., Rader D.J., Tall A.R. (2003). Cholesteryl ester transfer protein: A novel target for raising HDL and inhibiting atherosclerosis. Arter. Thromb. Vasc. Biol..

[615] Hernandez M., Wright S.D., Cai T.Q. (2007). Critical role of cholesterol ester transfer protein in nicotinic acid-mediated HDL elevation in mice. Biochem. Biophys. Res. Commun..

[616] Le Goff W., Guerin M., Chapman M.J. (2004). Pharmacological modulation of cholesteryl ester transfer protein, a new therapeutic target in atherogenic dyslipidemia. Pharmacol. Ther..

[617] Mousa S.S., Block R.C., Mousa S.A. (2010). High Density Lipoprotein (HDL) Modulation Targets. Drugs Future.

[618] Olsson A.G., Schettler G., Habenicht A.J.R. (1994). Nicotinic Acid and Derivatives. Principles and Treatment of Lipoprotein Disorders.

[619] Jin F.Y., Kamanna V.S., Kashyap M.L. (1997). Niacin decreases removal of high-density lipoprotein apolipoprotein A-I but not cholesterol ester by Hep G2 cells. Implication for reverse cholesterol transport. Arter. Thromb. Vasc. Biol..

[620] Meyers C.D., Kashyap M.L. (2004). Pharmacologic elevation of high-density lipoproteins: Recent insights on mechanism of action and atherosclerosis protection. Curr. Opin. Cardiol..

[621] Wu Z.H., Zhao S.P. (2009). Niacin promotes cholesterol efflux through stimulation of the PPARgamma-LXRalpha-ABCA1 pathway in 3T3-L1 adipocytes. Pharmacology.

[622] Knowles H.J., te Poele R.H., Workman P., Harris A.L. (2006). Niacin induces PPARgamma expression and transcriptional activation in macrophages via HM74 and HM74a-mediated induction of prostaglandin synthesis pathways. Biochem. Pharmacol..

[623] Oram J.F., Lawn R.M., Garvin M.R., Wade D.P. (2000). ABCA1 is the cAMP-inducible apolipoprotein receptor that mediates cholesterol secretion from macrophages. J. Biol. Chem..

[624] Yvan-Charvet L., Wang N., Tall A.R. (2010). Role of HDL, ABCA1, and ABCG1 transporters in cholesterol efflux and immune responses. Arter. Thromb. Vasc. Biol..

[625] Takahashi Y., Miyata M., Zheng P., Imazato T., Horwitz A., Smith J.D. (2000). Identification of cAMP analogue inducible genes in RAW264 macrophages. Biochim. Biophys. Acta.

[626] Zhao S.P., Yang J., Li J., Dong S.Z., Wu Z.H. (2008). Effect of niacin on LXRalpha and PPARgamma expression and HDL-induced cholesterol efflux in adipocytes of hypercholesterolemic rabbits. Int. J. Cardiol..

[627] Johnson S., Imai S.I. (2018). NAD(+) biosynthesis, aging, and disease. F1000Research.

[628] Imai S. (2009). From heterochromatin islands to the NAD World: A hierarchical view of aging through the functions of mammalian Sirt1 and systemic NAD biosynthesis. Biochim. Biophys. Acta.

[629] Imai S. (2011). Dissecting systemic control of metabolism and aging in the NAD World: The importance of SIRT1 and NAMPT-mediated NAD biosynthesis. FEBS Lett..

[630] Rehan L., Laszki-Szczachor K., Sobieszczanska M., Polak-Jonkisz D. (2014). SIRT1 and NAD as regulators of ageing. Life Sci..

[631] Poljsak B. (2018). NAMPT-Mediated NAD Biosynthesis as the Internal Timing Mechanism: In NAD+ World, Time Is Running in Its Own Way. Rejuvenation Res..

[632] Massudi H., Grant R., Braidy N., Guest J., Farnsworth B., Guillemin G.J. (2012). Age-associated changes in oxidative stress and NAD+ metabolism in human tissue. PLoS ONE.

[633] Schultz M.B., Sinclair D.A. (2016). Why NAD(+) Declines during Aging: It’s Destroyed. Cell Metab..

[634] Camacho-Pereira J., Tarrago M.G., Chini C.C.S., Nin V., Escande C., Warner G.M., Puranik A.S., Schoon R.A., Reid J.M., Galina A. (2016). CD38 Dictates Age-Related NAD Decline and Mitochondrial Dysfunction through an SIRT3-Dependent Mechanism. Cell Metab..

[635] Mao K., Zhang G. (2021). The role of PARP1 in neurodegenerative diseases and aging. FEBS J..

[636] Covarrubias A.J., Perrone R., Grozio A., Verdin E. (2021). NAD(+) metabolism and its roles in cellular processes during ageing. Nat. Rev. Mol. Cell Biol..

[637] Bai P., Canto C., Oudart H., Brunyanszki A., Cen Y., Thomas C., Yamamoto H., Huber A., Kiss B., Houtkooper R.H. (2011). PARP-1 inhibition increases mitochondrial metabolism through SIRT1 activation. Cell Metab..

[638] Belenky P., Racette F.G., Bogan K.L., McClure J.M., Smith J.S., Brenner C. (2007). Nicotinamide riboside promotes Sir2 silencing and extends lifespan via Nrk and Urh1/Pnp1/Meu1 pathways to NAD+. Cell.

[639] Canto C., Houtkooper R.H., Pirinen E., Youn D.Y., Oosterveer M.H., Cen Y., Fernandez-Marcos P.J., Yamamoto H., Andreux P.A., Cettour-Rose P. (2012). The NAD(+) precursor nicotinamide riboside enhances oxidative metabolism and protects against high-fat diet-induced obesity. Cell Metab..

[640] Wang X., Li H., Ding S. (2014). The effects of NAD+ on apoptotic neuronal death and mitochondrial biogenesis and function after glutamate excitotoxicity. Int. J. Mol. Sci..

[641] Yaku K., Okabe K., Hikosaka K., Nakagawa T. (2018). NAD Metabolism in Cancer Therapeutics. Front. Oncol..

[642] Kennedy B.E., Sharif T., Martell E., Dai C., Kim Y., Lee P.W., Gujar S.A. (2016). NAD(+) salvage pathway in cancer metabolism and therapy. Pharmacol. Res..

[643] Wakade C., Chong R., Bradley E., Thomas B., Morgan J. (2014). Upregulation of GPR109A in Parkinson’s disease. PLoS ONE.

[644] Wakade C., Chong R., Bradley E., Morgan J.C. (2015). Low-dose niacin supplementation modulates GPR109A, niacin index and ameliorates Parkinson’s disease symptoms without side effects. Clin. Case Rep..

[645] Jia H., Li X., Gao H., Feng Z., Li X., Zhao L., Jia X., Zhang H., Liu J. (2008). High doses of nicotinamide prevent oxidative mitochondrial dysfunction in a cellular model and improve motor deficit in a Drosophila model of Parkinson’s disease. J. Neurosci. Res..

[646] Nimmagadda V.K., Makar T.K., Chandrasekaran K., Sagi A.R., Ray J., Russell J.W., Bever C.T. (2017). SIRT1 and NAD+ precursors: Therapeutic targets in multiple sclerosis a review. J. Neuroimmunol..

[647] Kim S.Y., Cohen B.M., Chen X., Lukas S.E., Shinn A.K., Yuksel A.C., Li T., Du F., Ongur D. (2017). Redox Dysregulation in Schizophrenia Revealed by in vivo NAD+/NADH Measurement. Schizophr. Bull..

[648] Benavente C.A., Schnell S.A., Jacobson E.L. (2012). Effects of niacin restriction on sirtuin and PARP responses to photodamage in human skin. PLoS ONE.

[649] Gensler H.L., Williams T., Huang A.C., Jacobson E.L. (1999). Oral niacin prevents photocarcinogenesis and photoimmunosuppression in mice. Nutr. Cancer.

[650] Gehring W. (2004). Nicotinic acid/niacinamide and the skin. J. Cosmet. Dermatol..

[651] Levine D., Even-Chen Z., Lipets I., Pritulo O.A., Svyatenko T.V., Andrashko Y., Lebwohl M., Gottlieb A. (2010). Pilot, multicenter, double-blind, randomized placebo-controlled bilateral comparative study of a combination of calcipotriene and nicotinamide for the treatment of psoriasis. J. Am. Acad. Dermatol..

[652] Park S.M., Li T., Wu S., Li W.Q., Weinstock M., Qureshi A.A., Cho E. (2017). Niacin intake and risk of skin cancer in US women and men. Int. J. Cancer.

[653] Oberwittler H., Baccara-Dinet M. (2006). Clinical evidence for use of acetyl salicylic acid in control of flushing related to nicotinic acid treatment. Int. J. Clin. Pract..

[654] Benyo Z., Gille A., Bennett C.L., Clausen B.E., Offermanns S. (2006). Nicotinic acid-induced flushing is mediated by activation of epidermal langerhans cells. Mol. Pharmacol..

[655] Hanson J., Gille A., Zwykiel S., Lukasova M., Clausen B.E., Ahmed K., Tunaru S., Wirth A., Offermanns S. (2010). Nicotinic acid- and monomethyl fumarate-induced flushing involves GPR109A expressed by keratinocytes and COX-2-dependent prostanoid formation in mice. J. Clin. Investig..

[656] Hay D.L., Poyner D.R. (2009). Calcitonin gene-related peptide, adrenomedullin and flushing. Maturitas.

[657] Wierzbicki A.S. (2011). Niacin: The only vitamin that reduces cardiovascular events. Int. J. Clin. Pr..

[658] McKenney J.M., Proctor J.D., Harris S., Chinchili V.M. (1994). A comparison of the efficacy and toxic effects of sustained-vs immediate-release niacin in hypercholesterolemic patients. JAMA.

[659] Dalton T.A., Berry R.S. (1992). Hepatotoxicity associated with sustained-release niacin. Am. J. Med..

[660] Lawrence S.P. (1993). Transient focal hepatic defects related to sustained-release niacin. J. Clin. Gastroenterol..

[661] Cheng K., Wu T.J., Wu K.K., Sturino C., Metters K., Gottesdiener K., Wright S.D., Wang Z., O’Neill G., Lai E. (2006). Antagonism of the prostaglandin D2 receptor 1 suppresses nicotinic acid-induced vasodilation in mice and humans. Proc. Natl. Acad. Sci. USA.

[662] Parsons W.B. (1960). Activation of peptic ulcer by nicotinic acid. Report of five cases. JAMA.

[663] McCulloch D.K., Kahn S.E., Schwartz M.W., Koerker D.J., Palmer J.P. (1991). Effect of nicotinic acid-induced insulin resistance on pancreatic B cell function in normal and streptozocin-treated baboons. J. Clin. Investig..

[664] Garg A., Grundy S.M. (1990). Nicotinic acid as therapy for dyslipidemia in non-insulin-dependent diabetes mellitus. JAMA.

[665] Canner P.L., Furberg C.D., Terrin M.L., McGovern M.E. (2005). Benefits of niacin by glycemic status in patients with healed myocardial infarction (from the Coronary Drug Project). Am. J. Cardiol..

[666] Elam M.B., Hunninghake D.B., Davis K.B., Garg R., Johnson C., Egan D., Kostis J.B., Sheps D.S., Brinton E.A. (2000). Effect of niacin on lipid and lipoprotein levels and glycemic control in patients with diabetes and peripheral arterial disease: The ADMIT study: A randomized trial. Arterial Disease Multiple Intervention Trial. JAMA.

[667] Grundy S.M., Vega G.L., McGovern M.E., Tulloch B.R., Kendall D.M., Fitz-Patrick D., Ganda O.P., Rosenson R.S., Buse J.B., Robertson D.D. (2002). Efficacy, safety, and tolerability of once-daily niacin for the treatment of dyslipidemia associated with type 2 diabetes: Results of the assessment of diabetes control and evaluation of the efficacy of niaspan trial. Arch. Intern. Med..

[668] Hwang E.S., Song S.B. (2020). Possible Adverse Effects of High-Dose Nicotinamide: Mechanisms and Safety Assessment. Biomolecules.

[669] Williams R.J., Bradway E.M. (1931). The further fractination of yeast nutrilites and their relationship to vitamin B and Wildiers’ "bios". J. Am. Chem. Soc..

[670] Williams R.J., Lyman C.M., Goodyear G.H., Truesdail J.H., Holaday D. (1933). “Pantothenic Acid,” a Growth Determinant of Universal Biological Occurrence. J. Am. Chem. Soc..

[671] Müller M.A., Medlock J., Prágai Z., Warnke I., Litta G., Kleefeldt A., Kaiser K., De Potzolli B. (2019). Vitamins, 9. Vitamin B5. Ullmann’s Encyclopedia of Industrial Chemistry.

[672] Gonzalez-Lopez J., Aliaga L., Gonzalez-Martinez A., Martinez-Toledo M.V. (2016). Pantothenic Acid. Industrial Biotechnology of Vitamins, Biopigments, and Antioxidants.

[673] Schnellbaecher A., Binder D., Bellmaine S., Zimmer A. (2019). Vitamins in cell culture media: Stability and stabilization strategies. Biotechnol. Bioeng..

[674] Webb M.E., Smith A.G., Abell C. (2004). Biosynthesis of pantothenate. Nat. Prod. Rep..

[675] Leonardi R., Jackowski S. (2007). Biosynthesis of Pantothenic Acid and Coenzyme A. EcoSal Plus.

[676] Martinez D.L., Tsuchiya Y., Gout I. (2014). Coenzyme A biosynthetic machinery in mammalian cells. Biochem. Soc. Trans..

[677] Leonardi R., Zhang Y.M., Rock C.O., Jackowski S. (2005). Coenzyme A: Back in action. Prog. Lipid Res..

[678] Ottenhof H.H., Ashurst J.L., Whitney H.M., Saldanha S.A., Schmitzberger F., Gweon H.S., Blundell T.L., Abell C., Smith A.G. (2004). Organisation of the pantothenate (vitamin B5) biosynthesis pathway in higher plants. Plant J..

[679] Chakauya E., Coxon K.M., Whitney H.M., Ashurst J.L., Abell C., Smith A.G. (2006). Pantothenate biosynthesis in higher plants: Advances and challenges. Physiol. Plant.

[680] Webb M.E., Smith A.G., Rébeillé F., Douce R. (2011). Pantothenate Biosynthesis in Higher Plants. Advances in Botanical Research.

[681] Webb M.E., Marquet A., Mendel R.R., Rebeille F., Smith A.G. (2007). Elucidating biosynthetic pathways for vitamins and cofactors. Nat. Prod. Rep..

[682] White W.H., Gunyuzlu P.L., Toyn J.H. (2001). Saccharomyces cerevisiae is capable of de Novo pantothenic acid biosynthesis involving a novel pathway of beta-alanine production from spermine. J. Biol. Chem..

[683] Spry C., Kirk K., Saliba K.J. (2008). Coenzyme A biosynthesis: An antimicrobial drug target. FEMS Microbiol. Rev..

[684] Smith C.M., Song W.O. (1996). Comparative nutrition of pantothenic acid. J. Nutr. Biochem..

[685] Roje S. (2007). Vitamin B biosynthesis in plants. Phytochemistry.

[686] Coxon K.M., Chakauya E., Ottenhof H.H., Whitney H.M., Blundell T.L., Abell C., Smith A.G. (2005). Pantothenate biosynthesis in higher plants. Biochem. Soc. Trans..

[687] Miller J.W., Rucker R.B. (2012). Pantothenic Acid. Present Knowledge in Nutrition.

[688] Walsh J.H., Wyse B.W., Hansen R.G. (1981). Pantothenic acid content of 75 processed and cooked foods. J. Am. Diet. Assoc..

[689] Scientific Committee on Food (2006). Tolerable Upper Intake Levels for Vitamins and Minerals.

[690] Kelly G.S. (2011). Pantothenic acid. Monograph. Altern. Med. Rev..

[691] Willerton E., Cromwell H. (2002). Microbiologic Assay of Natural Pantothenic Acid in Yeast and Liver. Influence of Clarase Digestion. Ind. Eng. Chem. Anal. Ed..

[692] Ball G.F.M. (1998). Pantothenic acid. Bioavailability and Analysis of Vitamins in Foods.

[693] Hall A.P., Moore J.G., Morgan A.F. (1956). B Vitamin content of avocados: Studies reveal California-grown avocados are in superior group of foods as source of pantothenic acid and vitamin B. Calif. Agric..

[694] EFSA Panel on Dietetic Products, Nutrition and Allergies (NDA) (2014). Scientific Opinion on Dietary Reference Values for pantothenic acid. EFSA J..

[695] Ciulu M., Floris I., Nurchi V.M., Panzanelli A., Pilo M.I., Spano N., Sanna G. (2013). HPLC determination of pantothenic acid in royal jelly. Anal. Methods.

[696] Kunugi H., Mohammed Ali A. (2019). Royal Jelly and Its Components Promote Healthy Aging and Longevity: From Animal Models to Humans. Int. J. Mol. Sci..

[697] Pearson P.B., Burgin C.J. (1941). The Pantothenic Acid Content of Royal Jelly. Exp. Biol. Med..

[698] Uebanso T., Shimohata T., Mawatari K., Takahashi A. (2020). Functional Roles of B-Vitamins in the Gut and Gut Microbiome. Mol. Nutr. Food Res..

[699] Bates C.J., Caballero B. (2013). Pantothenic Acid. Encyclopedia of Human Nutrition.

[700] Said H.M., Mohammed Z.M. (2006). Intestinal absorption of water-soluble vitamins: An update. Curr. Opin. Gastroenterol..

[701] Said H.M., Ortiz A., McCloud E., Dyer D., Moyer M.P., Rubin S. (1998). Biotin uptake by human colonic epithelial NCM460 cells: A carrier-mediated process shared with pantothenic acid. Am. J. Physiol..

[702] Ghosal A., Lambrecht N., Subramanya S.B., Kapadia R., Said H.M. (2013). Conditional knockout of the Slc5a6 gene in mouse intestine impairs biotin absorption. Am. J. Physiol. Gastrointest. Liver Physiol..

[703] Said H.M. (2011). Intestinal absorption of water-soluble vitamins in health and disease. Biochem J..

[704] MacDonald R., Reitmeier C. (2017). Food Processing. Understanding Food Systems.

[705] Schroeder H.A. (1971). Losses of vitamins and trace minerals resulting from processing and preservation of foods. Am. J. Clin. Nutr..

[706] Bodwell C., Anderson B. (1986). Nutritional Composition and Value of Meat and Meat Products.

[707] Engler P.P., Bowers J.A. (1976). B-vitamin retention in meat during storage and preparation. A review. J. Am. Diet. Assoc..

[708] Meyer B.H., Mysinger M.A., Wodarski L.A. (1969). Pantothenic acid and vitamin B6 in beef. J. Am. Diet Assoc..

[709] Cheng T.S., Eitenmiller R.R. (1988). Effects of Processing and Storage on the Pantothenic-Acid Content of Spinach and Broccoli. J. Food Process. Preserv..

[710] Hoppner K., Lampi B. (1993). Pantothenic-Acid and Biotin Retention in Cooked Legumes. J. Food Sci..

[711] Khalil A.H., Mansour E.H. (1995). The Effect of Cooking, Autoclaving and Germination on the Nutritional Quality of Faba Beans. Food Chem..

[712] Kilgore S.M., Sistrunk W.A. (1981). Effects of Soaking Treatments and Cooking Upon Selected B-Vitamins and the Quality of Blackeyed Peas. J. Food Sci..

[713] Rolls B.A., Porter J.W.G. (1973). Some effects of processing and storage on the nutritive value of milk and milk products. Proc. Nutr Soc..

[714] King J.C., Blumberg J., Ingwersen L., Jenab M., Tucker K.L. (2008). Tree nuts and peanuts as components of a healthy diet. J. Nutr..

[715] Arya S.S., Salve A.R., Chauhan S. (2016). Peanuts as functional food: A review. J. Food Sci. Technol..

[716] Sathe S.K., Monaghan E.K., Kshirsagar H.H., Venkatachalam M. (2009). Chemical composition of edible nut seeds and its implications in human health. Tree Nuts: Composition, Phytochemicals, and Health Effects.

[717] Dreher M.L., Davenport A.J. (2013). Hass avocado composition and potential health effects. Crit. Rev. Food Sci. Nutr..

[718] Chen C.Y., Lapsley K., Blumberg J. (2006). A nutrition and health perspective on almonds. J. Sci. Food Agric..

[719] Yada S., Lapsley K., Huang G.W. (2011). A review of composition studies of cultivated almonds: Macronutrients and micronutrients. J. Food Compos. Anal..

[720] Barreca D., Nabavi S.M., Sureda A., Rasekhian M., Raciti R., Silva A.S., Annunziata G., Arnone A., Tenore G.C., Suntar I. (2020). Almonds (*Prunus Dulcis* Mill. D. A. Webb): A Source of Nutrients and Health-Promoting Compounds. Nutrients.

[721] Roncero J.M., Alvarez-Orti M., Pardo-Gimenez A., Rabadan A., Pardo J.E. (2020). Review about Non-Lipid Components and Minor Fat-Soluble Bioactive Compounds of Almond Kernel. Foods.

[722] Ahmad R.S., Imran A., Hussain M.B. (2018). Nutritional Composition of Meat.

[723] Li C. (2017). The role of beef in human nutrition and health. Ensuring Safety and Quality in the Production of Beef.

[724] Probst Y. (2009). Nutrient Compostion of Chicken Meat.

[725] Van Heerden S.M., Schönfeldt H.C., Smith M.F., Jansen van Rensburg D.M. (2002). Nutrient Content of South African Chickens. J. Food Compos. Anal..

[726] Dunn K.R., Goddard V.R. (1948). Effect of heat upon the nutritive values of peanuts; riboflavin and pantothenic acid content. Food Res..

[727] Muhamad N., Yusoff M.M., Gimbun J. (2015). Thermal degradation kinetics of nicotinic acid, pantothenic acid and catechin derived from Averrhoa bilimbi fruits. RSC Adv..

[728] Ford J.E., Hurrell R.F., Finot P.A. (1983). Storage of milk powders under adverse conditions. 2. Influence on the content of water-soluble vitamins. Br. J. Nutr..

[729] Gutzeit D., Klaubert B., Rychlik M., Winterhalter P., Jerz G. (2007). Effects of processing and of storage on the stability of pantothenic acid in sea buckthorn products (*Hippophae rhamnoides* L. ssp. rhamnoides) assessed by stable isotope dilution assay. J. Agric. Food Chem..

[730] Pearson A., West R., Luecke R. (1959). The vitamin and amino acid content of drip obtained upon defrosting frozen pork. Food Res..

[731] Pearson A.M., Burnside J.E., Edwards H.M., Glasscock R.S., Cunha T.J., Novak A.F. (1951). Vitamin losses in drip obtained upon defrosting frozen meat. Food Res..

[732] Ledesma-Amaro R., Santos M.A., Jiménez A., Revuelta J.L., McNeil B., Archer D., Giavasis I., Harvey L. (2013). Microbial production of vitamins. Microbial Production of Food Ingredients, Enzymes and Nutraceuticals.

[733] Shimizu S. (2001). Vitamins and Related Compounds: Microbial Production. Biotechnology.

[734] Shimizu S., Kataoka M., Honda K., Sakamoto K. (2001). Lactone-ring-cleaving enzymes of microorganisms: Their diversity and applications. J. Biotechnol..

[735] Li H., Lu X., Chen K., Yang J., Zhang A., Wang X., Ouyang P. (2018). β-alanine production using whole-cell biocatalysts in recombinant *Escherichia coli*. Mol. Catal..

[736] Shen Y., Zhao L., Li Y., Zhang L., Shi G. (2014). Synthesis of beta-alanine from L-aspartate using L-aspartate-alpha-decarboxylase from Corynebacterium glutamicum. Biotechnol. Lett..

[737] Wang L., Mao Y., Wang Z., Ma H., Chen T. (2021). Advances in biotechnological production of beta-alanine. World J. Microbiol. Biotechnol..

[738] Laudert D., Hohmann H.P., Moo-Young M. (2011). Application of Enzymes and Microbes for the Industrial Production of Vitamins and Vitamin-like Compounds. Comprehensive Biotechnology.

[739] Bonrath W., Netscher T., Eggersdorfer M., Adam G. (2019). Vitamins, 1. troduction. In Ullmann’s Encyclopedia of Industrial Chemistry.

[740] Zu Berstenhorst S.M., Hohmann H.P., Stahmann K.P., Schaechter M. (2009). Vitamins and Vitamin-like Compounds: Microbial Production. Encyclopedia of Microbiology.

[741] Wang Y., Liu L., Jin Z., Zhang D. (2021). Microbial Cell Factories for Green Production of Vitamins. Front. Bioeng. Biotechnol..

[742] Dusch N., Puhler A., Kalinowski J. (1999). Expression of the Corynebacterium glutamicum panD gene encoding L-aspartate-alpha-decarboxylase leads to pantothenate overproduction in Escherichia coli. Appl. Environ. Microbiol..

[743] Huser A.T., Chassagnole C., Lindley N.D., Merkamm M., Guyonvarch A., Elisakova V., Patek M., Kalinowski J., Brune I., Puhler A. (2005). Rational design of a Corynebacterium glutamicum pantothenate production strain and its characterization by metabolic flux analysis and genome-wide transcriptional profiling. Appl. Environ. Microbiol..

[744] Tigu F., Zhang J., Liu G., Cai Z., Li Y. (2018). A highly active pantothenate synthetase from Corynebacterium glutamicum enables the production of D-pantothenic acid with high productivity. Appl. Microbiol. Biotechnol..

[745] Chassagnole C., Diano A., Letisse F., Lindley N.D. (2003). Metabolic network analysis during fed-batch cultivation of *Corynebacterium glutamicum* for pantothenic acid production: First quantitative data and analysis of by-product formation. J. Biotechnol..

[746] Zhang B., Zhang X.M., Wang W., Liu Z.Q., Zheng Y.G. (2019). Metabolic engineering of *Escherichia coli* for d-pantothenic acid production. Food Chem..

[747] Zou S.P., Wang Z.J., Zhao K., Zhang B., Niu K., Liu Z.Q., Zheng Y.G. (2020). High-level production of d-pantothenic acid from glucose by fed-batch cultivation of *Escherichia coli*. Biotechnol. Appl. Biochem..

[748] Zou S.P., Zhao K., Wang Z.J., Zhang B., Liu Z.Q., Zheng Y.G. (2021). Overproduction of D-pantothenic acid via fermentation conditions optimization and isoleucine feeding from recombinant *Escherichia coli* W3110. 3 Biotech.

[749] Woollard D.C., Indyk H.E., Christiansen S.K. (2000). The analysis of pantothenic acid in milk and infant formulas by HPLC. Food Chem..

[750] Romera J.M., Ramirez M., Gil A. (1996). Determination of pantothenic acid in infant milk formulas by high performance liquid chromatography. J. Dairy Sci..

[751] Mittermayr R., Kalman A., Trisconi M.J., Heudi O. (2004). Determination of vitamin B5 in a range of fortified food products by reversed-phase liquid chromatography-mass spectrometry with electrospray ionisation. J. Chromatogr. A.

[752] Andrieux P., Fontannaz P., Kilinc T., Gimenez E.C. (2012). Pantothenic acid (vitamin B5) in fortified foods: Comparison of a novel ultra-performance liquid chromatography-tandem mass spectrometry method and a microbiological assay (AOAC Official Method 992.07). J. AOAC Int..

[753] Lu B., Ren Y., Huang B., Liao W., Cai Z., Tie X. (2008). Simultaneous determination of four water-soluble vitamins in fortified infant foods by ultra-performance liquid chromatography coupled with triple quadrupole mass spectrometry. J. Chromatogr. Sci..

[754] Chakauya E., Coxon K.M., Wei M., Macdonald M.V., Barsby T., Abell C., Smith A.G. (2008). Towards engineering increased pantothenate (vitamin B(5)) levels in plants. Plant Mol. Biol..

[755] Rucker R.B. (2016). Pantothenic Acid.

[756] Czumaj A., Szrok-Jurga S., Hebanowska A., Turyn J., Swierczynski J., Sledzinski T., Stelmanska E. (2020). The Pathophysiological Role of CoA. Int. J. Mol. Sci..

[757] Mindrebo J.T., Patel A., Misson L.E., Kim W.E., Davis T.D., Ni Q.Z., La Clair J.J., Burkart M.D., Hung-Wen L., Begley T.P. (2020). 1.04-Structural Basis of Acyl-Carrier Protein Interactions in Fatty Acid and Polyketide Biosynthesis. Comprehensive Natural Products III.

[758] Naquet P., Kerr E.W., Vickers S.D., Leonardi R. (2020). Regulation of coenzyme A levels by degradation: The “Ins and Outs”. Prog. Lipid Res..

[759] Goding J.W., Grobben B., Slegers H. (2003). Physiological and pathophysiological functions of the ecto-nucleotide pyrophosphatase/phosphodiesterase family. Biochim. Biophys. Acta.

[760] Shibata K., Gross C.J., Henderson L.M. (1983). Hydrolysis and absorption of pantothenate and its coenzymes in the rat small intestine. J. Nutr..

[761] Bartucci R., Salvati A., Olinga P., Boersma Y.L. (2019). Vanin 1: Its Physiological Function and Role in Diseases. Int. J. Mol. Sci..

[762] Turner J.B., Hughes D.E. (1962). The absorption of some B-group vitamins by surviving rat intestine preparations. Q. J. Exp. Physiol. Cogn. Med. Sci..

[763] Prasad P.D., Wang H., Huang W., Fei Y.J., Leibach F.H., Devoe L.D., Ganapathy V. (1999). Molecular and functional characterization of the intestinal Na+-dependent multivitamin transporter. Arch. Biochem. Biophys..

[764] Ono S., Kameda K., Abiko Y. (1974). Metabolism of panthethine in the rat. J. Nutr. Sci. Vitam..

[765] Wittwer C.T., Gahl W.A., Butler J.D., Zatz M., Thoene J.G. (1985). Metabolism of pantethine in cystinosis. J. Clin. Investig..

[766] Karnitz L.M., Gross C.J., Henderson L.M. (1984). Transport and metabolism of pantothenic acid by rat kidney. Biochim. Biophys. Acta.

[767] Eissenstat B.R., Wyse B.W., Hansen R.G. (1986). Pantothenic acid status of adolescents. Am. J. Clin. Nutr..

[768] Annous K.F., Song W.O. (1995). Pantothenic acid uptake and metabolism by red blood cells of rats. J. Nutr..

[769] Vadlapudi A.D., Vadlapatla R.K., Mitra A.K. (2012). Sodium dependent multivitamin transporter (SMVT): A potential target for drug delivery. Curr. Drug Targets.

[770] Grassl S.M. (1992). Human placental brush-border membrane Na(+)-pantothenate cotransport. J. Biol. Chem..

[771] Uchida Y., Ito K., Ohtsuki S., Kubo Y., Suzuki T., Terasaki T. (2015). Major involvement of Na(+) -dependent multivitamin transporter (SLC5A6/SMVT) in uptake of biotin and pantothenic acid by human brain capillary endothelial cells. J. Neurochem..

[772] Boger W.P., Bayne G.M., Gylfe J., Wright L.D. (1953). Renal clearance of pantothenic acid in man; inhibition by probenecid (benemid). Proc. Soc. Exp. Biol. Med..

[773] Spector H., Hamilton T.S., Mitchell H.H. (1945). The effect of pantothenic acid dosage and environmental temperature and humidity upon the dermal and renal excretion of pantothenic acid. J. Biol. Chem..

[774] Tsuji T., Fukuwatari T., Sasaki S., Shibata K. (2010). Urinary excretion of vitamin B1, B2, B6, niacin, pantothenic acid, folate, and vitamin C correlates with dietary intakes of free-living elderly, female Japanese. Nutr. Res..

[775] Tsuji T., Fukuwatari T., Sasaki S., Shibata K. (2010). Twenty-four-hour urinary water-soluble vitamin levels correlate with their intakes in free-living Japanese university students. Eur. J. Clin. Nutr..

[776] Hodges R.E., Ohlson M.A., Bean W.B. (1958). Pantothenic acid deficiency in man. J. Clin. Investig..

[777] Hodges R.E., Bean W.B., Ohlson M.A., Bleiler R. (1959). Human pantothenic acid deficiency produced by omega-methyl pantothenic acid. J. Clin. Investig..

[778] Drell W., Dunn M.S. (1951). Production of pantothenic acid deficiency syndrome in mice with-methylpantothenic acid. Arch. Biochem. Biophys..

[779] Pudelkewicz C., Roderuck C. (1960). Pantothenic acid deficiency in the young guinea pig. J. Nutr..

[780] Bean W.B., Hodges R.E., Daum K. (1955). Pantothenic acid deficiency induced in human subjects. J. Clin. Investig..

[781] Nelson M.M., Evans H.M. (1946). Pantothenic acid deficiency and reproduction in the rat. J. Nutr..

[782] Chen M.-C., Song Y., Song W.O. (1996). Fetal growth retardation and death in pantothenic acid-deficient rats is due to imparired placental function. J. Nutr. Biochem..

[783] Olson R.E., Kaplan N.O. (1948). The effect of pantothenic acid deficiency upon the coenzyme A content and pyruvate utilization of rat and duck tissues. J. Biol. Chem..

[784] Schaefer A.E., McKibbin J.M., Elvehjem C.A. (1942). Pantothenic acid deficiency studies in the dog. J. Biol. Chem..

[785] Guehring R.R., Hurley L.S., Morgan A.F. (1952). Cholesterol metabolism in pantothenic acid deficiency. J. Biol. Chem..

[786] Fry P.C., Fox H.M., Tao H.G. (1976). Metabolic response to a pantothenic acid deficient diet in humans. J. Nutr. Sci. Vitam..

[787] Xu J., Patassini S., Begley P., Church S., Waldvogel H.J., Faull R.L.M., Unwin R.D., Cooper G.J.S. (2020). Cerebral deficiency of vitamin B5 (d-pantothenic acid; pantothenate) as a potentially-reversible cause of neurodegeneration and dementia in sporadic Alzheimer’s disease. Biochem. Biophys. Res. Commun..

[788] Johnson M.A., Kuo Y.M., Westaway S.K., Parker S.M., Ching K.H., Gitschier J., Hayflick S.J. (2004). Mitochondrial localization of human PANK2 and hypotheses of secondary iron accumulation in pantothenate kinase-associated neurodegeneration. Ann. N. Y. Acad. Sci..

[789] Kurian M.A., McNeill A., Lin J.P., Maher E.R. (2011). Childhood disorders of neurodegeneration with brain iron accumulation (NBIA). Dev. Med. Child Neurol..

[790] Pratini N.R., Sweeters N., Vichinsky E., Neufeld J.A. (2013). Treatment of classic pantothenate kinase-associated neurodegeneration with deferiprone and intrathecal baclofen. Am. J. Phys. Med. Rehabil..

[791] Gregory A., Hayflick S.J., Adam M.P., Ardinger H.H., Pagon R.A., Wallace S.E., Bean L.J.H., Mirzaa G., Amemiya A. (1993). Pantothenate Kinase-Associated Neurodegeneration. GeneReviews^®^.

[792] Hatano M. (1962). Pantothenic acid deficiency in rats. J. Vitam..

[793] Seronde J. (1963). The Pathogenesis of Duodenal Ulcer Disease in the Pantothenate-Deficient Rat. Yale J. Biol. Med..

[794] Jones J.H., Foster C., Dorfman F., Hunter G.L., Quinby M.E., Alexander D.L. (1945). Effects on the Albino Mouse of Feeding Diets Very Deficient in Each of Several Vitamin B Factors. J. Nutr..

[795] Berg B.N. (1959). Duodenitis and duodenal ulcers produced in rats by pantothenic acid deficiency. Br. J. Exp. Pathol..

[796] Osborn M.O., Weaver C., Anderson J. (1958). Cholesterol in blood and tissues of adult pantothenic acid-deficient rats. J. Nutr..

[797] Groody T.C., Groody M.E. (1942). Feather Depigmentation and Pantothenic Acid Deficiency in Chicks. Science.

[798] Gries C.L., Scott M.L. (1972). The pathology of thiamin, riboflavin, pantothenic acid and niacin deficiencies in the chick. J. Nutr..

[799] Jukes T.H. (1939). Pantothenic acid and the filtrate (chick anti-dermatitis) factor. J. Am. Chem. Soc..

[800] Wintrobe M.M., Follis R.H., Alcayaga R., Paulson M., Humphreys S. (1943). Pantothenic acid deficiency in swine with particular reference to the effects on growth and on the alimentary tract. Bul. Johns Hopkins Hosp..

[801] Follis R.H., Wintrobe M.M. (1945). A Comparison of the Effects of Pyridoxine and Pantothenic Acid Deficiencies on the Nervous Tissues of Swine. J. Exp. Med..

[802] Nelson R.A. (1968). Intestinal transport, coenzyme A, and colitis in pantothenic acid deficiency. Am. J. Clin. Nutr..

[803] Silber R.H. (1944). Studies of Pantothenic Acid Deficiency in Dogs: Three Figures. J. Nutr..

[804] Wittwer C.T., Graves C.P., Peterson M.A., Jorgensen E., Wilson D.E., Thoene J.G., Wyse B.W., Windham C.T., Hansen R.G. (1987). Pantethine lipomodulation: Evidence for cysteamine mediation in vitro and in vivo. Atherosclerosis.

[805] Evans M., Rumberger J.A., Azumano I., Napolitano J.J., Citrolo D., Kamiya T. (2014). Pantethine, a derivative of vitamin B5, favorably alters total, LDL and non-HDL cholesterol in low to moderate cardiovascular risk subjects eligible for statin therapy: A triple-blinded placebo and diet-controlled investigation. Vasc. Health Risk Manag..

[806] Gaddi A., Descovich G.C., Noseda G., Fragiacomo C., Colombo L., Craveri A., Montanari G., Sirtori C.R. (1984). Controlled evaluation of pantethine, a natural hypolipidemic compound, in patients with different forms of hyperlipoproteinemia. Atherosclerosis.

[807] Arsenio L., Caronna S., Lateana M., Magnati G., Strata A., Zammarchi G. (1984). Hyperlipidemia, diabetes and atherosclerosis: Efficacy of treatment with pantethine. Acta Biomed. Ateneo Parm..

[808] Donati C., Bertieri R.S., Barbi G. (1989). Pantethine, diabetes mellitus and atherosclerosis. Clinical study of 1045 patients. Clin. Ter..

[809] Coronel F., Tornero F., Torrente J., Naranjo P., De Oleo P., Macia M., Barrientos A. (1991). Treatment of hyperlipemia in diabetic patients on dialysis with a physiological substance. Am. J. Nephrol..

[810] Orloff S., Butler J.D., Towne D., Mukherjee A.B., Schulman J.D. (1981). Pantetheinase activity and cysteamine content in cystinotic and normal fibroblasts and leukocytes. Pediatr. Res..

[811] Capodice J.L. (2012). Feasibility, Tolerability, Safety and Efficacy of a Pantothenic Acid Based Dietary Supplement in Subjects with Mild to Moderate Facial Acne Blemishes. J. Cosmet. Dermatol. Sci. Appl..

[812] Yang M., Moclair B., Hatcher V., Kaminetsky J., Mekas M., Chapas A., Capodice J. (2014). A randomized, double-blind, placebo-controlled study of a novel pantothenic Acid-based dietary supplement in subjects with mild to moderate facial acne. Dermatol. Ther..

[813] Proksch E., de Bony R., Trapp S., Boudon S. (2017). Topical use of dexpanthenol: A 70th anniversary article. J. Dermatol. Treat..

[814] Wollina U., Kubicki J. (2006). Dexpanthenol supports healing of superficial wounds and injuries. Kosm. Med..

[815] Proksch E., Nissen H.P. (2002). Dexpanthenol enhances skin barrier repair and reduces inflammation after sodium lauryl sulphate-induced irritation. J. Dermatol. Treat..

[816] Stettler H., Kurka P., Lunau N., Manger C., Bohling A., Bielfeldt S., Wilhelm K.P., Dahnhardt-Pfeiffer S., Dahnhardt D., Brill F.H. (2017). A new topical panthenol-containing emollient: Results from two randomized controlled studies assessing its skin moisturization and barrier restoration potential, and the effect on skin microflora. J. Dermatol. Treat..

[817] Heise R., Schmitt L., Huth L., Krings L., Kluwig D., Katsoulari K.V., Steiner T., Holzle F., Baron J.M., Huth S. (2019). Accelerated wound healing with a dexpanthenol-containing ointment after fractional ablative CO_2_ laser resurfacing of photo-damaged skin in a randomized prospective clinical trial. Cutan. Ocul. Toxicol..

[818] Wananukul S., Limpongsanuruk W., Singalavanija S., Wisuthsarewong W. (2006). Comparison of dexpanthenol and zinc oxide ointment with ointment base in the treatment of irritant diaper dermatitis from diarrhea: A multicenter study. J. Med. Assoc. Thai.

[819] Olsavszky R., Nanu E.A., Macura-Biegun A., Kurka P., Trapp S. (2019). Skin barrier restoration upon topical use of two 5% dexpanthenol water-in-oil formulations on freshly tattooed skin: Results from a single-blind prospective study. Wounds Int..

[820] Udompataikul M., Limpa-o-vart D. (2012). Comparative trial of 5% dexpanthenol in water-in-oil formulation with 1% hydrocortisone ointment in the treatment of childhood atopic dermatitis: A pilot study. J. Drugs Dermatol..

[821] Shanazi M., Farshbaf Khalili A., Kamalifard M., Asghari Jafarabadi M., Masoudin K., Esmaeli F. (2015). Comparison of the Effects of Lanolin, Peppermint, and Dexpanthenol Creams on Treatment of Traumatic Nipples in Breastfeeding Mothers. J. Caring Sci..

[822] Hamdi I.M. (2018). Effect of D-Panthenol on Corneal Epithelial Healing after Surface Laser Ablation. J. Ophthalmol..

[823] Gobbels M., Gross D. (1996). Clinical study of the effectiveness of a dexpanthenol containing artificial tears solution (Siccaprotect) in treatment of dry eyes. Klin. Monbl. Augenheilkd..

[824] Jagade M.V., Langade D.G., Pophale R.R., Prabhu A. (2008). Oxymetazoline plus dexpanthenol in nasal congestion. Indian J. Otolaryngol. Head Neck Surg..

[825] Kehrl W., Sonnemann U., Dethlefsen U. (2003). Advance in therapy of acute rhinitis—Comparison of efficacy and safety of xylometazoline in combination xylometazoline-dexpanthenol in patients with acute rhinitis. Laryngo-Rhino-Otologie.

[826] Kehrl W., Sonnemann U. (2000). Improving wound healing after nose surgery by combined administration of xylometazoline and dexpanthenol. Laryngo-Rhino-Otologie.

[827] Unna K., Greslin J.G. (1941). Studies on the toxicity and pharmacology of pantothenic acid. J. Pharmacol. Exp. Ther..

[828] Flodin N.W. (1988). Pharmacology of Micronutrients.

[829] Debourdeau P.M., Djezzar S., Estival J.L., Zammit C.M., Richard R.C., Castot A.C. (2001). Life-threatening eosinophilic pleuropericardial effusion related to vitamins B5 and H. Ann. Pharmacother..

