# Supplementary material for: Biological Properties of Vitamins of the B-Complex, Part 1: Vitamins B1, B2, B3, and B5
Source: Nutrients. 2022 Jan 22;14(3):484. doi: 10.3390/nu14030484 (PMC8839250; doi:10.3390/nu14030484)
Supplement: Supplementary file 1 [file nutrients-14-00484-s001.zip › nutrients-1550678-supplementary.pdf]

Biological properties of vitamins of the B-complex, part 1 – vitamins B_1, B_2, B_3 and B_5

Marcel Hrubša[1], Tomáš Siatka[2], Iveta Nejmanová[3], Marie Vopršalová[1], Lenka Kujovská Krčmová[4,6], Kateřina Matoušová[6], Lenka Javorská[6], Kateřina Macáková[2], Laura Mercolini[7], Fernando Remião[8], Marek Máťuš[5]* and Přemysl Mladěnka[1]

SUPPLEMENTARY DATA

13 PAGES

Table S1: Detailed summary of methods for determination of vitamin B_{1-5} in human biological materials

technique	sensitivity nmol/L	analytes	matrix	advantages	disadvantages	ref.	publication year
LC-MS	LOQ<br>0.15 – 8.12<br>(B_1 – B_3-NAM)	B_1, B_3-NAM, B_2-FAD, B_2, and others	human milk<br>serum	* short analysis time<br>* small sample volume (50 µL)<br>* sensitivity	* SIM<br>* complicated sample preparation (breast milk)	[1]	2012
	LLOQ<br>B_1-TPP 12	B_1-TPP, and others	whole blood	* 250 µL sample volume<br>* MRM<br>* simple sample preparation<br>* short analysis time	* complicated gradient elution	[2]	2017
	LLOQ<br>1.54 – 15.63	B_1, B_1-TPP, B_1-TMP	whole blood	* 200 µL sample volume<br>* simple sample preparation<br>* MRM<br>* sensitivity		[3]	2018
	LOD<br>0.189 – 2.28<br>(B_1 – B_5)	B_1, B_2, B_2-FAD, B_3-NAM, B_5, and others	human milk	* MRM<br>* short analysis time<br>* sensitivity	* 1 mL sample	[4]	2015
	LLOQ<br>21.9	B_1-TPP	dry blood (VAMS)	* MRM<br>* 2 min analysis<br>* simple extraction	* complicated gradient elution	[5]	2021
	LOQ<br>1.58 – 40.94<br>(B_1 – B_3-NAM)	$B_{1,2,3,5}$<br>B_3-NAM, and others	whole blood	* simple extraction	* validated only for 9 analytes<br>* SIM	[6]	2021
	LOD<br>0.41 – 0.91<br>(B_3 – B_5)	$B_{2,3,5,}$	plasma	* small sample volume (100 µL)	* SIM<br>* complicated sample preparation	[7]	2020

				* small solvents volumes<br>* sensitivity	* long analysis time		
	LLOQ<br>1.33	B_2	plasma	* small sample volume (100 µL)<br>* MRM<br>* simple sample preparation<br>* sensitivity		[8]	2019
	LOQ<br>13.29	B_2	plasma	* simple sample preparation	* long analysis time<br>* SIM	[9]	2020
	LLOQ<br>1 – 60<br>(B_1 – B_5)	$B_{1,2,3,5}$ and its vitamers (21 analytes)	plasma	* simple sample preparation<br>* MRM<br>* sensitivity		[10]	2015
	LOQ<br>26.57 – 246.32<br>(B_3 – B_5)	fat + water soluble vitamins (12 analytes)<br>$B_{1,2,3,5}$	serum<br>tears	* one extraction for both groups of vitamins<br>* simple sample preparation<br>* MRM<br>* small sample volume (200 µL serum, 70 µL tears)	* not sufficient validation parameters for all analytes	[11]	2018
	LOD<br>29.23	B_2	urine	* small sample volume (50 µL)<br>* MRM		[12]	2011
	LLOQ<br>45.61	B_5,<br>7 analytes	plasma	* simple sample preparation in 96well plate<br>* small sample volume (50 µL)<br>* short analysis time	* SIM	[13]	2016

	LOQ 4.27 – 5.17 (B_1 – B_1-TMP)	B_1, B_1-TPP, B_1-TMP	whole blood	* 250 µL sample volume	* no IS<br>* complicated sample preparation<br>* derivatization<br>* long analysis time	[14]	2020
	LLOQ 23.51	B_1-TPP	dry blood spot		* complicated sample preparation<br>* derivatization<br>* long analysis time	[15]	2019
HPLC-FLD	LOQ 1.5 – 3	B_1, B_1-TMP, B_1-TPP	dry blood spot	* microplates used in sample preparation<br>* sensitivity	* complicated sample preparation<br>* toxic solvents usage	[16]	2020
	LLOQ 20	B_1-TPP	whole blood	* small sample volume (100 µL)<br>* simple derivatization procedure	* no IS	[17]	2020
	LLOQ 1	B_2, B_2-FAD, B_2-FMN	plasma	* simple sample preparation<br>* microplates usage<br>* sensitivity		[18]	2011
	LOQ 0.5	B_1, B_1-TMP, B_1-TPP	whole blood human milk	* sensitivity	* derivatization<br>* no IS	[19]	2012
HPLC-PDA	LOD B_2 212.56	B_2	urine	* simple sample preparation	* long analysis time	[20]	2009
	LOD B_1 1.48×10^3 B_3-NAM 4.09×10^3 B_2 0.53×10^3	B_1, B_3-NAM, B_2	urine plasma		* no optimal recovery<br>* long analysis time<br>* poor sensitivity	[21]	2014
Sensors / nanodots / CL / FLD / ECD	LOD 1	B_1	urine	* simple<br>* cheap<br>* small sample (100 µL) and solvents volumes	* research only - not commercially available<br>* indirect detection	[22]	2002

				* no sample preparation<br>* short analysis<br>* sensitivity			
	LOD<br>6.8×10^{-6}	B_1	serum	* selectivity<br>* simple sample preparation	* preparation of functionalized gold nanoparticles	[23]	2015
	LOD<br>0.717	B_1	serum<br>urine	* sensitivity	* indirect detection	[24]	2017
	LOD<br>0.25×10^{3}	B_1	serum<br>urine		* indirect determination<br>* not sensitive for biological material<br>* poor sensitivity	[25]	2018
	LOD<br>0.86	B_2	plasma	* sensitivity	* not available in the market<br>* research only<br>* standard addition method not suitable in clinical analysis	[26]	2020
SFC-MS	not specified	B_3 and its metabolites	urine<br>rabbit plasma	* short analysis time<br>* simple sample preparation	* no data using human biofluids available	[27,28]	
Microbiological test kits	LLOQ<br>83.93	B_5	serum	* small sample volume (50 µL)	* high price (working in duplicate recommended)<br>* long analysis time (24 h)	[29]	
	LLOQ<br>64.98	B_3	serum	* small sample volume (100 µL)	* high price (working in duplicate recommended)<br>* long analysis time (24 h)	[30]	

HPLC-FLD (kits)	LOD<br>B_1-TPP 1.18<br>B_2-FAD 12.71	B_1-TPP, B_2-FAD and others	plasma<br>whole blood	* small sample volume (100 – 300 µL)<br>* sensitivity	* no IS<br>* long analysis time<br>* different extraction procedures for each vitamin<br>* different analysis conditions (temperature etc.)<br>* high price for small sample series	[31]	2021
	LOD<br>1.18	B_1-TPP	whole blood	* small sample volume (50 µL)<br>* sensitivity	* long analysis time<br>* high price for small sample series	[32]	2021
	LOD<br>B_1 1.89<br>B_1-TPP 1.18<br>B_1-TMP 1.45	B_1, B_1-TPP, B_1-TMP	whole blood	* short analysis time<br>* possible to use for B_2 and B_6 vitamins determination<br>* sensitivity	* high price for small sample series	[33]	2021
	LOD only for B_2-FAD 12.7	B_2, B_2-FAD, B_2-FMN	whole blood	* small sample volume (200 µL)<br>* possible to use for B_1 and B_6 vitamins determination	* high price for small sample series	[34]	2021
LC-MS/MS (kits)	LOD<br>B_1-TPP 4.4<br>B_2-FAD 3.41	B_1-TPP, B_2-FAD and others	whole blood	* IS<br>* MRM<br>* short analysis time<br>* small sample volume (50 µL)<br>* simple sample preparation<br>* sensitivity	* high price for small sample series	[35]	2021
ELISA kits	LOD<br>6.93	B_2	serum<br>plasma	* small sample volume (250 µL)	* for research only<br>* cross reactivity with analogues	[36]	2021

			cell culture<br>supernatant<br>tissue<br>breast milk<br>sperm<br>…	* one kit for various matrices	* time and money consuming for small sample series		
	LOD<br>6.78	B_2	serum<br>plasma	* small sample volume (50 µL)<br>* one kit for various matrices	* for research only<br>* time and money consuming for small sample series	[37]	2021
	LOD<br>0.93×10^{-3}	B_1	serum<br>plasma<br>urine<br>cell culture<br>supernatant<br>tissue	* small sample volume (40 µL)<br>* one kit for various matrices<br>* sensitivity	* for research only<br>* time and money consuming for small sample series	[38]	2021

[1] LOD Limit of Detection, [2] LOQ Limit of Quantitation, [3] LLOQ Lower Limit of Quantitation

$B_{1,}$ thiamine; B_1-TPP, thiamine pyrophosphate/diphosphate; B_1-TMP, thiamine monophosphate; $B_{2,}$ riboflavin; B_2-FAD, flavin adenine dinucleotide; B_2-FMN, flavin adenine mononucleotide; $B_{3,}$ niacin; B_3-NAM, nicotinamide; B_5, pantothenic acid; CL, Chemiluminescence; ECD, Electrochemical Detection; ELISA, Enzyme-Linked ImmunoSorbent Assay; FLD, Fluorescence Detection; HPLC, High Performance Liquid Chromatography; IS, Internal Standard; LC-MS, Coupling of Liquid Chromatography and Mass Spectrometry; MRM, Multiple Reaction Monitoring; MS, Mass Spectrometry; MS/MS, Tandem Mass Spectrometry; PDA, Photodiode-Array Detection; SFC-MS, Coupling of Supercritical Fluid Chromatography and Mass Spectrometry; SIM, Selected Ion Monitoring; VAMS, Volumetric Absorptive Microsampling.

Table S2: Brief overview of human flavoproteins

Enzyme	Cofactor
D-lactate dehydrogenase	FAD
Xanthine dehydrogenase	FAD
(S)-2-hydroxy-acid oxidase	FMN
Glycerol 3-phosphate dehydrogenase	FAD
Choline dehydrogenase	FAD
L-2-Hydroxyglutarate dehydrogenase	FAD
D-2-Hydroxyglutarate dehydrogenase	FAD
Aldehyde oxidase	FAD
Dihydropyrimidine dehydrogenase	FMN
3β-Hydroxysterol δ^{24}-reductase	FAD
Dihydroorotate dehydrogenase	FMN
Protoporphyrinogen IX oxidase	FAD
Acyl-CoA oxidase	FAD
Glutaryl-CoA oxidase	FAD
Succinate dehydrogenase	FAD
Short-chain-(butyryl-) acyl CoA dehydrogenase	FAD
Medium-chain acyl-CoA dehydrogenase	FAD
Glutaryl-CoA dehydrogenase	FAD
Isovaleryl-CoA dehydrogenase	FAD
2-Methylbutyryl-CoA dehydrogenase	FAD
Long-chain-acyl-CoA dehydrogenase	FAD
Very long-chain acyl-CoA dehydrogenase	FAD
Isobutyryl-CoA dehydrogenase	FAD
Long-chain-unsaturated-acyl-CoA dehydrogenase (molecular chaperone of complex I)	FAD
Long-and branched-chain-acyl-CoA dehydrogenase	FAD
C22-long-chain-acyl-CoA dehydrogenase	FAD
D-aspartate oxidase	FAD
L-amino acid oxidase	FAD
D-amino acid oxidase	FAD
Monoamine oxidase	FAD
Pyridoxal 5’-phosphate oxidase (Pyridoxine 5’-phosphate oxidase)	FMN
Catecholamine oxidase (renalase)	FAD
Methylenetetrahydrofolate reductase	FAD
L-pipecolate oxidase	FAD
Spermine oxidase	FAD
Electron-transferring flavoprotein-ubiquinone oxidoreductase	FAD
Electron transferring flavoprotein	FAD
Sarcosine dehydrogenase	FAD
Dimethylglycine dehydrogenase	FAD
Lysine-specific histone demethylase	FAD
Proline dehydrogenase	FAD
Cytochrome-b5 reductase	FAD
NADPH-hemoprotein reductase (cytochrome P450 reductase)	FMN, FAD
NAD(P)H dehydrogenase (quinone)	FAD
NADH-ubiquinone oxidoreductase of complex I, subunit UQOR1	FMN
NADPH-dependent diflavin oxidoreductase 1	FMN, FAD
tRNA dihydrouridine synthase	FMN
Dihydrolipoyl dehydrogenase	FAD
Glutathione-disulfide reductase	FAD
Thioredoxin-disulfide reductase	FAD
ER flavoprotein associated with degr.	FAD
Sulfhydryl oxidase	FAD
Prenylcysteine oxidase	FAD
Ribosyldihydronicotinamide dehydrogenase	FAD
Flavin-containing monooxygenases	FAD
Kynurenine 3-monooxygenase	FAD
Nitric-oxide synthase	FMN, FAD
Squalene monooxygenase	FAD
Monooxygenase in coenzyme Q biosynthesis	FAD
Ferrireductase (biliverdin IX beta reductase)	FMN
Methionine synthase reductase	FMN
Ferredoxin-NADP+ reductase	FAD
NAD(P)H oxidase cytochrome b(558), beta subunit	FAD
Thyroid oxidase / dual oxidase	FAD
Acetolactate synthase-like protein	FAD
Alkyldihydroxyacetone phosphate synthase	FAD
4’-Phosphopantothenoylcysteine decarboxylase	FMN
Cryptochrome	FAD
Apoptosis inducing protein	FAD
Apoptosis inducing protein	6-OH-FAD
Iodotyrosine deiodinase	FMN
Axon guidance protein Interacting with CasL	FAD
FAD-dependent oxidoreductase (molecular chaperone of complex 1)	FAD

Data for this table are from [39].

Table S3. Average recommended intake of pantothenic acid for different age groups

Birth to 6 months	1.7 mg/day
Infants 7-12 months	1.8 mg/day
Children 1-3 years	2 mg/day
Children 4-8 years	3 mg/day
Children 8-13 years	4 mg/day
Adolescent 14- 18 years	5 mg/day
Adult 19- 50 years	5 mg/day
51 years and older	5 mg/day
Pregnancy	6 mg/day
Lactation	7 mg/day

Data are from [40]

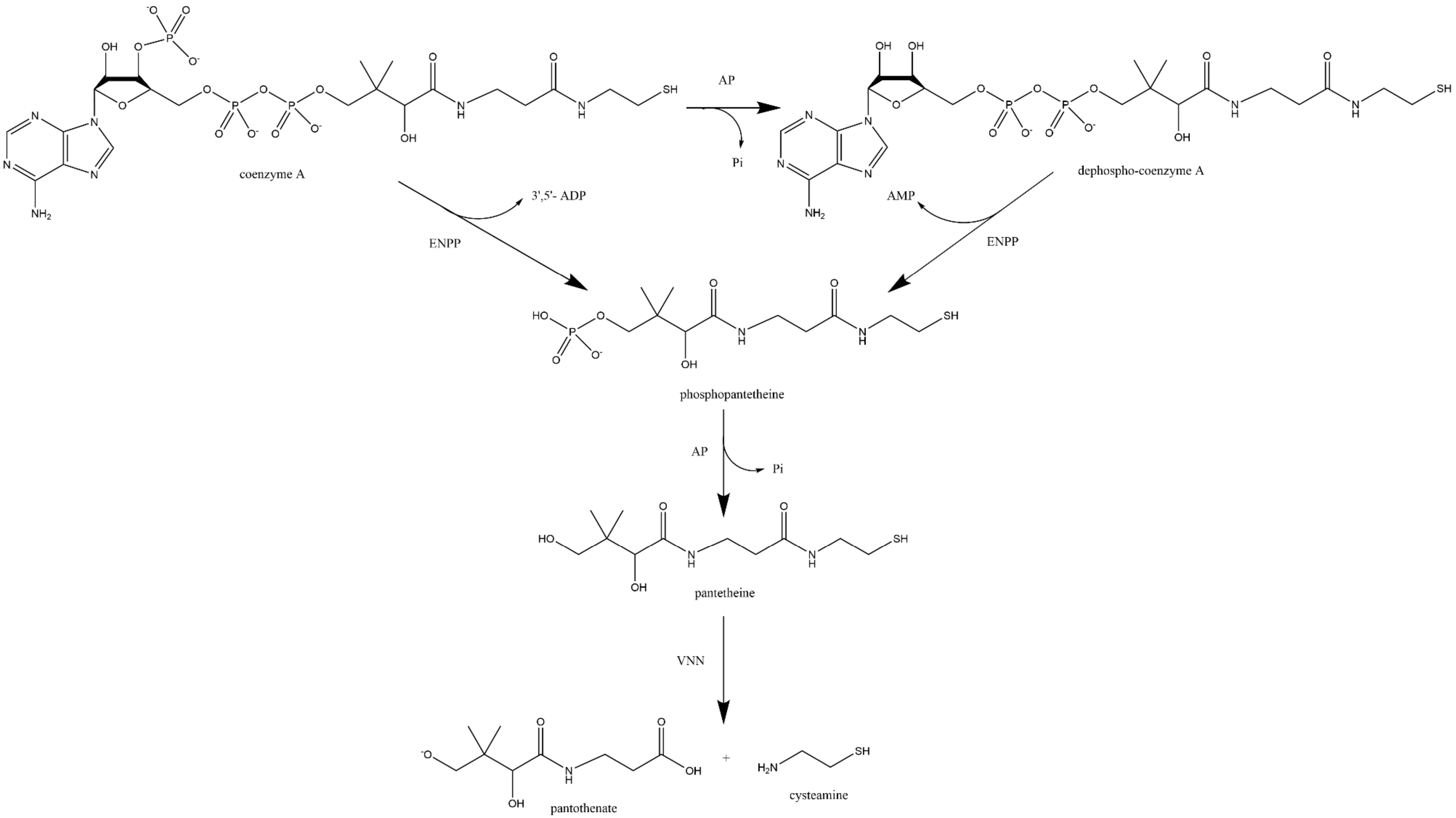

Figure S1. Hydrolytic reactions leading to release of vitamin B_5. AP, alkaline phosphatase; ENPP, ectonucleotide pyrophosphatase/phosphodiesterase; VNN, vascular non-inflammatory molecule (vanin).

References

1. Hampel, D.; York, E.R.; Allen, L.H. Ultra-performance liquid chromatography tandem mass-spectrometry (UPLC-MS/MS) for the rapid, simultaneous analysis of thiamin, riboflavin, flavin adenine dinucleotide, nicotinamide and pyridoxal in human milk. *J Chromatogr B Analyt Technol Biomed Life Sci* **2012**, *903*, 7-13, doi:10.1016/j.jchromb.2012.06.024.
2. Roelofsen-de Beer, R.; van Zelst, B.D.; Wardle, R.; Kooij, P.G.; de Rijke, Y.B. Simultaneous measurement of whole blood vitamin B1 and vitamin B6 using LC-ESI-MS/MS. *J Chromatogr B Analyt Technol Biomed Life Sci* **2017**, *1063*, 67-73, doi:10.1016/j.jchromb.2017.08.011.
3. Cheng, X.; Ma, D.; Fei, G.; Ma, Z.; Xiao, F.; Yu, Q.; Pan, X.; Zhou, F.; Zhao, L.; Zhong, C. A single-step method for simultaneous quantification of thiamine and its phosphate esters in whole blood sample by ultra-performance liquid chromatography-mass spectrometry. *J Chromatogr B Analyt Technol Biomed Life Sci* **2018**, *1095*, 103-111, doi:10.1016/j.jchromb.2018.07.030.
4. Ren, X.N.; Yin, S.A.; Yang, Z.Y.; Yang, X.G.; Shao, B.; Ren, Y.P.; Zhang, J. Application of UPLC-MS/MS Method for Analyzing B-vitamins in Human Milk. *Biomed Environ Sci* **2015**, *28*, 738-750, doi:10.3967/bes2015.104.
5. Verstraete, J.; Stove, C. Patient-Centric Assessment of Thiamine Status in Dried Blood Volumetric Absorptive Microsamples Using LC-MS/MS Analysis. *Anal Chem* **2021**, *93*, 2660-2668, doi:10.1021/acs.analchem.0c05018.
6. Kahoun, D.; Fojtíková, P.; Vácha, F.; Nováková, E.; Hypša, V. Development and validation of an LC-MS/MS method for determination of B vitamins and some its derivatives in whole blood. *bioRxiv* **2021**, doi:10.1101/2021.01.18.427110.
7. Armah, S.; Ferruzzi, M.G.; Gletsu-Miller, N. Feasibility of Mass-Spectrometry to Lower Cost and Blood Volume Requirements for Assessment of B Vitamins in Patients Undergoing Bariatric Surgery. *Metabolites* **2020**, *10*, doi:10.3390/metabo10060240.
8. Diniz, M.; Dias, N.; Andrade, F.; Paulo, B.; Ferreira, A. Isotope dilution method for determination of vitamin B2 in human plasma using liquid chromatography-tandem mass spectrometry. *J Chromatogr B Analyt Technol Biomed Life Sci* **2019**, *1113*, 14-19, doi:10.1016/j.jchromb.2019.03.001.
9. Jeong Hyeon, M.; Shin Beom, S.; Shin, S. Liquid Chromatography-Tandem Mass Spectrometry Analysis of Riboflavin in Beagle Dog Plasma for Pharmacokinetic Studies. *Mass Spectrom. Lett.* **2020**, *11*, 10-14, doi:10.5478/MSL.2020.11.1.10.
10. Meisser Redeuil, K.; Longet, K.; Benet, S.; Munari, C.; Campos-Gimenez, E. Simultaneous quantification of 21 water soluble vitamin circulating forms in human plasma by liquid chromatography-mass spectrometry. *J Chromatogr A* **2015**, *1422*, 89-98, doi:10.1016/j.chroma.2015.09.049.
11. Khaksari, M.; Mazzoleni, L.; Ruan, C.; Song, P.; Hershey, N.; Kennedy, R.; Burns, M.; Minerick, A. Detection and quantification of vitamins in microliter volumes of biological samples by Lc MS for clinical screening. *Aiche Journal* **2018**, *64*, 3709-3718, doi:10.1002/aic.16345.
12. Bishop, A.M.; Fernandez, C.; Whitehead, R.D., Jr.; Morales, A.P.; Barr, D.B.; Wilder, L.C.; Baker, S.E. Quantification of riboflavin in human urine using high performance liquid chromatography-tandem mass spectrometry. *J Chromatogr B Analyt Technol Biomed Life Sci* **2011**, *879*, 1823-1826, doi:10.1016/j.jchromb.2011.04.032.
13. Zhang, Q.; Ford, L.A.; Goodman, K.D.; Freed, T.A.; Hauser, D.M.; Conner, J.K.; Vroom, K.E.; Toal, D.R. LC-MS/MS method for quantitation of seven biomarkers in human plasma for the assessment of insulin resistance and impaired glucose tolerance. *J Chromatogr B Analyt Technol Biomed Life Sci* **2016**, doi:10.1016/j.jchromb.2016.10.025.
14. Jenčo, J.; Krčmová, L.K.; Sobotka, L.; Bláha, V.; Solich, P.; Švec, F. Development of novel liquid chromatography method for clinical monitoring of vitamin B1 metabolites and B6 status in the whole blood. *Talanta* **2020**, *211*, 120702, doi:10.1016/j.talanta.2019.120702.

15. Mathew, E.M.; Sakore, P.; Lewis, L.; Manokaran, K.; Rao, P.; Moorkoth, S. Development and validation of a dried blood spot test for thiamine deficiency among infants by HPLC-fluorimetry. *Biomed Chromatogr* **2019**, *33*, e4668, doi:10.1002/bmc.4668.
16. Huang, Y.; Gibson, R.A.; Green, T.J. Measuring thiamine status in dried blood spots. *Clin Chim Acta* **2020**, *509*, 52-59, doi:10.1016/j.cca.2020.06.011.
17. Nguyen, V.L.; Darman, M.; Ireland, A.; Fitzpatrick, M. A high performance liquid chromatography fluorescence method for the analysis of both pyridoxal-5-phosphate and thiamine pyrophosphate in whole blood. *Clin Chim Acta* **2020**, *506*, 129-134, doi:10.1016/j.cca.2020.03.026.
18. Petteys, B.J.; Frank, E.L. Rapid determination of vitamin B_2 (riboflavin) in plasma by HPLC. *Clin Chim Acta* **2011**, *412*, 38-43, doi:10.1016/j.cca.2010.08.037.
19. Stuetz, W.; Carrara, V.I.; McGready, R.; Lee, S.J.; Biesalski, H.K.; Nosten, F.H. Thiamine diphosphate in whole blood, thiamine and thiamine monophosphate in breast-milk in a refugee population. *PLoS One* **2012**, *7*, e36280, doi:10.1371/journal.pone.0036280.
20. Mandal, S.M.; Mandal, M.; Ghosh, A.K.; Dey, S. Rapid determination of vitamin B2 and B12 in human urine by isocratic liquid chromatography. *Anal Chim Acta* **2009**, *640*, 110-113, doi:10.1016/j.aca.2009.03.009.
21. Heydari, R.; Elyasi, N.S. Ion-pair cloud-point extraction: a new method for the determination of water-soluble vitamins in plasma and urine. *J Sep Sci* **2014**, *37*, 2724-2731, doi:10.1002/jssc.201400642.
22. Song, Z.; Hou, S. Determination of picomole amounts of thiamine through flow-injection analysis based on the suppression of luminol-KIO(4) chemiluminescence system. *J Pharm Biomed Anal* **2002**, *28*, 683-691, doi:10.1016/s0731-7085(01)00655-0.
23. Shankar, S.; John, S.A. Sensitive and highly selective determination of vitamin B1 in the presence of other vitamin B complexes using functionalized gold nanoparticles as fluorophore. *Rsc Advances* **2015**, *5*, 49920-49925, doi:10.1039/c5ra09165a.
24. Prasad, B.B.; Singh, R.; Singh, K. Development of highly electrocatalytic and electroconducting imprinted film using Ni nanomer for ultra-trace detection of thiamine. *Sensors and Actuators B-Chemical* **2017**, *246*, 38-45, doi:10.1016/j.snb.2017.02.033.
25. Zhang, H.; Chen, H.; Li, H.; Pan, S.; Ran, Y.; Hu, X. Construction of a novel turn-on-off fluorescence sensor used for highly selective detection of thiamine via its quenching effect on o-phen-Zn(2+) complex. *Luminescence* **2018**, *33*, 1128-1135, doi:10.1002/bio.3519.
26. Asgharian Marzabad, M.; Jafari, B.; Norouzi, P. Determination of Riboflavin by Nanocomposite Modified Carbon Paste Electrode in Biological Fluids Using Fast Fourier Transform Square Wave Voltammetry. *Int. J. Eng* **2020**, *33*, 1696-1702, doi:10.5829/ije.2020.33.09c.01.
27. Tyśkiewicz, K.; Dębczak, A.; Gieysztor, R.; Szymczak, T.; Rój, E. Determination of fat- and water-soluble vitamins by supercritical fluid chromatography: A review. *J Sep Sci* **2018**, *41*, 336-350, doi:10.1002/jssc.201700598.
28. Taguchi, K.; Fukusaki, E.; Bamba, T. Determination of niacin and its metabolites using supercritical fluid chromatography coupled to tandem mass spectrometry. *Mass Spectrom (Tokyo)* **2014**, *3*, A0029, doi:10.5702/massspectrometry.A0029.
29. ImmundiagnostikAG. ID-Vit® Pantothenic acid. Available online: https://www.immundiagnostik.com/media/pages/testkits/kif004/1c6c7f961a-1633917660/kif004_2019-05-23_pantothensaeure.pdf (accessed on 10.7.2021).
30. ImmundiagnostikAG. ID-Vit® Niacin. Available online: https://www.immundiagnostik.com/media/pages/testkits/kif003/2d1c628e3b-1633917660/kif003_2019-05-23_niacin.pdf (accessed on 10.7.2021).
31. RECIPEChemicals+InstrumentsGmbH. VITAMIN B1, B2 AND B6 (COMBIKIT). Available online: https://recipe.de/products/combikit-vitamin-b1-b2-b6-whole-blood/ (accessed on 10.7.2021).
32. ImmundiagnostikAG. Vitamin B1 HPLC Kit. Available online: https://www.immundiagnostik.com/media/pages/testkits/kc2201/59011e2c72-1633658467/vitamin-b1_kc2201.pdf (accessed on 10.7.2021).
33. RECIPEChemicals+InstrumentsGmbH. VITAMIN B1. Available online: https://recipe.de/products/vitamin-b1-whole-blood/ (accessed on 10.7.2021).
34. RECIPEChemicals+InstrumentsGmbH. VITAMIN B2. Available online: https://recipe.de/products/vitamin-b2-whole-blood/ (accessed on 10.7.2021).

35. RECIPEChemicals+InstrumentsGmbH. VITAMINS B1, B2, B6. Available online: https://recipe.de/products/vitamins-b1-b2-b6-in-whole-blood/ (accessed on 10.7.2021).
36. LSBio. Vitamin B2 / Riboflavin (Competitive EIA) ELISA Kit - LS-F55485. Available online: https://www.lsbio.com/elisakits/vitamin-b2-riboflavin-competitive-eia-elisa-kit-ls-f55485/55485 (accessed on 10.7.2021).
37. AntibodiesonlineGmbH. Vitamin B2 (Riboflavin) ELISA Kit. Available online: https://www.antibodies-online.com/kit/1059863/Vitamin+B2+Riboflavin+ELISA+Kit/ (accessed on 10.7.2021).
38. MYBioSource. Thiamine elisa kit :: Human Thiamine ELISA Kit. Available online: https://www.mybiosource.com/human-elisa-kits/thiamine/167383 (accessed on 10.7.2021).
39. Lienhart, W.D.; Gudipati, V.; Macheroux, P. The human flavoproteome. *Arch Biochem Biophys* **2013**, *535*, 150-162, doi:10.1016/j.abb.2013.02.015.
40. Institute of Medicine (US) Standing Committee on the Scientific Evaluation of Dietary Reference Intakes and its Panel on Folate, O.B.V., and Choline. Dietary Reference Intakes for Thiamin, Riboflavin, Niacin, Vitamin B6, Folate, Vitamin B12, Pantothenic Acid, Biotin, and Choline. In *Dietary Reference Intakes for Thiamin, Riboflavin, Niacin, Vitamin B6, Folate, Vitamin B12, Pantothenic Acid, Biotin, and Choline*; The National Academies Collection: Reports funded by National Institutes of Health; National Academies Press (US), National Academy of Sciences.: Washington (DC), 1998.
